# Cellulose nanofiber-enabled MXene textronics for wearable therapeutic applications

**DOI:** 10.1039/d6ra05144h

**Published:** 2026-10-30

**Authors:** Gharieb S. El-Sayyad, Ghadah Alrabghi, Ahmed Siddiq, Mohamed Mohamady Ghobashy

**Affiliations:** a Department of Biology, College of Science, Imam Mohammad Ibn Saud Islamic University (IMSIU) Riyadh 11623 Saudi Arabia gelsayyd@imamu.edu.sa; b Department of Physical Science, Faculty of Science, University of Jeddah Jeddah 21959 Saudi Arabia; c Department of Chemistry, Faculty of Science, Al-Azhar University Assiut 71524 Egypt; d Radiation Research of Polymer Chemistry Department, National Center for Radiation Research and Technology (NCRRT), Egyptian Atomic Energy Authority (EAEA) P.O. Box. 29, Nasr City Cairo Egypt Mohamed_ghobashy@yahoo.com Mohamed.ghobashy@eaea.org.eg

## Abstract

Electronic textiles (e-textiles) are advancing wearable healthcare by integrating electronics into soft, breathable, and scalable fabrics. Composites of cellulose nanofibers (CNFs) and two-dimensional MXenes have gained attention due to their complementary properties. CNFs offer flexibility, mechanical strength, biodegradability, and rich surface chemistry, while MXenes provide high conductivity and tunable multifunctionality. Strong interfacial interactions enable the development of durable, flexible, and environmentally compatible textronic architectures for wearable applications. This review examines the structural role of CNFs in MXene-based textronics across hierarchical formats, from fibers to fabrics. Key fabrication strategies, coating, spinning, electrospinning, and hybrid assembly, are discussed, with emphasis on comfort and performance balance. CNF–MXene textiles enable a range of functions, including wearable energy storage (supercapacitors), physiological and strain/pressure sensing, electrical stimulation, thermotherapy, and controlled drug delivery, positioning them as multifunctional platforms for both power-supporting and therapeutic wearable technologies. Key challenges, such as durability, washability, moisture resistance, and stability, are assessed, with future directions focusing on MXene stability, scalable production, and sustainable end-of-life strategies.

## Abstract 2

Cellulose nanofiber MXene textronics integrate sustainability, conductivity and multifunctionality, enabling advanced wearable sensors and therapeutic systems for next-generation healthcare applications.

## Graphical abstract

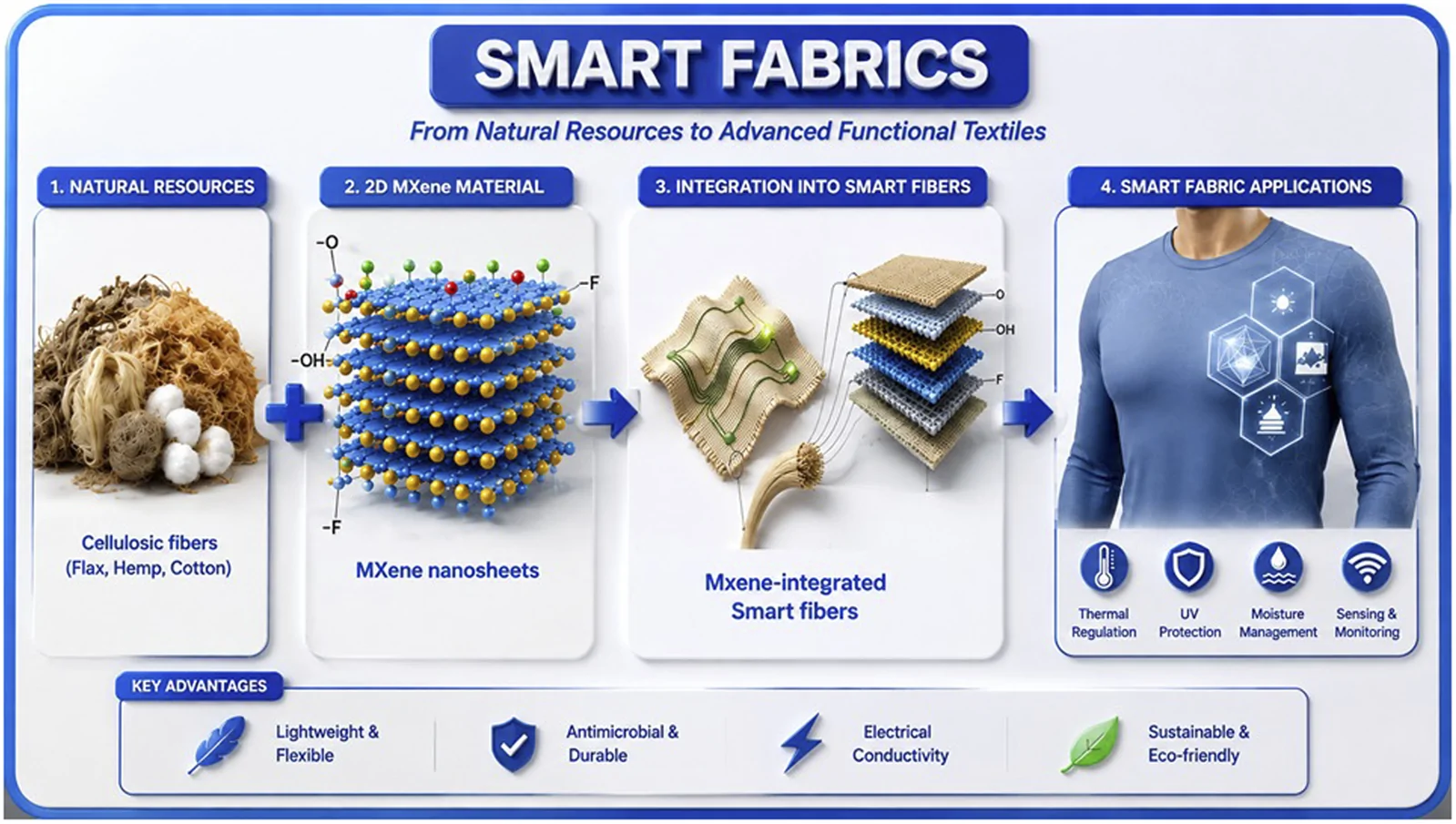

## 1. Introduction

Cellulose nanofibers (CNF) have emerged as promising materials for the development of electronic textiles (e-textiles) due to their unique combination of mechanical strength, flexibility, biodegradability, and excellent surface properties. In recent years, rapid advances in wearable technology have driven significant growth in research. Keum *et al.*^1^ reported that the innovation of wearable electronic devices has changed our lifestyles due to their appealing applications in artificial intelligence,^2^ human motion detection,^3^ robotics,^4^ energy storage systems,^5^ human health monitoring,^6^ and many more. Wearable electronic devices possess excellent intrinsic properties, including light weight, flexibility, and ease of attachment to human skin, which has prompted scientists to develop new generations of smart wearable devices.

Impressively, textile substrates such as fiber, yarn, or fabric possess all these superior characteristics. Along with these properties, textile materials exhibit outstanding wash fastness and physio-comfort features such as air permeability and sweat absorption. Therefore, textile substrates are now widely used in the development of wearable electronic devices. However, textiles are insulating materials, necessitating surface modification to prepare electronic textiles, also called textronics. Regarding this modification, various conductive materials are used, for example, graphene,^7,8^ carbon nanotube (CNT),^9^ boron nitride,^10^ metallic nanoparticles,^11^ conducting polymers,^12,13^ and more recently, two-dimensional (2D) transition metal carbides and nitrides known as MXene nanomaterials.^14–17^

Several recent review articles have summarized the development of MXene-based electronic textiles, conductive textile platforms, cellulose nanofiber composites, and flexible wearable bioelectronics. These studies have primarily focused on conductive nanomaterials, fabrication strategies, energy storage, sensing performance, or the general properties of cellulose-based functional materials. However, a comprehensive review specifically addressing the synergistic integration of cellulose nanofibers and MXenes for wearable therapeutic textronics remains lacking. In particular, limited attention has been given to the multifunctional structural role of cellulose nanofibers in improving MXene stability, mechanical robustness, flexibility, washability, and biocompatibility while simultaneously enabling therapeutic functions such as electrical stimulation, thermotherapy, physiological monitoring, and controlled drug delivery. The present review bridges this gap by integrating recent advances in material design, hierarchical textile architectures, fabrication technologies, therapeutic applications, and future translational challenges into a unified framework. By providing this focused perspective, the review offers practical design principles for developing next-generation sustainable therapeutic e-textiles based on CNF-enabled MXene composites.

Fig. 1a schematically illustrates the broad therapeutic landscape enabled by textile-based electronic systems, organized into three main domains: clinical intervention, functional rehabilitation, and physiological modulation. Fig. 1b summarizes five stimulation modalities achievable through e-textiles: electrical, thermal, chemical, optical, and mechanical. Electrical stimulation supports neuromuscular activation, pain management, and rehabilitation *via* conformal textile electrodes.^18^ Thermal stimulation enables controlled heating for pain relief and enhanced circulation *via* textile-integrated Joule heaters. Chemical stimulation allows localized or sustained drug delivery through textile-based reservoirs or responsive release systems, particularly relevant for wound care and chronic therapy.^19^ Optical stimulation, enabled by light-emitting fibers and photonic elements, supports phototherapy and antimicrobial treatments. Mechanical stimulation, including haptic feedback, pressure therapy, and motion assistance, enhances proprioception and functional recovery.^20^

**Fig. 1:**
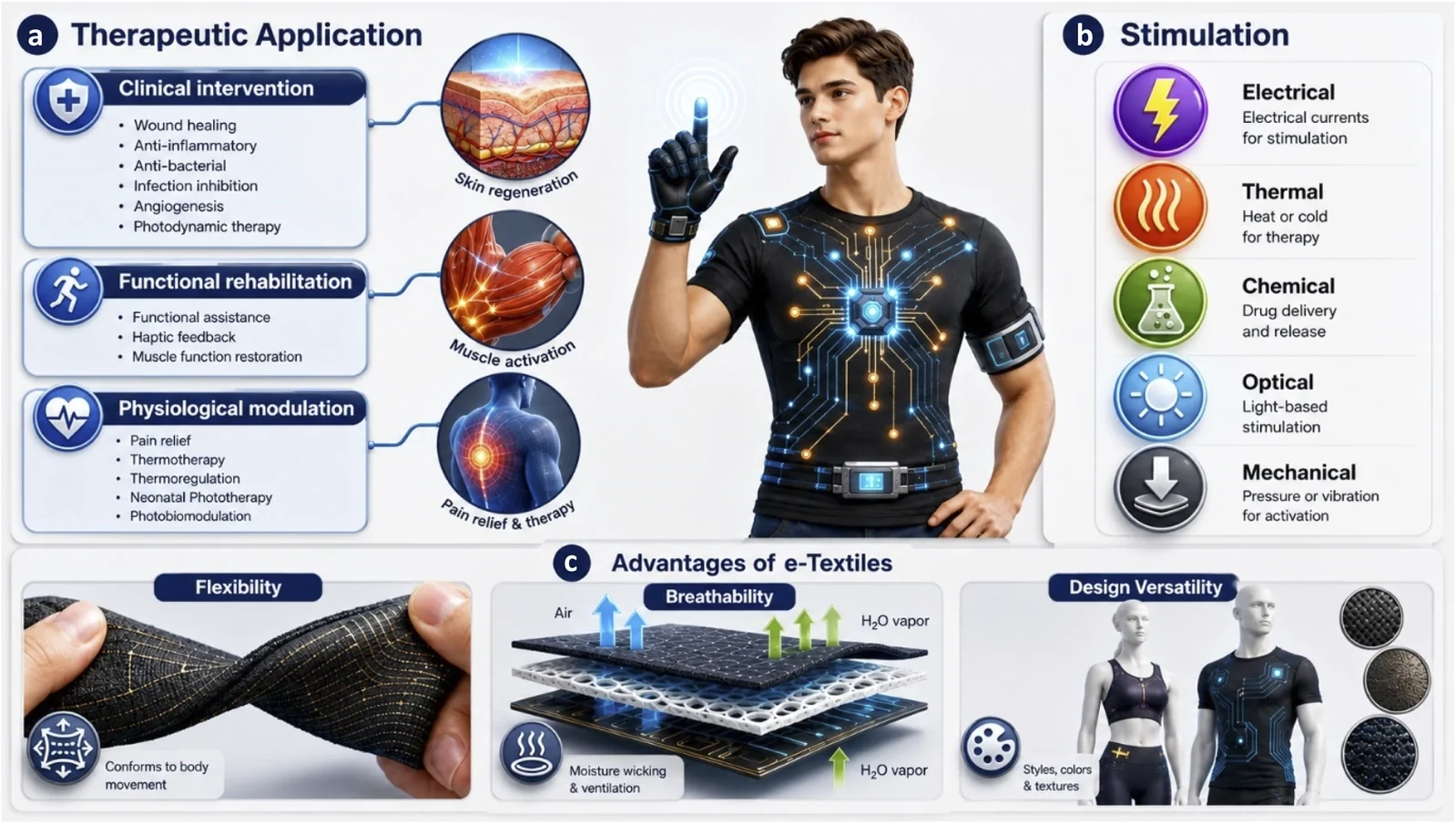
Schematic illustration of therapeutic e-textiles (a) textile-based therapeutic applications spanning clinical intervention, functional rehabilitation, and physiological modulation; (b) integrated stimulation modalities including electrical, thermal, chemical, optical, and mechanical; and (c) key enabling properties of e-textiles, flexibility, breathability, and scalable fabrication for comfortable, long-term wearable healthcare applications, this figure has been reproduced from ref. 28 with permission from nature, copyright 2025.

Fig. 1c highlights three key characteristics that make e-textiles particularly suitable for wearable therapeutics flexibility, breathability, and scalable fabrication. Textile flexibility ensures conformal skin contact, mechanical compliance, and comfort during prolonged wear, maintaining stable therapeutic and sensing performance. Breathability preserves air and moisture transport, reducing skin irritation and enabling long-term use in applications such as wound care and chronic pain management.^21,22^ Scalable fabrication using established textile processes, such as knitting, weaving, and embroidery, supports cost-effective manufacturing, design versatility, and the seamless integration of multiple functional elements into garments.^23^ Among these electroactive materials, cellulose nanofibers offer unique advantages as binders, structural reinforcements, and functional components in MXene-based textronics. The surface functional groups and hydrophilicity of both cellulose and MXene nanoflakes allow facile fabrication of composite materials.^24,25^ Cotton, for instance, has –OH functional groups which create strong adhesion bonds with MXene's functional groups through hydrogen bonding.^26,27^ Furthermore, the functional groups of textile substrates and MXenes enable additional surface modification, thereby enhancing the functional properties of MXene-based textronics. This review focuses on the role of cellulose nanofibers in the architectural design of MXene-based textronics and their applications in supercapacitors, thermotherapy, and sensors.

As summarized in Table 1, previous reviews have addressed MXene-based sensors, MXene energy-storage chemistry, general textile energy-harvesting devices, MXene/CNF fabrication processes, and therapeutic smart textiles as largely separate topics, with CNF typically treated as one interchangeable substrate among many rather than as a central material of interest. To our knowledge, no existing review has specifically consolidated the role of cellulose nanofibers, as a structural binder, reinforcing scaffold, and functional surface-chemistry partner, in MXene-based e-textiles across the full fiber-to-fabric hierarchy, while also connecting these structure–property relationships to therapeutic performance metrics (stimulation, thermotherapy, sensing, and drug delivery) and sustainability considerations. This review addresses that gap by synthesizing fabrication, durability, and mechanical/electrical performance data specifically through the lens of CNF–MXene interfacial chemistry, and by proposing design principles intended to guide future development of sustainable, biocompatible wearable therapeutic textiles.

**Table 1:** Comparison of the present review with existing reviews on MXene e-textiles, CNF composites, and wearable therapeutics

Prior review	Primary focus/scope	Limitations relative to present work	Ref.
Jin & Bai	MXene-based sensors (strain, pressure, gas, humidity) across general substrates	Focuses on sensing performance broadly; does not address CNF as a structural/binder material or therapeutic (stimulation, thermotherapy, drug-delivery) applications	15
Zhang & Nicolosi; Tang *et al.*	MXene applications in energy storage (supercapacitors, batteries)	Centered on electrochemical performance and materials chemistry of MXene electrodes generally; textile substrates and CNF interactions are only tangentially discussed	29 and 30
Chen *et al.*	Textile-based energy-harvesting and storage devices (general e-textiles)	Broad survey of triboelectric, piezoelectric, and supercapacitor textiles; does not focus on MXene–CNF interfacial chemistry or therapeutic use cases	31
Usman *et al.*	Fabrication techniques for MXene-based fibers and textiles (spinning, coating)	Covers processing methods comprehensively but treats CNF as one of several possible binders/substrates rather than as a central organizing material; therapeutic applications not addressed	32
Bhardwaj & Kundu	Electrospinning principles and nanofiber fabrication (general polymer science)	Foundational process-engineering review predating widespread MXene integration; no treatment of MXene, CNF-specific surface chemistry, or wearable therapeutics	33
Rohani Shirvan *et al.*	Wet spinning of advanced/functional fibers	Process-focused across many fiber chemistries; MXene/CNF systems are not a central theme, and therapeutic applications are outside its scope	34
Libanori *et al.*	Wearable and implantable bioelectronics for healthcare broadly	Covers therapeutic e-textiles at a conceptual/device level across many material systems; does not focus specifically on CNF or MXene, or on their interfacial binding mechanisms	35
Repon *et al.*	Therapeutic applications of smart textiles (general)	Surveys therapeutic textile functions broadly (stimulation, monitoring) without emphasis on specific nanomaterial systems such as MXene or CNF binders	36
Thakur *et al.*	MXene synthesis routes and effects of etching conditions on flake properties	Materials-synthesis focused; does not extend to textile integration, CNF interactions, or device-level therapeutic performance	37
**Present review**	CNF specifically as structural binder, reinforcement, and functional component in MXene-based textronics, spanning fiber–yarn–fabric hierarchy, fabrication methods, and therapeutic applications (stimulation, thermotherapy, sensing, drug delivery)	Consolidates CNF–MXene hydrogen-bonding mechanisms, fabrication trade-offs, and durability data into comparative tables; explicitly links CNF surface chemistry/mechanical properties to therapeutic device performance and sustainability	This work

## 2. Structural and functional properties of cellulose nanofibers

### 2.1. Structural characteristics

Cellulose nanofibers possess exceptional structural characteristics that make them ideal for electronic textile applications. CNF typically exhibits diameters ranging from 3 to 100 nanometers and lengths from several hundred nanometers to several micrometers, resulting in extremely high aspect ratios (length-to-diameter ratio). This nanoscale dimension results in a high surface area-to-volume ratio, providing abundant sites for functionalization and interaction with other materials.^38,39^ The crystalline structure of cellulose, particularly cellulose I, contributes to its high mechanical strength and chemical stability.

Fig. 2a shows the hierarchical structural design of textile platforms. The therapeutic functionality of e-textiles originates fundamentally from the hierarchical structure of textiles, which comprises three interconnected levels: fibers, yarns, and fabrics.^40^ Each structural level contributes distinct mechanical and functional properties that, when properly optimized and integrated, collectively determine the overall system's flexibility, durability, and capability to deliver therapeutic interventions. Understanding and optimizing this hierarchical architecture is essential for achieving reliable, long-term performance in wearable systems that must conform to the body's dynamic, complex surfaces and withstand the mechanical stresses imposed by daily wear and laundering.^41^

**Fig. 2:**
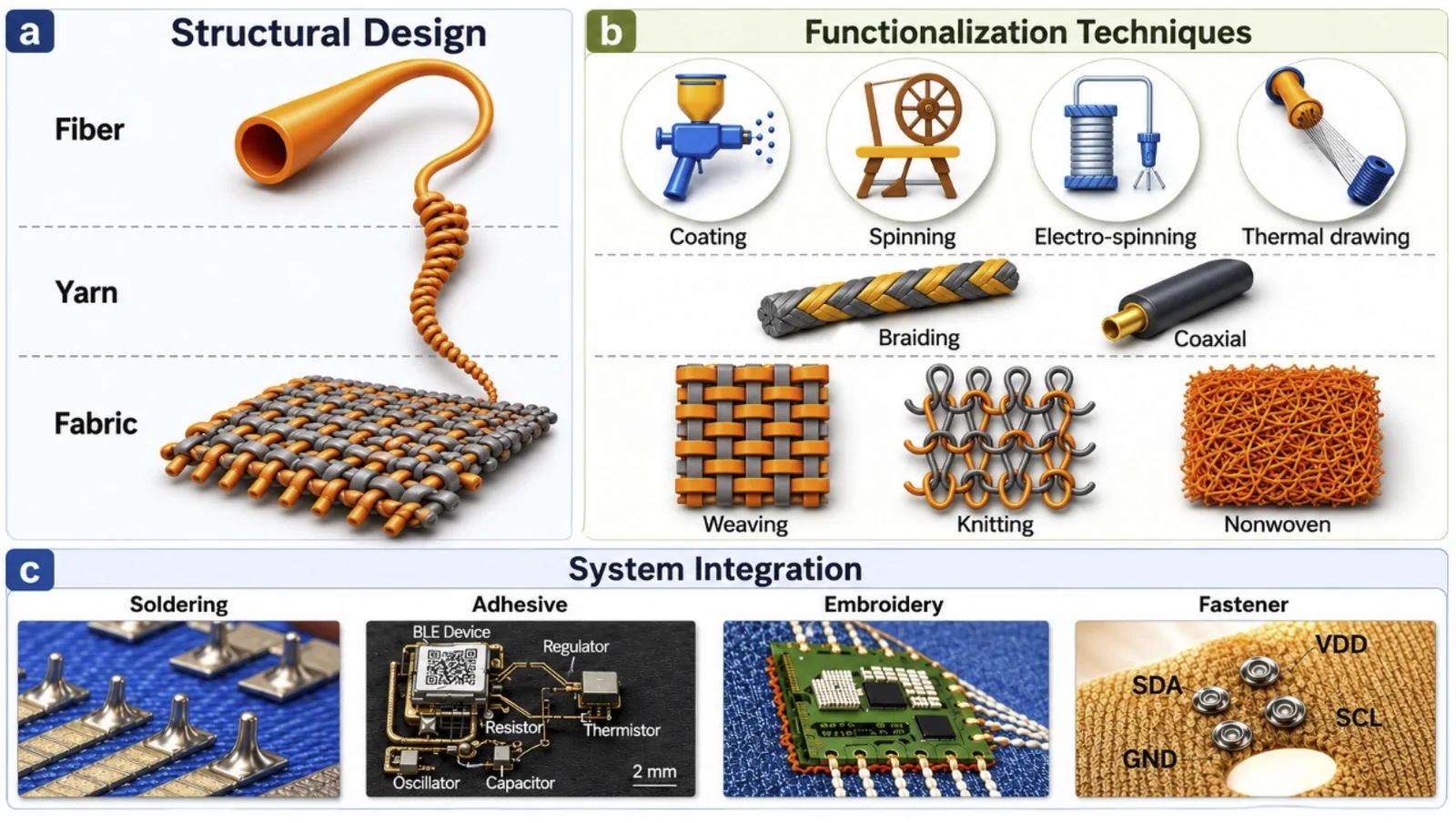
Structural architecture, functional modification approaches, and integration methodologies in therapeutic electronic textile systems. (a) Multilevel structural organization of textile platforms comprising three hierarchical components: fibers, yarns, and fabrics. Fibers serve as the primary structural units and can be assembled into yarns through processes such as braiding or coaxial configuration. These yarns are subsequently constructed into fabrics *via* weaving, knitting, or nonwoven fabrication, resulting in mechanically stable, breathable textile assemblies. (b) Functionalization strategies that impart therapeutic functionality, including surface coating, fiber spinning, electrospinning, and thermal drawing techniques. (c) Typical approaches for integrating electronic components within textile matrices, this figure has been reproduced from ref. 28 with permission from nature, copyright 2025.

At the fabric level, the organization of yarns through weaving, knitting, or nonwoven assembly determines the overall mechanical behavior, comfort characteristics, and therapeutic effectiveness of textile-based platforms. Building on the established properties of individual fibers and yarns, woven fabrics offer high dimensional stability and exceptional.^41^ This structural stability allows precise alignment of functional yarns while minimizing performance degradation under mechanical strain, making woven fabrics particularly suitable for integrating distributed electrodes or optical fibers to deliver localized stimulation and sensing across complex anatomical surfaces.^42^

Knitted fabrics, characterized by their looped architectural structures rather than interlaced threads, provide exceptional stretchability reaching up to 300% elongation^43^ and naturally conform to body contours, making them exceptionally well-suited for dynamic applications such as rehabilitation garments and adaptive thermotherapy devices.^43,44^ The inherent elasticity of knitted structures allows them to accommodate body movements, muscle expansion, and shape changes while maintaining electrical and functional contact with the body surface.

Nonwoven fabrics, formed through entanglement or bonding of fibers without interlacing or weaving, exhibit high porosity and expansive surface areas, supporting applications in breathable wound dressings and drug delivery platforms.^44,45^ The high porosity of nonwoven structures facilitates both drug diffusion and the permeability of moisture and gases, which are critical for supporting wound healing while maintaining breathability. For example, electrospun nonwoven mats loaded with insulin closed wounds approximately 17 percentage points faster compared to untreated controls, while enhancing key biomarkers associated with wound healing and promoting faster epithelial regeneration in both *in vitro* and *ex vivo* human skin models.^46^ The ability to tailor fabric architecture and material composition enables the creation of multifunctional textile platforms that can deliver mechanical resilience, electrical conductivity, and therapeutic functionality in a seamless, wearable format. Fig. 2b shows functionalization techniques enabling therapeutic capabilities.

The intrinsic softness and breathability of textile substrates make them ideal platforms for integrating functional materials and converting base textiles into active therapeutic devices. Building upon the hierarchical structure of fibers, yarns, and fabrics, effective integration of functional materials requires precise functionalization techniques that preserve mechanical flexibility and ensure user comfort. Among the available functionalization approaches, coating methods have emerged as particularly versatile and scalable strategies for imparting diverse therapeutic functionalities to textile platforms. Coating methods, including spray coating, dip coating, blade coating, and electrochemical deposition, are widely employed to impart conductivity, thermal responsiveness, optical emission, or controlled drug release capabilities directly onto textile surfaces while maintaining mechanical integrity and breathability.^47,48^ These approaches achieve uniform coatings across complex three-dimensional textile architectures while preserving the mechanical properties and flexibility of the underlying textile structure. Dip coating represents one of the most practical and scalable approaches for integrating functional materials into textile substrates. For instance, titanium carbide (Ti_3_C_2_T_*x*_) MXene dip-coatings can achieve surface conductivities exceeding 440 S cm^−1^ while retaining stable performance after 45 wash cycles.^49^ This level of durability and electrical conductivity is essential for reliable electrical stimulation and biosignal monitoring in wearable systems subjected to repeated laundering. Spray coating offers similar advantages, with enhanced flexibility for applying targeted coatings to specific regions of textiles, while blade coating provides highly controlled, uniform coating thickness. Electrochemical deposition enables precise control overcoating composition and microstructure, facilitating integration of catalytically active materials or responsive polymers.^50^ Beyond electrical performance, functional coatings can impart additional therapeutic functionalities such as stimuli-responsive drug delivery and thermal regulation to textile-based systems.^44,51–53^ For instance, hydrogel coatings embedded with drug reservoirs can release therapeutic agents in response to localized heating, typically in the range of 32–50 °C,^54–56^ or to electrical stimulation.^57^ The underlying mechanism involves thermally or electrically induced changes in the hydrogel network, such as polymer chain relaxation, matrix swelling, formation of microchannels, or polymer network collapse and dehydration, that facilitate precise diffusion of embedded therapeutic agents.^51,56^ These coatings have demonstrated rapid response times and sustained functionality across multiple activation cycles, delivering consistent therapeutic effects even under dynamic mechanical conditions.^58,59^ To balance functional performance with durability and wearer comfort, parameters including coating thickness uniformity and adhesion strength must be carefully optimized. Furthermore, nanomaterial-based coatings employing graphene oxide or carbon nanotube networks expand textile capabilities by providing high sensitivity for biosignal monitoring and efficient thermal dissipation, broadening the scope of multimodal therapeutic interventions.^60,61^ These advanced coatings integrate sensing, thermal management, and stimulation capabilities within a single textile platform, enabling simultaneous comprehensive therapeutic monitoring and intervention. Complementary to coating strategies, spinning techniques including melt spinning, wet spinning, dry spinning, and electrospinning enable the fabrication of continuous fibers with integrated functional properties essential for wearable therapeutic platforms. These techniques offer precise control over fiber composition, diameter, and internal structure, enabling creation of fibers optimized for specific therapeutic applications. Wet spinning represents a particularly effective approach for fabricating stretchable conductive fibers with high elongation capacity. Wet-spun fibers composed of thermoplastic polyurethane and nanosized carbon black can achieve elongations at break exceeding 2400% while maintaining electrical conductivities up to 210 S m^−1^, depending on carbon black loading fraction.^62^ These mechanical and electrical properties support both low-voltage stimulation and physiological sensing applications, providing remarkable compliance with body movements while maintaining reliable electrical function.

Electrospinning generates nanofiber mats with diameters of 200–500 nm, which facilitate exceptionally high drug-loading capacity and sustained release.^63^ These nanofiber mats have demonstrated controlled drug-delivery rates conducive to localized healing while maintaining permeability to moisture and gases, making them ideal for wound-dressing and transdermal-delivery applications.^45,64^ However, the structural robustness of electrospun nanofibers is typically lower than that of conventional spun fibers, rendering them susceptible to mechanical damage over extended use periods.^65^ To address this limitation, hybrid spinning approaches that integrate electrospun layers onto woven or knitted textile substrates have been developed.^66,67^ These composite structures enhance tensile strength and tear resistance by distributing mechanical stress across the supporting textile while preserving the nanofiber layer's breathability, flexibility, and high drug-loading capacity.

Thermal drawing offers a uniquely scalable and precise method for fabricating fibers with complex internal architectures, substantially expanding their functionality for therapeutic e-textile applications. This technique involves thermally softening preforms containing conductive, optical, or chemical materials, then drawing them into micro- or nanoscale fibers while preserving the intended internal structure.^64,68,69^ Thermally drawn multifunctional fiber probes can integrate optical waveguides, polymer electrodes, and microfluidic channels within a single filament, enabling simultaneous optogenetic stimulation, electrophysiological recording, and targeted drug delivery, supporting multifunctional therapeutic modalities with unprecedented integration.^70^ Thermal drawing can produce kilometers of such complex fibers with high uniformity, enabling mass production of large-area e-textile systems.^71,72^

However, maintaining both mechanical integrity and functional performance under dynamic conditions requires careful optimization of drawing parameters, including temperature, draw ratio, and drawing speed, as well as precise material selection.^73^ Optimizing polymer viscoelastic properties, such as by incorporating elastomers with high strain recovery, enhances the durability and stretchability of drawn fibers under repetitive deformation cycles.^74^ Moreover, controlling the core-to-shell ratio within the preform enables precise tuning of electrical conductivity and insulation properties, which are crucial for delivering consistent therapeutic effects in wearable platforms.^75^

Fig. 2c shows the system integration strategies for embedding electronics into textiles.

In addition to the challenges posed by mechanical stress and material compatibility in integrated systems, environmental factors, including moisture, sweat, and temperature fluctuations, introduce significant obstacles to maintaining long-term stability in wearable therapeutic applications. Moisture ingress can infiltrate soldered joints or adhesive layers, leading to oxidation, corrosion, or loss of adhesion, thereby compromising both electrical conductivity and mechanical integrity.^76,77^ Sweat introduces salts, enzymes, and organic compounds that accelerate these degradation processes,^78^ while temperature fluctuations can induce thermal expansion and contraction cycles, exacerbating interfacial stress and material fatigue.^79,80^ To mitigate these risks, advanced encapsulation strategies employing breathable yet waterproof membranes, such as fluorinated polymers or silicone elastomers, have been developed.^81–85^ These materials typically achieve water vapor transmission rates of 6.3–12.5 kg per m^2^ per day, providing a protective barrier against moisture ingress while maintaining wearer comfort and skin compatibility.

Moreover, the mechanical flexibility and conformability of these materials support dynamic movements without delamination or cracking, ensuring prolonged system durability and consistent therapeutic performance in real-world conditions. The hierarchical architecture of textiles from fibers through yarns to fabrics, when combined with precise functionalization techniques and optimized system integration strategies, enables the design of therapeutic systems that optimize mechanical compliance, durability, and seamless functional integration. By precisely controlling fiber material properties, including conductivity and elasticity, configuring yarn structures, such as core–sheath arrangements, to tailor mechanical and electrical performance, and selecting fabric constructions, including woven, knitted, or nonwoven variants, to match anatomical and therapeutic requirements, textile-based platforms can be engineered to meet specific clinical demands. This versatility supports diverse therapeutic modalities, including neuromodulation *via* integrated conductive pathways, localized thermal regulation *via* Joule-heating yarns, phototherapy *via* embedded optical fibers, and programmable drug delivery *via* electrospun nonwoven matrices. A deep understanding of textile hierarchy, combined with mastery of functionalization and integration techniques, is thus essential for developing reliable, effective, and user-friendly therapeutic systems capable of delivering continuous, adaptive healthcare interventions in accessible, comfortable, and scalable formats.

### 2.2. Surface chemistry of CNFs

The presence of hydroxyl groups (–OH) on the CNF surface facilitates strong interfacial bonding through hydrogen bonding interactions with various conductive materials, including MXene nanosheets. The interaction of MXene nanosheets and textile substrates mainly occurs through hydrogen bonding between the MXene surface functional groups and the substrate's polarity. Yang *et al.*^86^ demonstrated that to ensure good adhesion properties, cellulosic fabric or yarn surfaces are pretreated with acetone, ethanol, and NaOH to activate the functional groups. This pretreatment creates additional reactive sites that enhance bonding with conductive nanomaterials.

MXene nanoflakes are generally presented by the M_*n*+1_X_*n*_T_*x*_ formula, where T_*x*_ represents the surface functional groups such as –O, –OH, –F, and/or –Cl.^24,25^ Naguib and his co-workers^87^ first prepared Ti_3_C_2_ layered MXene with OH and/or F functional groups from Ti_3_AlC_2_ precursors using hydrofluoric (HF) acid, and to date, numerous MXene nanoflakes have been prepared, including Ti_2_C, V_2_C, Nb_2_C, Ti_4_N_3_, TiNbC, Ti_3_CN, Mo_2_TiC_2_, *etc.*^88,89^ The surface terminal groups of MXenes can form strong bonds with the textile substrate *via* hydrogen bonding, creating stable composite structures.

Sun *et al.*^90^ demonstrated the development of stable MXene/cellulose nanofiber (CNF) membranes for efficient osmotic energy generation. Pure MXene membranes, while promising for nanofluidic ion transport, suffer from poor mechanical stability in aqueous environments due to weak van der Waals interactions between nanosheets. To address this, the authors incorporated CNF as a renewable, mechanically robust binder. The resulting composite membrane exhibited significantly enhanced structural integrity, remaining intact in high-salinity solutions for over 15 days, unlike pure MXene membranes, which degraded under the same conditions. This stability is attributed to hydrogen bonding between the hydroxyl and carboxyl groups of CNF and the surface functional groups of MXene, as confirmed by X-ray photoelectron spectroscopy (XPS) peak shifts.^91,92^ Sun *et al.*^90^ also investigated key factors influencing performance. The power output varied with the type of electrolyte cation, following the order K^+^ > Na^+^ > Li^+^ > Mg^2+^ > Ca^2+^, which correlated with the ions' hydration diameters and diffusion coefficients. Smaller, faster-diffusing cations like K^+^ yielded higher power. Additionally, the power density was pH-dependent, increasing under alkaline conditions due to enhanced deprotonation of surface hydroxyl groups on CNF, thereby increasing the membrane's negative charge density and, consequently, its cation selectivity.^93,94^

Fig. 3a illustrates the schematic process for preparing delaminated MXene nanosheets, which serve as the foundational 2D material for the composite membrane. The process began with the MAX-phase precursor, titanium aluminum carbide (Ti_3_AlC_2_), which was chemically etched using a hydrochloric acid and lithium fluoride (HCl/LiF) solution. This selective etching removed the aluminum (Al) layers, expanding the layered structure and forming MXene (Ti_3_C_2_T_*x*_). The successful delamination was confirmed by X-ray diffraction (XRD), which showed the disappearance of characteristic MAX-phase peaks such as (004), (101), (104), and (105), along with a pronounced shift of the (002) peak from 9.08° to 5.02°. This shift indicates an increase in interlayer spacing due to the removal of Al and the introduction of surface functional groups (–F, –OH, –O), which are critical for subsequent interactions with cellulose nanofibers.

**Fig. 3:**
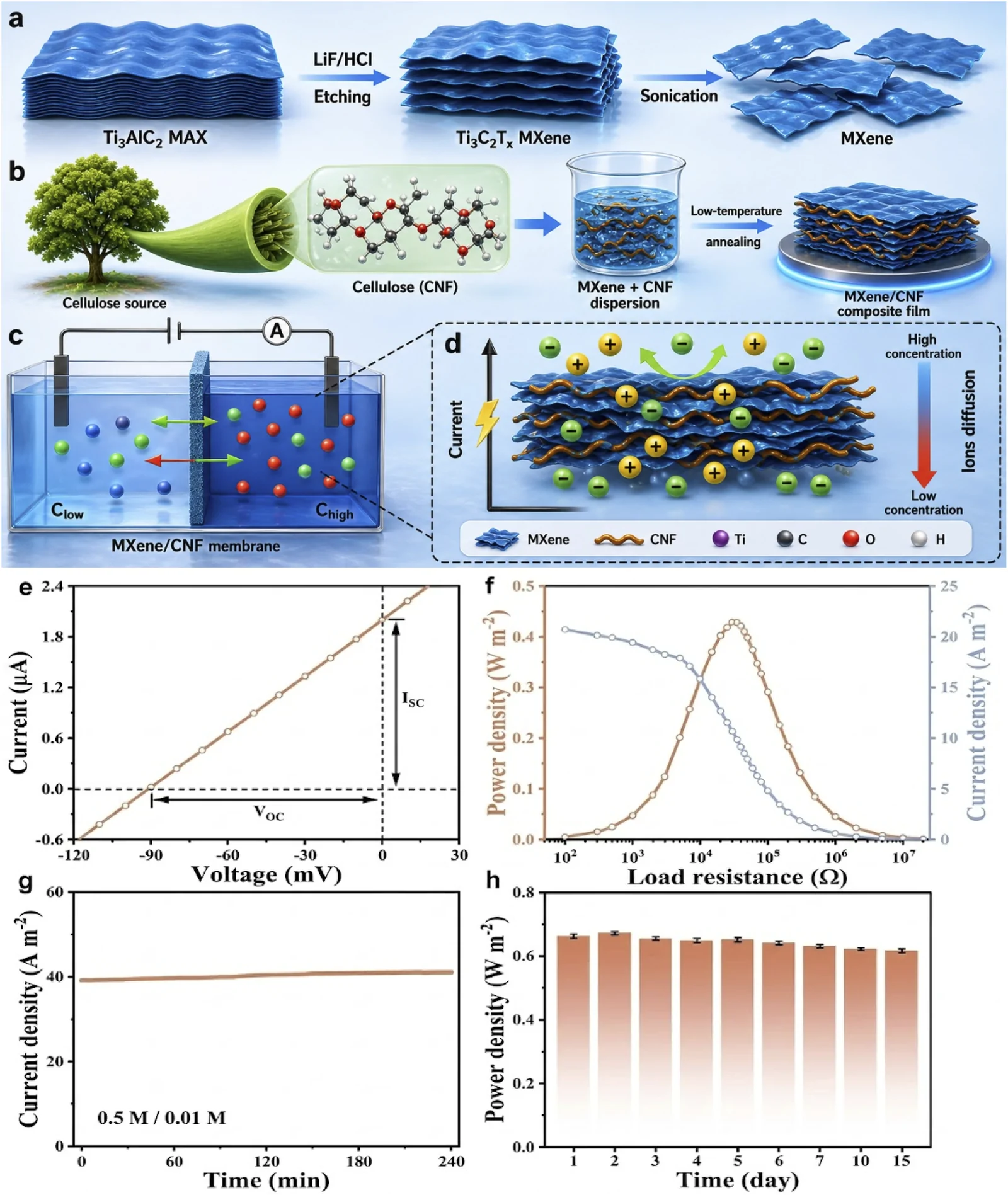
Fabrication, structure, and energy-harvesting performance of an MXene/CNF composite membrane. (a) Schematic illustration of MXene synthesis from Ti_3_AlC_2_ MAX phase *via* LiF/HCl etching and subsequent sonication to obtain Ti_3_C_2_T_*x*_ nanosheets. (b) Integration of cellulose nanofibers (CNFs) with MXene to form a homogeneous aqueous dispersion. (c) Layered architecture of the MXene/CNF composite film and its structural evolution after low-temperature annealing. (d) Working principle of the MXene/CNF membrane for osmotic energy conversion, showing ion transport across a salinity gradient and current generation using Ag/AgCl electrodes. (e) Current–voltage (*I*–*V*) characteristics of the membrane, indicating open-circuit voltage (*V*_oc_) and short-circuit current (*I*_sc_). (f) Output power density and current density as a function of external load resistance. (g) Long-term current stability under a 0.5 M/0.01 M concentration gradient. (h) Power density stability over 15 days, demonstrating durable energy-harvesting performance, this figure has been reproduced from ref. 95 with permission from Elsevier, copyright 2025.

Fig. 3b depicts the integration of cellulose nanofibers (CNF) with the exfoliated MXene nanosheets to form a stable, layered composite membrane. CNF was introduced as a renewable biomass-based binder to address the inherent mechanical instability of pure MXene membranes, which suffer from weak van der Waals interactions between nanosheets. The CNF fibers, rich in hydroxyl and carboxyl groups, formed hydrogen bonds with the functionalized MXene surfaces, significantly enhancing the composite's structural integrity and aqueous stability. This interaction was further verified through X-ray photoelectron spectroscopy (XPS), which showed shifts in the binding energies of C–Ti and C–O peaks, indicating strong interfacial hydrogen bonding. The resulting MXene/CNF dispersion exhibited excellent colloidal stability, as demonstrated by the Tyndall effect, and could be uniformly assembled into membranes *via* vacuum filtration.

Fig. 3c presents the final membrane structure and its functional advantages as a nanofluidic osmotic energy generator. The vacuum-filtrated MXene/CNF membrane exhibited a well-ordered, laminate architecture with an interlayer spacing that creates interconnected nanochannels for selective ion transport. The incorporation of CNF not only improved mechanical robustness allowing the membrane to remain intact in high-salinity solutions for over 15 days but also contributed additional negative surface charges, enhancing cation selectivity. This layered design facilitates efficient ion diffusion across salinity gradients, enabling the membrane to function as a high-performance, stable generator of osmotic energy. The membrane's enhanced hydrophilicity, reduced water contact angle, and ability to be fabricated as a free-standing film further support its practicality for scalable energy-harvesting applications.

Fig. 3d schematically illustrates the experimental setup used to evaluate the osmotic energy conversion performance of the MXene/CNF (MC) membrane. The setup consists of a two-compartment electrochemical cell, separated by the MC membrane, which creates a salinity gradient by holding different electrolyte concentrations on each side. The high concentration (HC) compartment represents the high-salinity source simulating seawater, while the low concentration (LC) compartment represents the low-salinity source simulating river water. A pair of Ag/AgCl electrodes is placed in each compartment to measure the transmembrane potential and current. The external circuit, connected to these electrodes, allows for both open-circuit voltage (*V*_oc_) and short-circuit current (*I*_sc_) measurements, as well as the application of an external load resistance to determine the maximum power output. This setup mimics a reverse electrodialysis (RED) configuration and directly assesses the membrane's ability to convert Gibbs free energy from a salt concentration gradient into usable electrical power.

Fig. 3e illustrates the practical application and long-term performance of the MXene/CNF (MC) membrane for osmotic energy conversion under simulated real-world conditions. Fig. 3e shows the current–voltage (*I*–*V*) curve of the MC membrane under a salinity gradient mimicking seawater and river water (0.5 M/0.01 M NaCl). The linear response confirms stable ion transport, with an open-circuit voltage (*V*_oc_) of −91 mV and a short-circuit current (*I*_sc_) of 2 µA. Fig. 3f plots the output power density and current density as a function of external load resistance under the same NaCl gradient. The power density peaks at approximately 0.43 W m^−2^ at a load resistance of ≈30 kΩ, demonstrating efficient energy extraction under realistic salinity differences. Fig. 3g presents the short-circuit current density of the MC membrane over time under a 0.5/0.01 M KCl gradient. The current remains stable for 4 hours without decay, indicating reliable, consistent ion flux during continuous operation. Fig. 3h highlights the long-term stability of the energy generator, showing that the power output remains nearly constant over 15 days of testing under the same KCl gradient. This exceptional durability confirms the structural and functional integrity of the MC membrane in prolonged osmotic energy harvesting applications.

### 2.3. Mechanical properties

Cellulose nanofibers exhibit outstanding mechanical properties, with tensile strengths ranging from 200 to 300 MPa and Young's moduli from 20 to 150 GPa, depending on the source and processing methods. These mechanical properties are critical for maintaining structural integrity in flexible electronic devices that undergo repeated bending, stretching, and washing cycles. 2D MXene nanosheets exhibit excellent electrical, mechanical, and thermal properties, as well as high-temperature and corrosion resistance, superior to those of other electroactive materials.^15,96^ Meng *et al.*^96^ reported that, due to their outstanding properties, research interest in these 2D nanomaterials has grown when combined with cellulosic substrates. These outstanding materials (CNFs) represent one of the most promising classes of functional materials for textile-based electronic systems, particularly due to their exceptional properties, including biocompatibility, sustainability, electrical modulation capability through coating and functionalization, and mechanical properties that rival or exceed those of synthetic polymers. The mechanical properties of cellulose nanofiber e-textiles fundamentally determine their suitability for sustainable, biocompatible wearable therapeutic applications where environmental impact and skin compatibility are important considerations.

Uzun *et al.*^23^ and Khudiyev *et al.*^97^ have used cellulose nanofibers as a distinct and increasingly important class of functional materials for textile-based electronic and therapeutic systems, fundamentally different from carbon nanotube fibers that have been discussed separately. Cellulose nanofibers derive from natural cellulose sources, including wood pulp and plant fibers, or from synthesized cellulose derivatives and offer unique combinations of properties, including biocompatibility, sustainability, electrical modulation capability through coating and functionalization, and exceptional mechanical properties that rival or exceed those of synthetic polymers. The mechanical properties of cellulose nanofiber e-textiles fundamentally determine their suitability for sustainable, biocompatible wearable therapeutic applications where environmental impact and skin compatibility are important considerations for long-term patient use and acceptance.

Uzun *et al.*^23^ have prepared knittable, washable, multifunctional MXene-coated cellulose yarns, which represent one of the most well-developed examples of cellulose nanofiber integration. These innovative yarns integrate MXene, a transition-metal carbide known as Ti_3_C_2_T_*x*_, onto cellulose yarn substrates, creating a hybrid system that combines the biocompatibility and sustainability of cellulose with MXene's electrical conductivity. These MXene-coated cellulose yarns achieve surface conductivities exceeding 440 siemens per centimeter while retaining stable performance after 45 wash cycles. The preservation of electrical conductivity after 45 wash cycles is remarkable. It indicates that the MXene coating adheres securely to the cellulose substrate, despite the mechanical and chemical stresses of repeated laundering, which would damage or degrade many alternative coating approaches.

Dong *et al.*^98^ have further developed cellulose nanofiber technology through sophisticated MXene-optimized films comprising a three-component composite of MXene, carbon nanotubes, and cellulose nanofibers on a stretchable thermoplastic polyurethane substrate. This represents a highly sophisticated approach in which cellulose nanofibers serve as a structural scaffold, contributing mechanical properties, while MXene and carbon nanotubes provide electrical conductivity and thermal responsiveness. The integration of three distinct functional materials into a unified mechanical structure exemplifies the versatility of cellulose nanofiber approaches for complex therapeutic applications.

Chen *et al.*^44^ have evaluated the mechanical and functional properties of cellulose nanofiber-based textiles, noting that nonwoven cellulose-based fabrics formed by fiber entanglement or bonding without interlacing exhibit high porosity and surface area. These properties directly support applications in breathable wound dressings and drug-delivery platforms, where maintaining moisture balance, gas exchange, and therapeutic agent delivery are essential for tissue regeneration. The high porosity serves dual functions in therapeutic applications, allowing the material to compress and deform easily without permanent damage while accommodating body motion and shape changes. Simultaneously, the high surface area and porosity enable drug loading, moisture transport, and breathability, which are critical properties for wound-healing applications.

Hashem *et al.*^63^ and Walther *et al.*^46^ have used electrospun nanofiber mats as the primary method for producing ultrafine cellulose nanofibers with controlled porosity and nanofiber diameter. The literature demonstrates electro-spun nanofiber mats with diameters as small as 200 to 500 nanometers, which facilitate high-capacity drug loading and sustained release. Bioactive insulin-loaded electro-spun wound dressings for localized drug delivery and stimulation of protein expression associated with wound healing represent a specific therapeutic application. These electro-spun mats demonstrate controlled drug-delivery rates conducive to localized healing while maintaining permeability to moisture and gases, making them ideal for wound dressings and transdermal delivery applications.

Oh *et al.*^65^ have addressed an important limitation of electro-spun cellulose nanofiber mats by noting that their structural robustness is typically lower than that of conventional spun fibers, rendering them susceptible to mechanical damage over extended use. This limitation has prompted researchers to develop innovative solutions. Jalalah *et al.*^66^ and Sanchaniya *et al.*^67^ have developed hybrid spinning approaches such as integrating electrospun layers onto woven textile substrates. These composite structures enhance tensile strength and tear resistance by distributing mechanical stress across the supporting textile while preserving the breathability and flexibility of the nanofiber layer. This hybrid approach embodies an important mechanical design principle: rather than aiming to create mechanically strong nanofiber mats, designers integrate fragile yet highly functional nanofiber layers with robust mechanical support layers. The supporting woven textile provides tensile strength and tear resistance, while the electro-spun nanofiber layer provides therapeutic functionality.

Zhang *et al.*^49^ have designed a sophisticated thermal therapy system based on a flexible MXene-decorated fabric with interwoven conductive networks for integrated Joule heating, electromagnetic interference shielding, and strain sensing. This system combines a cellulose-based fabric substrate, providing mechanical support and a base structure, with an MXene coating, providing electrical conductivity and thermal responsiveness, and an interwoven conductive network structure that distributes mechanical and thermal loads across the fabric. The resulting system achieves integrated Joule heating, electromagnetic interference shielding, and strain sensing, demonstrating that mechanical and functional properties can be simultaneously optimized in cellulose-based systems.

Cheng *et al.*^99^ have developed an advanced example of an MCCF- or MXene-optimized conductive film comprising a three-component composite system for wound-healing applications. This system was designed as a coaxially electrospun structure with a polycaprolactone shell encapsulating a hydroxypropyl cellulose core loaded with the antibiotic ciprofloxacin. The coaxial electrospinning approach leverages cellulose properties, with the PCL shell providing mechanical protection and an initial barrier function. In contrast, the hydroxypropyl cellulose core provides drug storage and a thermoresponsive release mechanism. Upon heating above 45 degrees Celsius, hydroxypropyl cellulose dehydrates and contracts, creating pressure that forces drug through microchannels in the polycaprolactone shell. The system demonstrated rapid heating at a low input voltage of approximately 4 volts. It retained mechanical integrity over 500 continuous bending cycles and in physiologically relevant corrosive environments, such as simulated sweat and body fluids.

Inman *et al.*^100^ have developed a woven cotton supercapacitor incorporating MXene as the active material, paired with a lithium chloride or polyvinyl alcohol gel electrolyte, in which cotton, composed primarily of cellulose fibers, serves as the mechanical substrate. Key mechanical properties documented include maintained flexibility, with the supercapacitor retaining mechanical compliance despite the integration of rigid electrochemical components, and consistent energy delivery over 71 pulsed cycles. Most impressively, the supercapacitor provided sufficient energy for continuous operation of a complete sensor system including wireless transmission for up to 96 minutes, demonstrating that cellulose-based mechanical substrates can support complex integrated electronic systems while maintaining their fundamental mechanical properties.

Wang *et al.*^101^ have designed helical core–sheath elastic yarn-based dual strain or humidity sensors with MXene sensing layers, emphasizing MXene coating rather than pure cellulose. In contrast, the core–sheath architecture leverages the mechanical properties of cellulose or cellulose-derived yarns. The elastic core provides mechanical compliance and strain response, while the MXene-coated sheath transduces mechanical deformation into electrical signal changes. The mechanical design enables simultaneous measurement of joint strain (representing body motion) and humidity (representing sweat), supporting comprehensive physiological monitoring in rehabilitation applications.

Li *et al.*^20^ reported that vibrotactile actuators constructed from textile materials achieved an air permeability of 108 liters per square meter per second, indicating that the material is highly porous and allows essentially unimpeded airflow, enabling extended wear without heat or moisture accumulation. Cellulose nanofiber-based materials inherently exhibit high air permeability due to their porous nanofiber structure. This property arises naturally from the electrospinning process, in which nanofibers are randomly arranged with large inter-fiber spaces. The mechanical consequence is that the material remains breathable even when incorporated into functional textile systems with coatings or functional components.

Wang *et al.*,^81^ Li *et al.*,^20^ and Sheng *et al.*^83^ have demonstrated advanced encapsulation materials with water vapor transmission rates ranging from 6.3 to 12.5 kilograms per square meter per day, providing a protective barrier against moisture ingress while maintaining wearer comfort and skin compatibility.

Future research directions for cellulose nanofiber e-textiles should focus on optimizing mechanical properties by developing hybrid structures that combine mechanical strength with therapeutic functionality. Durability enhancement through improving wash stability and long-term chemical or biological stability of coatings is necessary. Functional integration, advancing coating and functionalization techniques to achieve electrical, thermal, and therapeutic properties simultaneously, is important. Clinical validation through rigorous clinical studies demonstrating therapeutic efficacy and biocompatibility in real-world use is essential. Sustainability assessment through comprehensive life-cycle assessments documenting environmental benefits relative to synthetic alternatives should be conducted to fully demonstrate the advantages. The development of mechanically reliable and flexible substrates represents a critical advancement for next-generation deformable electronics, particularly for applications requiring sustained performance under cyclic mechanical and thermal stress. The cellulose nanofiber (CNF) hybrid films, fabricated *via* simultaneous electrospinning and spraying, exhibit exceptional mechanical properties that significantly outperform those of conventional plastic substrates used in flexible and stretchable electronics.

Fig. 4a and b together illustrate the critical microstructural evolution of the CNF–epoxy hybrid film induced by hot pressing. In Fig. 4a, SEM images (top and cross-sectional views) reveal the hybrid film immediately after simultaneous epoxy electrospinning and CNF spraying, prior to hot pressing, where a highly porous, fibrous architecture is observed with CNFs dispersed within a three-dimensional epoxy nanoweb; the surface is rough and the internal porosity is pronounced, reflecting a loosely integrated intermediate structure that promotes light scattering and limits mechanical integrity. After hot pressing at 95 °C under 10 MPa (Fig. 4b), the morphology transforms into a dense, smooth, and uniform film with substantially reduced porosity and surface roughness, achieving a root-mean-square roughness of 0.7 nm and a controlled thickness of 20 µm. This densification results from the melting and redistribution of epoxy nanofibers, which fill voids and strengthen interfacial bonding between the CNFs and the epoxy matrix. Consequently, hot pressing is essential for converting the initially porous hybrid into a continuous and thermally stable composite, thereby enabling enhanced optical transparency, improved mechanical strength and modulus, and reduced thermal expansion. The comparison between these two states highlights the pivotal role of post-processing in realizing the high-performance substrate properties reported in subsequent figures and in providing sufficient structural integrity for patterning and stretchable electronic applications. As illustrated in Fig. 4c, the stress–strain curves provide critical insights into the mechanical performance hierarchy of the films. The hybrid films exhibit Young's moduli of 5.5 GPa and tensile strengths of 190 MPa, which are more than threefold higher than those of pristine epoxy films (1.7 GPa and 40 MPa, respectively). More significantly, these values substantially exceed those of commonly used polymer substrates such as polyethylene terephthalate (PET; 2 GPa and approximately 50–60 MPa) and polyimide (2.5 GPa), demonstrating the superior structural integrity of the CNF hybrid system. In direct comparison, the pristine cellulose nanofiber film alone exhibits even higher modulus and strength values (6 GPa and 220 MPa), but introduces optical haze and water-compatibility issues. The hybrid composition therefore, represents an optimal balance between mechanical reinforcement from the high-modulus CNF network and the functional versatility provided by the epoxy matrix. The mechanical enhancement in the hybrid films can be attributed to the synergistic combination of the rigid CNF network and the ductile epoxy matrix. As the volume of sprayed CNF solution increases, the hybrid film exhibits significantly enhanced mechanical properties due to the reinforcing effect of the high-strength cellulose nanofibers, which possess individual Young's moduli exceeding those of aluminum and glass fibers. The epoxy backbone, while mechanically softer, provides the critical functions of binding the CNF fillers into a cohesive structure, enabling photolithographic patterning, and improving water resistance. This composite architecture creates a material with superior load-bearing capacity compared to single-component materials, making it exceptionally well-suited for demanding flexible electronics applications. Thermal stability is another critical factor in determining a substrate's suitability for electronic applications, as differences in coefficients of thermal expansion (CTE) between the substrate and device layers can induce catastrophic stress during fabrication and operation. As shown in Fig. 4d, the CTE analysis reveals a dramatic improvement in thermal properties of the hybrid films. While pristine epoxy films exhibit a CTE of 53 ppm K^−1^, which is far too high for thin-film electronics, the hybrid films formed by spraying 300 mL of CNF solution achieve a significantly reduced CTE of 10 ppm K^−1^. This remarkable reduction approaches that of quartz glass (<10 ppm K^−1^) and is substantially lower than conventional plastic substrates such as PET and polyimide (both >50 ppm K^−1^). The low CTE of the hybrid films is achieved because thermal stress in the epoxy is largely suppressed by the CNF network, which itself exhibits low thermal expansion due to its crystalline cellulose structure. To validate the practical implications of this low CTE, the authors conducted a temperature-humidity bias test in which chemically vapor-deposited graphene layers were transferred onto both PET and CNF hybrid films and exposed to 85 °C and 85% relative humidity for 240 hours. The results were striking: graphene on the PET film showed significant changes in sheet resistance due to the PET substrate's large thermal expansion, leading to crack formation in the graphene layer. In contrast, the sheet resistance of graphene on the hybrid film changed negligibly, directly demonstrating the thermomechanical stability advantage provided by the CNF hybrid's low CTE. This compatibility with functional layers is critical for ensuring long-term device reliability in automotive and other harsh-environment applications. To evaluate the practical applicability of these substrates in functional devices, silver nanowire (AgNW) electrodes were deposited onto the hybrid films, creating transparent conducting systems. As presented in Fig. 4e, the AgNW-coated hybrid film exhibits a smooth, uniform electrode surface, with scanning electron microscopy confirming consistent nanowire distribution across the substrate. The accompanying Fig. 4f demonstrates excellent optical properties, showing that the 20 µm-thick hybrid substrate alone provides 88% optical transmittance at 550 nm, and the AgNW electrode adds only 4–5% additional loss, resulting in a total transmittance of 83% for the complete system. This high transparency is essential for display applications and represents a significant advantage over opaque metal foils. The mechanical flexibility of the AgNW-coated hybrid substrate was rigorously characterized through bending tests. As shown in Fig. 4g, the relative change in resistance (Δ*R*/*R*_0_) is plotted as a function of bending radius (*R*_c_). The electrode demonstrates negligible resistance change even at extreme bending radii as small as 270 µm, indicating that both the substrate and the nanowire network maintain excellent mechanical integrity under severe deformation. Resistance increases only slightly to 12% when the bending radius drops below 100 µm, corresponding to a bending-induced strain of approximately 10%. At this point, structural damage to the AgNW network becomes appreciable. This performance is significantly superior to that of conventional plastic-substrate-electrode systems, where early failures typically occur at lower bending radii. The cyclic mechanical durability was assessed through a 10 000-cycle bending fatigue test, as presented in Fig. 4h. During repetitive tensile and compressive bending at a radius of 0.5 mm and frequency of 0.5 Hz, the sheet resistance (*R*_s_) of the AgNW electrode increased by only 11% and 5% for tensile and compressive bending, respectively. These minimal resistance changes indicate exceptional reliability and demonstrate that the low-CTE substrate effectively isolates the electrode from large thermomechanical stresses that would typically cause fatigue failure in conventional systems. The initial sheet resistance of approximately 12 Ω sq^−1^ is maintained with remarkable consistency throughout the cyclic test, providing strong evidence that the CNF hybrid substrate provides superior mechanical support compared to standard polymer films.

**Fig. 4:**
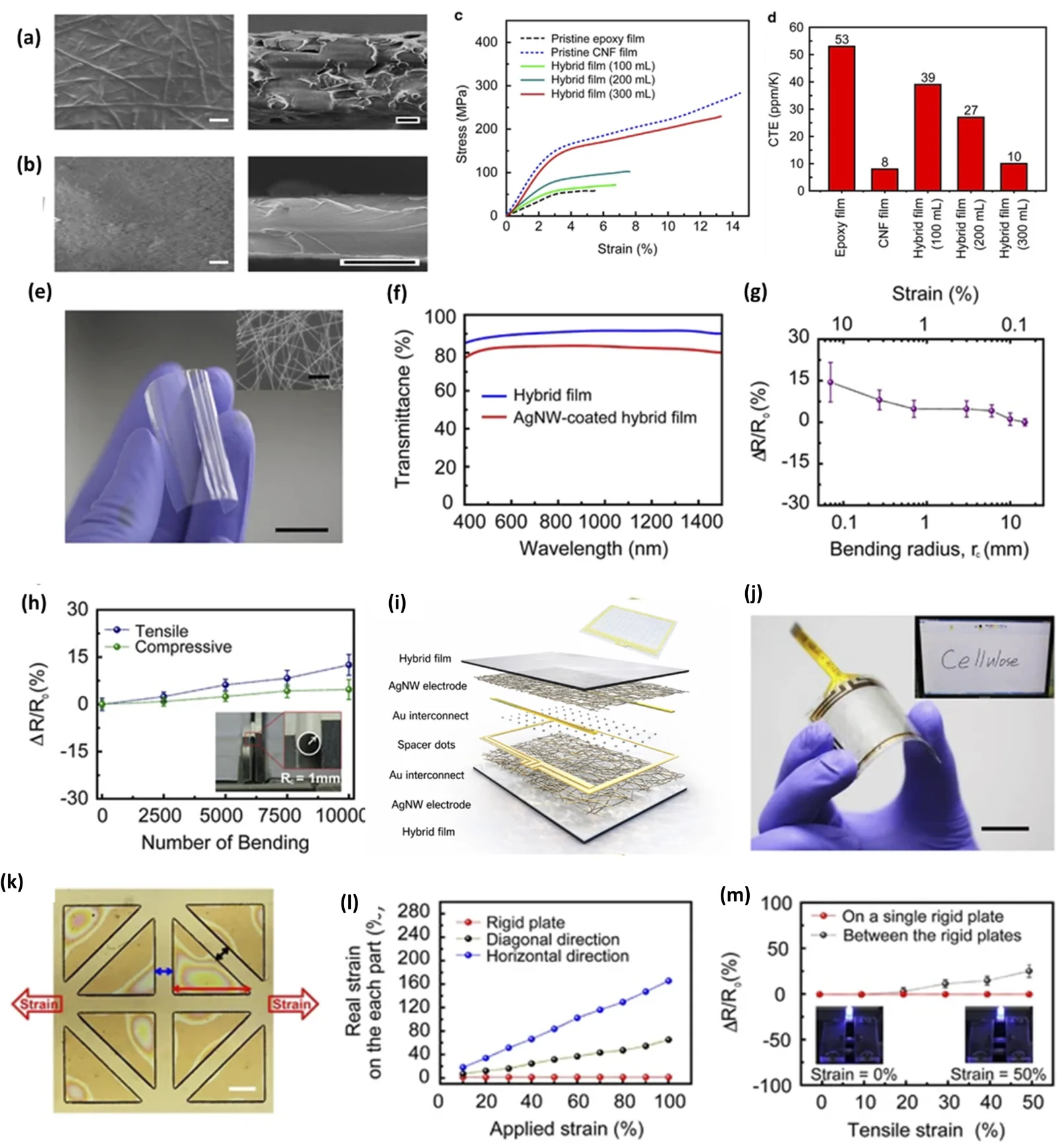
Structural, mechanical, and functional characterization of CNF–epoxy hybrid films and AgNW-coated flexible devices. (a and b) SEM image of the CNF–epoxy hybrid before and after hot pressing. (c) Stress–strain curves showing enhanced mechanical properties of the hybrid compared with those of the pristine epoxy. (d) Thermal expansion of films. (e) AgNW-coated hybrid film; inset: SEM of uniform coating. (f) Optical transmittance spectra. (g) Resistance *vs.* bending radius/strain. (h) Fatigue test over 10 000 cycles. (i and j) Touchscreen schematic and photo; inset/SI Movie S1 shows operation. (k) Stretchable origami substrate (optical micrograph). (l) Strain distribution. (m) Resistance change under strain; inset: LED lit at 50% strain, this figure has been adapted from ref. 102 with permission from nature, copyright 2016.

To demonstrate practical device integration, a four-wire resistive touchscreen panel (TSP) was fabricated using the hybrid films as transparent, flexible substrates for both the upper and lower electrode layers. As illustrated in Fig. 4i, the device architecture incorporates AgNW transparent electrodes, photolithographically patterned polymer dot spacers, and gold interconnects. The schematic reveals a well-engineered structure that balances the flexibility of the CNF hybrid substrate with electrical connectivity. Fig. 4j (photograph) shows the fabricated touchscreen panel, which demonstrates exceptional operational performance. The touchscreen remains fully functional, with smooth touch responsiveness across the entire active area. The critical thermal reliability test was conducted by exposing integrated touchscreen panels fabricated on CNF hybrid films and on conventional PET films to a sustained elevated temperature (100 °C) for 10 days. The CNF hybrid-based TSP operated stably with negligible performance degradation, and no cracking was observed in the gold interconnections. In stark contrast, the identical TSP architecture fabricated on PET films failed; the large thermal expansion of the PET substrate (approximately 60 ppm K^−1^) induced numerous cracks in the gold interconnects, rendering the device inoperable. This dramatic performance difference directly illustrates the importance of CTE matching and validates the CNF hybrid films as a superior substrate material. The minimal mismatch between the CNF hybrid (10 ppm K^−1^) and the metal electrodes (approximately 14 ppm K^−1^) ensures mechanical compatibility and long-term reliability under thermal cycling, which is critical for automotive and outdoor applications. Beyond conventional mechanical flexibility, the CNF hybrid films exhibit unique capabilities for stretchable origami-based electronics through integration of patterned rigid plates with elastomeric polydimethylsiloxane (PDMS) joints. This innovative architecture exploits the photo-patternability of the CNF hybrid films to create strain-engineered substrates that maintain electrical functionality under extreme deformation. As shown in Fig. 4k, an optical micrograph displays the fabricated stretchable origami substrate, featuring isosceles right-angled triangle rigid plates composed of patterned CNF hybrid material, separated by PDMS elastomeric joints with a thickness of 100 µm. This design elegantly separates mechanical functions: the rigid plates remain strain-free during deformation, while the soft PDMS regions absorb the applied mechanical stress.

The strain distribution analysis, presented in Fig. 4l, quantifies the effectiveness of this strain-engineered design. When a tensile strain (*ε*) is applied to the overall origami substrate, the rigid CNF hybrid plates experience negligible strain (*ε*_plate_ ≈ 0), while the elastomeric regions accommodate large effective strains in different directions: *ε*_horizontal_ = 1.62*ε* (horizontal direction across the PDMS joints) and *ε*_diagonal_ = 0.59*ε* (diagonal direction). This uneven strain distribution is precisely engineered by the geometry and placement of the elastomeric joints, allowing the rigid plates which carry the electrical circuits and functional devices to remain nearly strain-free. This is a critical achievement, as most semiconductor devices, dielectric materials, and metal interconnects are brittle and cannot tolerate large strains without failing. The electrical reliability of this stretchable origami architecture is demonstrated in Fig. 4m, which plots the relative changes in sheet resistance (Δ*R*/*R*_0_) of AgNW electrodes coated on the stretchable origami substrate as a function of applied tensile strain. Most remarkably, when an AgNW electrode is deposited on a rigid CNF hybrid plate, its sheet resistance remains nearly constant (*i.e.*, negligible change) even under 50% global tensile strain, confirming that the strain-free nature of the rigid plates effectively isolates the electrode from mechanical deformation.

In contrast, AgNW electrodes deposited on the elastomeric PDMS joints exhibit greater resistance changes (Δ*R*/*R*_0_ = 24% at 50% strain), which remain acceptable for practical electrode applications given the low initial resistance (*R*_0_ = 13 Ω sq^−1^). This demonstration establishes the origami substrate as a viable platform for truly deformable electronics that maintain functional electrical properties under extreme mechanical stress. The practical utility of this stretchable origami architecture is exemplified by the fabrication of a three-dimensional origami structure (a regular dodecahedron) using an AgNW-coated CNF hybrid substrate. The planar origami pattern is folded to create the three-dimensional geometry, with an inorganic light-emitting diode (ILED) integrated within the structure to serve as a functional element that demonstrates mechanical robustness. The ILED illuminates reliably even when the dodecahedron origami structure is deformed, providing compelling visual evidence that CNF hybrid films enable unprecedented form-factor flexibility while maintaining electrical functionality. This capability opens pathways for emerging applications in origami-inspired electronics, wearable devices with complex geometries, and adaptive structures that change shape in response to environmental stimuli.

These achievements position CNF hybrid films as a promising candidate for widespread application in automotive electronics, wearable devices, flexible displays, and other demanding applications where reliability under thermal and mechanical stress is paramount.

## 3. CNF as binder and structural support in conductive composites

### 3.1. CNF in MXene/h-BN composite films

One of the most critical roles of CNF in electronic textiles is serving as a binder material to hold conductive fillers together while providing mechanical reinforcement. Qian *et al.*^103^ demonstrated this application comprehensively by fabricating Ti_3_C_2_T_*x*_/hexagonal boron nitride (h-BN) composite films where CNF acted as the binding matrix. In their innovative approach, the negatively charged Ti_3_C_2_T_*x*_ and CNF were assembled *via* coulombic interactions with positively charged h-BN during fabrication, yielding tightly packed films with distinctive zigzag fracture structures.^103^ Additionally, Qian *et al.*^103^ reported that electrical conductivity increased dramatically from 4.96 S cm^−1^ to 57.67 S cm^−1^ with the incorporation of h-BN conductive nanofillers, demonstrating an order-of-magnitude enhancement that is crucial for efficient charge transport in electronic devices. These results highlight CNF's dual functionality as both a structural binder and an enhancer of electrical and thermal performance in e-textile applications.^103^

### 3.2. Mechanism of CNF binding in composites

The mechanism behind these improvements is the coulombic assembly process, which ensures uniform distribution of conductive fillers throughout the CNF matrix. The electrostatic interactions between oppositely charged components create a stable three-dimensional network that facilitates electron and phonon transport while maintaining mechanical integrity.^103^ This approach represents a sophisticated materials engineering strategy that leverages the inherent properties of cellulose nanofibers to achieve multifunctional performance.

To increase thermal conductivity or EMI shielding properties, adding conductive fillers to organic or inorganic polymers is considered the most efficient approach.^104^ Wang *et al.*^104^ demonstrated that combining conductive fillers in polymer matrices can significantly enhance both electrical and thermal transport properties. The CNF matrix provides a continuous phase that maintains mechanical integrity while allowing for high loadings of functional fillers without compromising processability.

### 3.3. Surface modification strategies

Furthermore, the functional groups of textile substrates and MXenes enable additional surface modification, thereby enhancing the functional properties of MXene-based textronics. For instance, Wang *et al.*^105^ prepared MXene/PPy ink *via* an *in situ* polymerization process before dip-coating poly(ethylene terephthalate) (PET) textiles. Pyrrole solution was first introduced into the MXene suspension, and, due to MXene's acidic properties, long polypyrrole (PPy) polymer chains formed, with the MXene terminal groups. After that, the PET textile pretreated with NaOH was dipped into a stable MXene/PPy ink. Furthermore, a silicone coating was applied to MXene/PPy@PET textiles to create a hydrophobic surface, which solved the oxidation problems of MXene and enhanced wash fastness (Table 2).^105^

**Table 2:** Influence of surface modifiers including CNF on functional properties of MXene-based textronics

Textronic architecture	Surface modifier (S.M.)	Purpose of using S.M.	Property enhancement	Ref.
Ti_3_C_2_T_*x*_/h-BN	**CNF**	Binder of conductive fillers	Electrical conductivity (57.67 S cm^−1^)	103
Spongy Fe_*x*_Co_1−*x*_P/WKF/MXene	Spongy FexCo_1−*x*_P	Increasing the conductive pathway, bind MXene nanosheets	Conductivity ↑, Joule heating (74 °C at 3 V), impact resistance (131.4%)	106
MXene–PPy textiles	Polypyrrole	Protective layer to avoid oxidation	Capacitive performance ↑	107
PPy/MXene decorated PET textiles	Polypyrrole, silicone	Formation of conductive path, stability, prevents oxidation	Water resistance ↑, electrical conductivity ↑, EMI shielding ↑, Joule heating ↑	105
MXene/CNC coated TPU fabric	CNC	Achieving stable homogenous MXene dispersion with good strength	Enhanced sensing properties	108
MXene/CNC coated TPU fabric	PDA	Increasing wettability of TPU NWF	Enhanced adhesion	108
AgNW/MXene/PET textiles	PDA	Enhance bonding strength	EMI shielding ↑, sensing performance ↑	109
AgNW/MXene/PET textiles	AgNW	Forming 3D conductive network	Conductivity ↑	109
AgNW@MXene coated PET textiles	NaOH	Improve surface activity of PET textiles	Antibacterial ↑, flame retardant ↑, EMI-shielding ↑, electrothermal ↑, photothermal ↑	110
AgNW@MXene coated PET textiles	PVP	Enhance the bonding strength between PET and AgNW along with MXene	Enhanced adhesion	110
AgNW@MXene coated PET textiles	AgNW	Enhance conductivity	Electrical conductivity ↑	110

## 4. Fabrication techniques for CNF-based electronic textiles

The fabrication strategy plays a critical role in determining the electrical, mechanical, and functional performance of CNF-enabled MXene textiles. Before discussing individual fabrication techniques, Table 3 provides a comparative overview of representative approaches, enabling direct comparison of their performance characteristics and application potential.

**Table 3:** Comparative summary of fabrication strategies and performance characteristics of cellulose nanofiber (CNF)-enabled MXene textile systems for wearable therapeutic applications

Fabrication category	Representative system	Electrical conductivity	Mechanical properties	Wash durability	Therapeutic/functional application	Advantages	Limitations	Representative study
Fiber	MXene-coated cellulose yarn	>440 S cm^−1^	High flexibility and mechanical integrity	Stable after 45 wash cycles	Conductive fibers and wearable interconnects	Simple fabrication, lightweight, flexible	Limited multifunctionality	Wei *et al.*^111^
Yarn	CNT/polyester wrapping yarn	∼15 kS cm^−1^	Tensile strain ≈ 143%; excellent cyclic stability	Excellent	Motion sensing and physiological monitoring	High conductivity and stretchability	Relatively complex fabrication	Yin *et al.*^112^
Fabric (woven/knitted/nonwoven)	MXene-functionalized textile	High	Woven: high strength; knitted: high elasticity; nonwoven: high porosity	Good	Smart garments, rehabilitation textiles, wound dressings	Comfortable, breathable, scalable	Performance depends on textile architecture	Wang *et al.*;^101^ Li & Tao^43^
Dip coating	Ti_3_C_2_T_*x*_ MXene-coated fabrics	>440 S cm^−1^	Preserves flexibility	Stable after 45 washing cycles	Conductive textiles and Joule heating	Simple, low-cost, scalable	Limited penetration into thick fabrics	Wei *et al.*^111^
Spray coating	MXene-coated textile	High	Flexible coating	Moderate	Wearable conductive textiles	Fast, scalable process	Coating uniformity may vary	Chen *et al.*^44^
Blade coating	MXene composite film	High	Uniform coating thickness	Good	Flexible electronics	Precise thickness control	Mainly applicable to flat substrates	Hong *et al.*^48^
Electrochemical deposition	Conductive polymer/MXene textile	High	Strong coating adhesion	Good	Sensors and electroactive textiles	Precise microstructure control	Complex processing	Zhang *et al.*^49^
Electrospinning	CNF nanofibrous membrane	Moderate	Highly porous nanofiber network	Moderate	Drug delivery and wound healing	High drug loading, controlled release	Lower mechanical robustness	Hashem *et al.*;^63^ Walther *et al.*^46^
Wet spinning	TPU/carbon black conductive fibers	∼210 S m^−1^	Elongation >2400%	Good	Stretchable electronic textiles	Excellent elasticity	Lower conductivity than MXene systems	Jalalah *et al.*^66^
Thermal drawing	Multifunctional smart fibers	Application dependent	High structural integrity	Excellent	Integrated sensing, stimulation, and drug delivery	Highly integrated multifunctional fibers	Requires specialized equipment	Orf *et al.*;^68^ Yan *et al.*^69^
Hybrid CNF/MXene composites	CNF/MXene conductive composite	High	Excellent balance of strength and flexibility	Good	Multifunctional therapeutic textiles	Synergistic electrical and mechanical properties	More complicated fabrication	Dong *et al.*;^113^ Lu *et al.*^114^

As summarized in Table 3, each fabrication strategy offers distinct advantages depending on the targeted therapeutic application. Coating techniques generally provide simple and scalable routes for producing conductive textiles, whereas spinning methods enable the fabrication of mechanically robust and highly flexible fibers. Hybrid CNF–MXene composite systems offer the most balanced combination of electrical conductivity, mechanical integrity, and multifunctionality, making them particularly attractive for next-generation wearable therapeutic platforms. The following sections discuss these fabrication approaches in greater detail.

### 4.1. Coating methods

Coating is one of the most convenient methods for fabricating CNF- and MXene-based conductive textiles. The coating technique mainly involves depositing a thick layer of desired materials on the substrate's surface, thereby significantly improving the substrate's mechanical, electrical, thermal, and chemical properties.^115^ There are numerous coating approaches including dip coating, spray coating, *in situ* polymerization, drop-casting, and spin coating.^115^

#### 4.1.1. Dip coating

The dip-coating technique is one of the simplest processes for loading MXene onto fabric or yarn surfaces. In this process, fabric or yarn substrates are dipped into the MXene solution for several minutes. Hatter *et al.*^116^ reported that the substrate is then dried in air or placed in an oven drier at a certain temperature. This dipping and drying process can be repeated several times to increase the amount of MXene on the surface. This technique can control the thickness of the coating layer.^101^

Wang *et al.*^101^ fabricated MXene-modified core–sheath elastic yarn (CSY). For preparing CSY, polyurethane and polyester filaments were simultaneously fed into a roller, which was then twisted together to form the yarn. The CSYs were ultrasonicated in water to remove wax and impurities. After that, the CSYs were dipped into MXene solution for 10 minutes, followed by drying at 120 °C in an oven for 10 minutes.^101^

Liu *et al.*^117^ used polymeric textiles, poly(ethylene terephthalate) (PET), to load MXene nanosheets by dip coating approaches. Before that, the polymeric textile surface was treated with strong alkali (NaOH) solution at 80 °C for 2 hours. During NaOH treatment, the ester groups of PET textiles are hydrolyzed, yielding oxygen functional groups. Therefore, after dipping alkali-treated PET textiles into MXene dispersion, strong interfacial interaction occurred between the surface functional group of MXene and PET textiles.^117^

Moreover, the dip-coating technique enables large-scale production of MXene-based textronics due to its simple fabrication process. Zou *et al.*^118^ demonstrated that cotton rovings were immersed in MXene dispersion for 30 minutes followed by drying. Then, an electroactive stretchable yarn was fabricated by spinning MXene/cotton rovings and spandex yarn.

#### 4.1.2. *In situ* polymerization

The *in situ* polymerization technique allows polymerization on the fabric surface rather than initiating it separately. The uniform deposition of polymeric materials on the substrate's surface is the main advantage of *in situ* polymerization, ensuring strong bonding between the polymer and the fiber. Modification of MXene-treated fabric by *in situ* polymerization technique involves the following steps: (1) MXene-modified fabric or yarn is dipped into a monomer solution; (2) an oxidant solution is added to the MXene-treated fabric or yarn surface to start the polymerization process.^119^

Yang *et al.*^119^ fabricated PPy/MXene@cotton (PMCF) yarn using this technique. At first, they soaked cotton yarn in a mixture of pyrrole (Py) and MXene solution. To initiate polymerization, 1 wt% C_14_H_7_NaO_5_S and 4 wt% (NH_4_)_2_S_2_O_8_ were used. The fabricated yarn showed excellent mechanical strength of 107.3 MPa and electrical conductivity of 60.8 S m^−1^. The fabrication process demonstrated in Fig. 5a showed the step-by-step integration of conductive polymers with MXene-coated cellulosic yarns.^119^

**Fig. 5:**
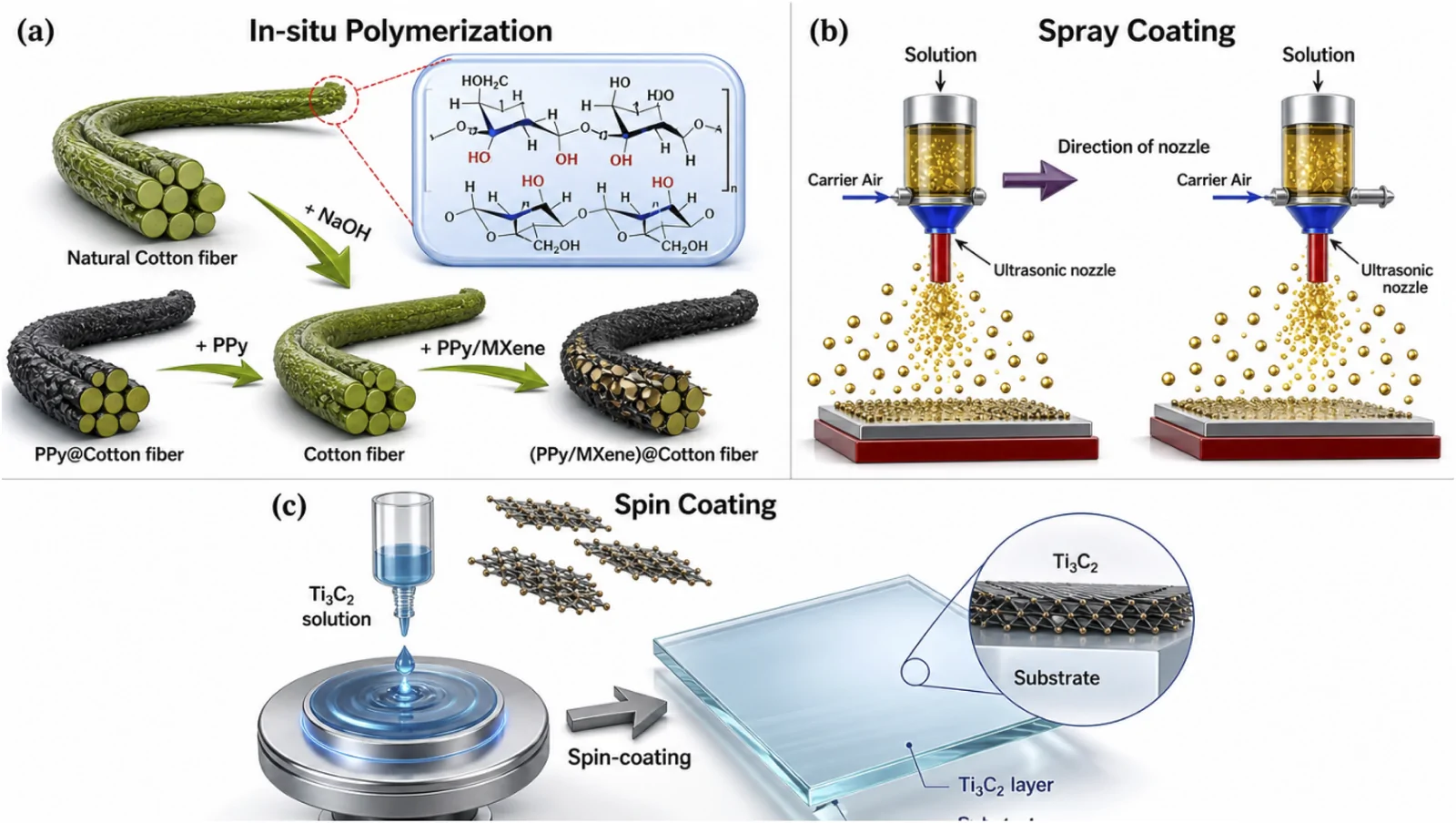
Representative fabrication approaches for conductive material deposition. (a) Schematic illustration of the *in situ* polymerization process of pyrrole monomers for forming conductive coatings, this figure has been reproduced from ref. 119 with permission from Elsevier, copyright 2021. (b) Depiction of the spray-coating method, highlighting the progressive buildup of the deposited layer with increasing deposition time, this figure has been reproduced from ref. 123 with permission from Elsevier, copyright 2015. (c) Spin-coating process employed for the uniform deposition of MXene nanomaterials, this figure has been reproduced from ref. 128 with permission from Wiley, copyright 2023.

Li *et al.*^120^ used Py monomer for *in situ* polymerization. Before polymerization, they modified the fabric with positively charged polyethyleneimine (PEI) and negatively charged Ti_3_C_2_T_*x*_ MXene nanosheets. Therefore, electrostatic interaction occurred between PEI and MXene nanosheets. After that, the MXene/PEI-modified fabric was soaked in a pyrrole/*p*-toluenesulfonic acid (PTSA) dispersion, followed by the addition of an oxidant solution containing FeCl_3_·6H_2_O (0.87 g) to initiate polymerization.

In another study, Yin *et al.*^121^ demonstrated that aniline monomer was used for polymerization to modify a carbon fiber (CF) fabric. Before that, the CF was pretreated with HNO_3_ solution. Therefore, a large number of oxygen-containing polar groups and positive charges were generated, which favored MXene loading onto the fabric surface. Then, MXene/CF was dipped into aniline monomer solution. To deposit the polyaniline (PANI) layer on MXene/CF fabric, APS oxidant was added.

Although *in situ* polymerization allows uniform deposition of polymeric materials on a bulk scale, it doesn't allow control over the thickness of electroactive materials on the substrate. In addition, because the monomer solution must be deposited on the substrate before polymerization, polymer aggregation may occur after polymerization. Furthermore, unreacted monomer may remain due to a lack of control over the polymerization process. For these reasons, the concentration of monomer and oxidant for the polymerization process needs to be selected very carefully.^119,121^

#### 4.1.3. Spray coating

Spray coating of MXene nanomaterials involves spraying MXene solution inks onto the textile substrate through a nozzle under pressure. Aziz and Ismail^122^ reported that after spraying, the textile substrates are dried, subjecting the evaporation of liquid materials and allowing the dried MXene nanosheets to adhere to the substrate. Fig. 5b shows a schematic illustration of the spray coating process at two different times. Zabihi *et al.*^123^ demonstrated that this technique is widely used in art and industries.

Large-scale production can be achieved using the spray-coating process because it requires less time to deposit the active materials on the substrate. Bishop *et al.*^124^ reported that moreover, thin deposition of active materials on the substrate is achieved within a very short time. In addition, the thickness of the coating layer can be controlled by adjusting several parameters: the distance between substrate and nozzle, air pressure, and solution concentration. Zhang *et al.*^125^ demonstrated that, in general, the MXene concentration for spray coating is kept between 0.1 mg mL^−1^ and 10 mg mL^−1^.

#### 4.1.4. Spin coating

In spin coating, the desired solution is dropped onto the substrate, which is then rotated for a specified time to deposit the coating material, after which the solvent evaporates. Mendhe^126^ reported that the main advantage of spin coating is its ability to deposit coated materials homogeneously on the substrate. However, there will be more solution wastage (almost 80–90%). Nandy and Chae^127^ quantified this material loss.

Fig. 5c shows the process of spin coating of MXene nanomaterials. Ahn *et al.*^128^ illustrated the step-by-step process. The applied shear force and the coating duration can control the thickness of the coated layer. Hall *et al.*^129^ demonstrated the relationship between spin speed and film thickness. For instance, Purbayanto *et al.*^130^ prepared rGO/Ti_3_CNT_*x*_ film *via* spin-coating techniques. The obtained film exhibited a sheet resistance of approximately 19 kΩ sq^−1^.

In another study, He *et al.*^131^ demonstrated that Cu–Ni alloy-coated nylon textile was used as the substrate for spin-coating of MXene/PDMS mixture. Although the spin-coating technique ensures a homogeneous distribution of the materials, the thickness and homogeneity of the coating layer may be disrupted by changing the spinning speed. Tian *et al.*^132^ reported this limitation. Another major disadvantage of spin-coating is the size of the substrate, which hinders scalable production. Moreover, increasing the substrate size makes it difficult to achieve high-speed spinning performance. Yilbas *et al.*^133^ discussed these constraints. Furthermore, during solvent vaporization, high temperatures may weaken the fabric's strength, thereby deteriorating its performance.

### 4.2. Electrospinning

The electrospinning technique is widely recognized as one of the most efficient methods for fabricating nanofibers in nonwoven form. Bhardwaj and Kundu^33^ provided a comprehensive review of electrospinning principles. Electrospinning involves three main basic components: (1) a high voltage supplier, (2) a spinneret, and (3) a collecting screen. In the electrospinning process, when a high voltage is applied, the polymer solution exits the spinneret and is collected on the collecting screen. Before reaching the collecting screen, the polymer solution is solidified.

Park^134^ and Li *et al.*^135^ described the solidification mechanisms. On the collecting screen, the nanofiber is collected as a nonwoven web with a high surface area-to-volume ratio. During electrospinning, the spinneret is exposed to the applied electric field, which creates a surface charge on the liquid. Huang *et al.*^136^ reported that increasing the applied voltage forms a conical shape of the liquid at the tip of the spinneret, known as the Taylor cone, and further increasing the voltage helps overcome surface tension. Taylor^137^ first described this phenomenon. Finally, the charged liquid is ejected from the Taylor cone and deposited on the collector.

Fig. 6a shows a schematic illustration of the horizontal setup of the electrospinning apparatus. Bhardwaj and Kundu^33^ illustrated the complete setup. When high electric voltage is applied during electrospinning, loading high-conductive liquids may result in short circuits, which can deteriorate performance. Usman *et al.*^115^ discussed this limitation. To address this shortcoming, carbonizing the nanofiber can be an effective approach.

**Fig. 6:**
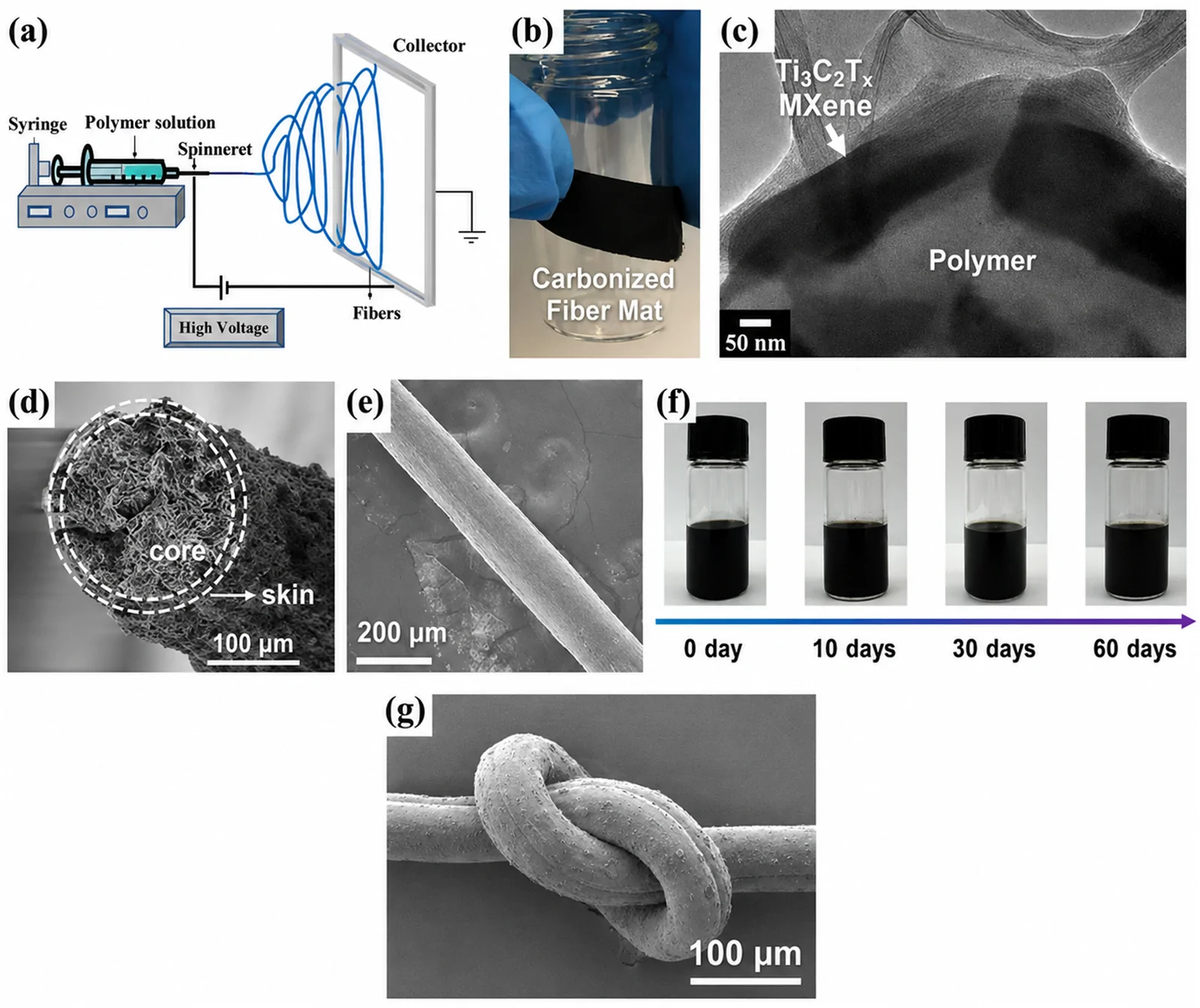
Electrospinning and wet-spinning strategies for MXene-based fiber fabrication. (a) Schematic representation of a horizontal electrospinning setup. (b) Carbonized MXene/PAN electrospun nanofiber wrapped around a glass vial, illustrating its outstanding mechanical flexibility. (c) Electrospinning-enabled homogeneous dispersion of MXene nanosheets within a polymer matrix. (d) Core–shell morphology of a wet-spun Kevlar/MXene composite fiber. (e) Surface morphology showing the smooth and uniform exterior of the wet-spun Kevlar/MXene fiber. (f) Photograph of a stable MXene/silk precursor solution used for wet spinning. (g) Knitted textile constructed from MXene/silk wet-spun fibers, demonstrating excellent flexibility, this figure has been reproduced from ref. 151 with permission from The Royal Society of Chemistry, copyright 2024.

Levitt *et al.*^138^ demonstrated that MXene/PAN electrospun nanofibers were carbonized to increase conductivity and improve supercapacitive performance. Before the carbonization process, the MXene/DMF solution was centrifuged to collect the MXene sediment, which was then added to the PAN/DMF solution, and the mixture was stirred overnight to prepare the spinning solution. The spinning solution was poured into the spinneret, and then the voltage was applied. Finally, the nanofiber mat was collected and carbonized to prepare MXene/PAN carbonized composites. The obtained nanofiber mat demonstrated greater flexibility, as shown in Fig. 6b. In addition, Fig. 6c exhibits the homogenous interconnection of MXenes along the fiber axis.^138^

In another study, Fu *et al.*^139^ found that Ti_3_C_2_T_*x*_-MXene/poly(vinylidene fluoride-*co*-trifluoroethylene) (P(VDF-TrFE)) nanofibers were prepared *via* an electrospinning process. For this, the MXene/DMF solution and the P(VDF-TrFE)/DMF/acetone solution were transferred into a syringe and electrospun at an applied voltage of 15 kV. Then, the nanofiber was obtained on aluminum foil. To increase the conductivity, the nanofiber film was further dipped into MXene solution.

MXene nanomaterials have wide surface functional groups and for this reason, MXene can be dispersed in a wide variety of polar solvents, such as chitosan,^140^ poly(acrylic acid) (PAA),^141^ poly(ethylene oxide) (PEO),^141,142^ poly(vinyl alcohol) (PVA),^141^ and alginate/PEO.^141^ Mayerberger *et al.*^140,141^ extensively studied MXene dispersions in these biopolymers. However, during electrospinning, one should be very careful, as a high applied voltage can cause an electric shock. In addition, several parameters, such as solution viscosity, MXene loading, applied voltage, distance between the spinneret and collector, temperatures, and many others, should be carefully selected.^140,141^

### 4.3. Wet spinning

Wet spinning has gained enormous attention nowadays for the fabrication of flexible yarn that can be woven or knitted into garments for the production of smart wearable devices. Rohani Shirvan *et al.*^143^ provided a comprehensive review of wet spinning for advanced materials. To fabricate yarn by wet spinning, four steps are followed: (1) dissolution of polymer materials, (2) extrusion of the polymer solution from the spinneret, (3) coagulation, and (4) collection of the yarn.

A polymer solution is typically extruded from the spinneret into the coagulation bath, where it begins to solidify. Qin *et al.*^144^ described the coagulation mechanisms. After that, the yarn is washed and collected on a rotating beam. In the wet spinning process, several parameters, such as the rheological properties of the polymer solution, the solvents in the coagulation bath, the spinneret diameter, the winding speed, and the processing temperature, play important roles.^143^

Using this technique, it is possible to produce continuous yarn, which is nowadays in high demand for wearable electronic devices. In wet spinning MXene materials, the main challenge is controlling the weak interlayer interactions between MXene nanosheets. Introducing additional materials as binders may solve this challenge. However, the mass ratio of active materials to binders should be carefully selected so that neither component adversely affects the desired properties of the fabricated yarn.^145^

#### 4.3.1. Wet spinning of MXene/polymer blends

Zhang *et al.*^146^ used PEDOT:PSS as a conductive binder with Ti_3_C_2_T_*x*_ to produce a wet-spun fiber. The wet-spun MXene/PEDOT:PSS fiber showed a high conductivity of 1489 S cm^−1^ with 70 wt% of MXene loading. In the wet-spinning process, the MXene/PEDOT:PSS spinning solution was passed through a coagulation bath containing sulfuric acid (H_2_SO_4_), then washed with a water/ethanol mixture, and finally dried. Introducing MXene disrupted π–π stacking in PEDOT and enabled well-aligned MXene nanosheets to be incorporated into PEDOT:PSS. In addition, the intense MXene peak at both 50 wt% and 70 wt% loading confirmed the presence of MXene flakes in the PEDOT:PSS matrix.

Cheng and Wu^147^ used Kevlar fiber as the reinforcing agent in wet-spinning with Ti_3_C_2_T_*x*_-MXene. Kevlar fiber was exfoliated with methanesulfonic acid (MSA) and trifluoroacetic acid (TFA) to make Kevlar acid solution. Then MXene was homogeneously dispersed in the Kevlar acid solution to prepare the Kevlar/MXene (KM) spinning dope. The KM solution was extruded through a spinneret into an acetic acid bath, then dried in air and collected on a bobbin. Fig. 6d and e show the core–skin structure and homogenous surface of the KM fiber, respectively, proving the successful preparation of Kevlar/MXene wet-spun fiber.^147^

In a similar study, Wang *et al.*^148^ demonstrated that aramid nanofiber (ANF)/DMSO solution and Ti_3_C_2_T_*x*_-MXene/DMSO solution were used for spinning dopes. The ANF/DMSO and MXene/DMSO were placed separately in two needles. Then the spinning dope was extruded into the coagulation bath at a constant rate of 0.03 mL min^−1^. The extruded fibers solidified due to interaction with the coagulant solvent, DMSO/H_2_O/CH_3_COOH. The resultant fiber showed a high mechanical strength of 130 MPa. In MXene/ANF wet-spun fiber, aramid acted as the high-strength frame around the MXene layer due to the connection of ANF with the coagulant bath. Furthermore, hydrogen bonding between the MXene hydroxyl group and the amide groups of aramid strengthened the MXene/ANF fiber.^148^

Besides synthetic polymeric fibers, naturally abundant polymers, such as silk, cellulose, alginate, chitosan, and others, are widely used with MXene materials because good interfacial interactions between MXene and natural polymers can be achieved *via* hydrogen bonding. Furthermore, MXene/natural polymer composites ensure biodegradability, biocompatibility, and a reduced carbon footprint. In addition, synthetic polymer fibers cannot ensure proper air permeability. Therefore, the use of natural polymer with MXene nanomaterials is increased day by day to fabricate soft, flexible, wearable electronic devices.^149^

Yue *et al.*^149^ used silk fibroin as the biotemplate with MXene nanosheets to fabricate a degradable, stretchable wet-spun fiber. In the wet-spinning process, the MXene dispersion and silk solution were mixed, with no agglomeration or precipitation observed even after 60 days (Fig. 6f). The spinning solution was transferred into a coagulation bath containing (NH_4_)_2_SO_4_, and the fibers were rolled at 10 rpm. Furthermore, the fibers were passed through a CaCl_2_ bath to increase their toughness. Fig. 6g shows the knitted structure of the produced fiber, demonstrating flexibility, stretchability, and softness, which is favorable for the fabrication of wearable electronic devices.^149^

Furthermore, Usman *et al.*^150^ demonstrated that crystalline nanocellulose (CNC) was used with Ti_3_C_2_T_*x*_-MXene nanomaterials to induce liquid-crystalline behavior for the fabrication of wet-spun fibers. The liquid-crystalline properties are essential for achieving appropriate alignment and processability during wet spinning, thereby ensuring that the resulting fibers have optimal mechanical and electrical properties.^150^

#### 4.3.2. Wet spinning of MXene/carbonaceous materials

Seyedin *et al.*^145^ used liquid crystal graphene oxide with Ti_3_C_2_T_*x*_-MXene nanosheets to fabricate yarn. They prepared the spinning solution by sequential centrifugation to increase MXene loading while preserving LC behavior. In addition, they used acetic acid as the coagulant solvent to produce high-strength fiber. Fig. 7a shows the knitted MXene/GO fiber and knitted structure, respectively, proving the flexibility of the MXene/GO wet-spun fiber.

**Fig. 7:**
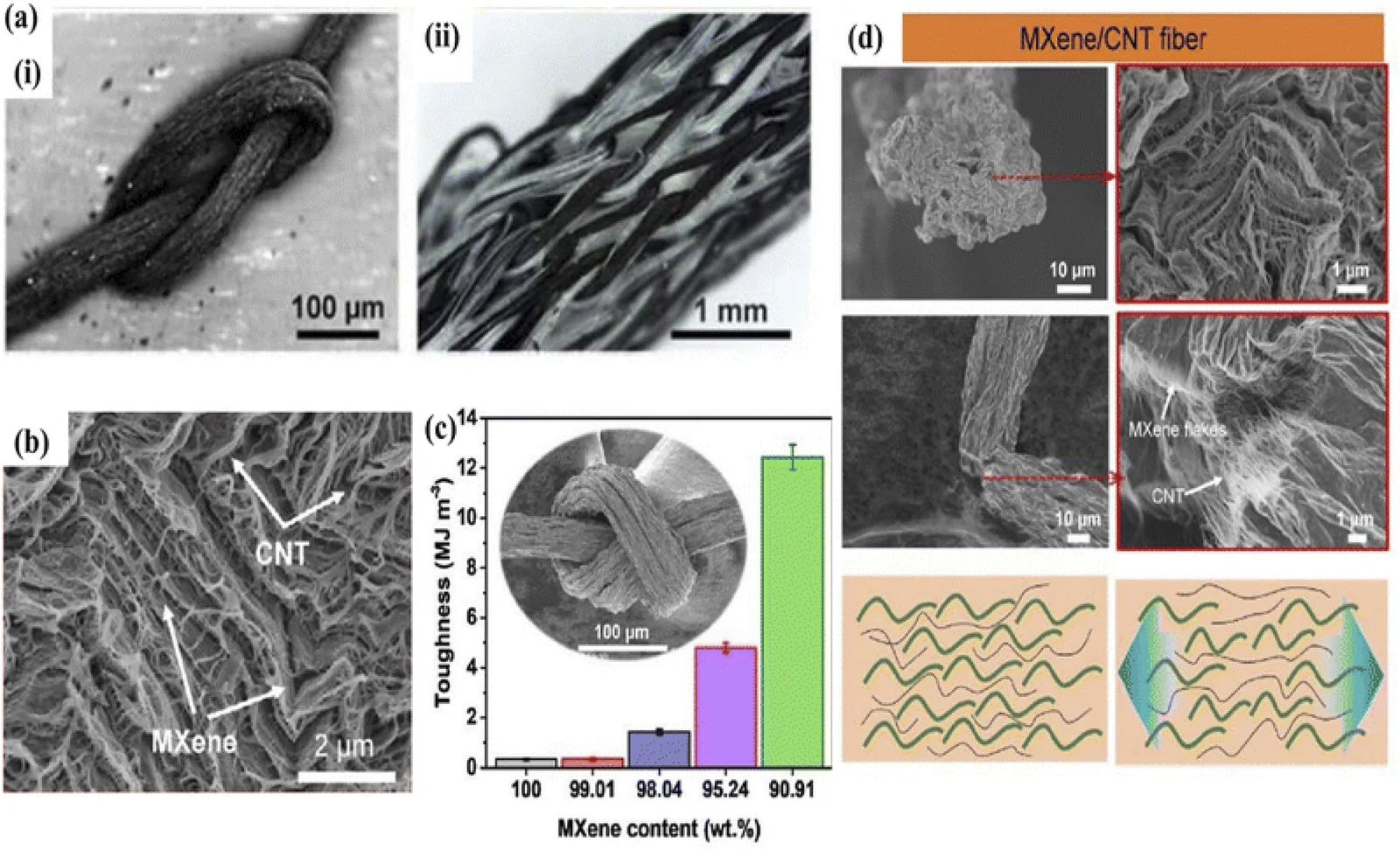
Structural and mechanical characteristics of MXene-based composite fibers. (a) Knitted textile fabricated from highly flexible wet-spun MXene/GO fibers, this figure has been adapted from ref. 145 with permission from The Royal Society of Chemistry, copyright 2017. (b) Scanning electron microscopy (SEM) image of a MXene/CNT composite nanofiber showing a uniform morphology without noticeable aggregation. (c) Mechanical toughness performance of the wet-spun MXene/CNT fiber. (d) Schematic depiction of the fiber under tensile loading, where elongation of CNT networks contributes to enhanced mechanical robustness, (b–d) have been adapted from ref. 152 with permission from Elsevier, copyright 2022.

Zhao *et al.*^152^ fabricated Ti_3_C_2_T_*x*_-MXene/CNT wet-spun fiber with 1 wt% CNT loading. To prepare a homogeneous spinning dope, CNT was first added to sodium taurodeoxycholate (STDOC) to form a CNT dispersion, imparting a negative charge on CNT. Thus, repulsion between the negatively charged CNT and MXene nanomaterials resulted in homogeneous spinning solutions. Fig. 7b shows the cross-sectional image of MXene/CNT nanofiber with no agglomeration. In addition, Fig. 7c shows that mechanical toughness increased with increasing CNT content due to strong interconnection between CNT and MXene, which was much higher than that of pure MXene fiber.^152^

Furthermore, under stress, as shown in Fig. 7d, the CNT stretched along the fiber axis, thereby enhancing the mechanical toughness of the wet-spun fiber.^152^ For the practical application of smart textiles, the conductive yarn must be highly stretchable, as textile-based wearable devices undergo various bending conditions in daily use. For this purpose, Wu *et al.*^153^ used thermoplastic polyurethane (TPU) as the polymer framework and MXene/CNT as the conductive networks. To prepare the wet-spinning solution, a MXene/DMF suspension was prepared by adding CNT, followed by stirring to form a MXene/CNT dispersion. Then TPU was added to the MXene/CNT mixture, and the solution was stirred at 70 °C to prepare the final spinning dope. CNTs/MXene–TPU spinning dispersion was injected into a coagulation bath containing water to solidify the fiber. In the coagulation bath, DMF diffused rapidly, leading to the formation of MXene/CNT–TPU fibers.

Moreover, Zheng *et al.*^154^ used sodium hyaluronate (HA) as a dispersant to ensure uniform dispersion of MXene and CNT in the spinning solution. HA acts as a protective layer to mitigate restacking issues in MXene and CNT. In addition, a coagulation bath containing 75% ethanol and CaCl_2_ was used to solidify the fiber.

#### 4.3.3. Wet spinning of pure MXene-based fiber

Wet spinning of pure MXene fibers is challenging due to processing issues, including weak interlayer bonding, oxidation, and others. To address this issue, Eom *et al.*^155^ developed a new method to fabricate wet-spun MXene fibers without the use of binders or additives. Ti_3_C_2_T_*x*_-MXene was prepared by etching the Al layer from Ti_3_AlC_2_ precursors. Centrifugation was carried out repeatedly to concentrate the MXene nanosheets (25 mg mL^−1^), resulting in a liquid crystalline phase. Moreover, the shear stress gradually increased after a certain time, indicating the uniform orientation of MXene nanosheets. The as-prepared LC MXene dispersion was extruded into a coagulation bath containing NH_4_^+^ ions, followed by washing with water to obtain a continuous, pure MXene fiber. The prepared fiber showed a highly compact structure. Furthermore, the pure MXene fiber exhibited an ultrahigh electrical conductivity of 7713 S cm^−1^, enabling it to transmit signals from mobile phones.

In another study, Zhou *et al.*^156^ demonstrated that to fabricate MXene fibers, MXene nanosheets were mixed with glutaraldehyde solution to induce liquid-crystalline properties. A high-concentration (30 mg mL^−1^) spinning solution exhibited a viscosity of 4.1 kPa s, suggesting good spinning properties. This highly concentrated MXene spinning solution was extruded into a coagulation bath containing ammonium chloride, ammonium hydroxide solution, and deionized water. The fiber was then transferred into a PVA solution, followed by washing with deionized water to obtain the MXene (MGP) fibers. The obtained MGP fibers were further transferred into a polymer tube for thermal drawing. Thus, the obtained fiber showed a high mechanical strength of 585.5 MPa and a high electrical conductivity of about 8802.4 S cm^−1^.

### 4.4. Biscrolling technique

The biscrolling technique involves the guest-host concept to fabricate hybrid yarn by using twist-based spinning. Wu *et al.*^157^ described the fundamental principles of this technique. Nowadays, this unique technique is used to fabricate MXene-based yarn. In the fabrication of MXene-based yarn, MXenes serve as guest materials, while carbon nanotubes (CNTs) serve as the host material. This technique may allow high electrochemical performance, electrical conductivity, and mechanical properties.

Yu *et al.*^158^ fabricated an MXene/CNT fiber by the biscrolling technique. The Ti_3_C_2_T_*x*_-MXene nanomaterials were dispersed in a water/DMF solution and then drop-cast onto a CNT scaffold using a pipette. The MXene/CNT thin film was then rolled into a helical fiber. This process enabled a higher loading of Ti_3_C_2_T_*x*_-MXene (95%) as the guest material into the CNT scaffold's corridor. In such a guest-host hybrid structure, CNTs provided mechanical robustness and higher electrical conductivity. As a result, the fiber material can be used in different forms, such as knitting it into a glove or winding it around a glass rod. The fabricated yarn exhibited excellent volumetric capacitance (22 F cm^−3^). This proves that the biscrolling technique is useful for fabricating MXene-based yarn for the production of smart textiles.^158^

Wang *et al.*^159^ also followed the biscrolling strategy to prepare Ti_3_C_2_T_*x*_-MXene/CNT yarn, referred to as BMX yarn. In this technique, the guest materials, MXene, can be confined within the corridor of the host materials, CNT, creating a porous structure. The high pore volume and hydrophilic nature of MXene nanosheets facilitate rapid electrolyte ion transport, thereby improving electrochemical performance.^159^ The biscrolling technique is very useful for producing flexible, high-strength conductive yarn that may facilitate the fabrication of smart wearable garments for multiple applications. However, the main disadvantage of this technique is that it is limited to CNT as the host material. Meena *et al.*^160^ discussed this limitation (Table 4).

**Table 4:** Advantages and disadvantages of different fabrication approaches for MXene and CNF-based textronics

Fabrication method	Advantages	Disadvantages	Ref.
Coating	• Simple and fast fabrication process	• Uneven thickness of electroactive materials	15
	• Lower cost	• Careful selection of concentration required	
	• Large-scale production	• Limited to surface coverage	
	• CNF can be easily incorporated as binder		
Biscrolling	• No need for a binder	• Host material limited to CNT	161
	• Accessible to high loading of guest materials	• Limited length of yarn	
	• Highly porous yarn can be prepared	• Difficult to incorporate CNF	
Wet spinning	• Continuous length of fiber	• Careful selection of coagulant solvent required	162
	• Scalable production	• Rheology of spinning solutions critical	
	• CNF can serve as binder and reinforcement	• Complex spinning setup	
Electrospinning	• High surface area to volume ratio	• Difficult to fabricate with highly conductive materials	26
	• Various composite nanofibers can be produced	• Tends to agglomerate	
	• CNF compatible with process	• High voltage requirements	

## 5. Factors affecting functional properties of CNF-based e-textiles

### 5.1. Fiber type effects

Fiber type plays an important role because it influences the final properties of MXene and CNF-based textronics. Cotton, polyester, Kevlar, wool, silk, *etc.*, differ in their properties. For instance, Kevlar, aramid, or basalt fibers are stronger than cotton or wool fibers. Therefore, such high-strength fibers should be used where higher strength is needed, for example, body armor for soldiers.^163^

Qian *et al.*^163^ demonstrated that MXene-modified basalt fiber-reinforced polymer matrix exhibited ultrahigh flexural strength of 530.1 MPa and flexural modulus of 26.7 GPa. In addition, this lower mechanical strength may arise from a lower degree of polymer linkage in the polyester fiber than in the aramid fiber. In addition, if MXene and CNF-based textronics are to be used as flexible wearable sensors or thermal devices, fiber elasticity is required. In this regard, Lycra fiber (spandex yarn) can be used because it has high elasticity. Sinha *et al.*^164^ characterized the elastic properties of spandex fibers.

For instance, Zou *et al.*^118^ demonstrated that MXene-modified cotton/spandex yarn (MCSY) showed excellent recoverability with up to 100% stretching. In addition, at 20% strain, the stretch recoverability after 20, 80, and 100 repeated tensile cycles was almost identical. Fig. 8a shows the stretching behavior of the yarn. Such MXene-coated yarn showed a wide sensing performance when attached to the human wrist and back neck, as shown in Fig. 8b.^118^

**Fig. 8:**
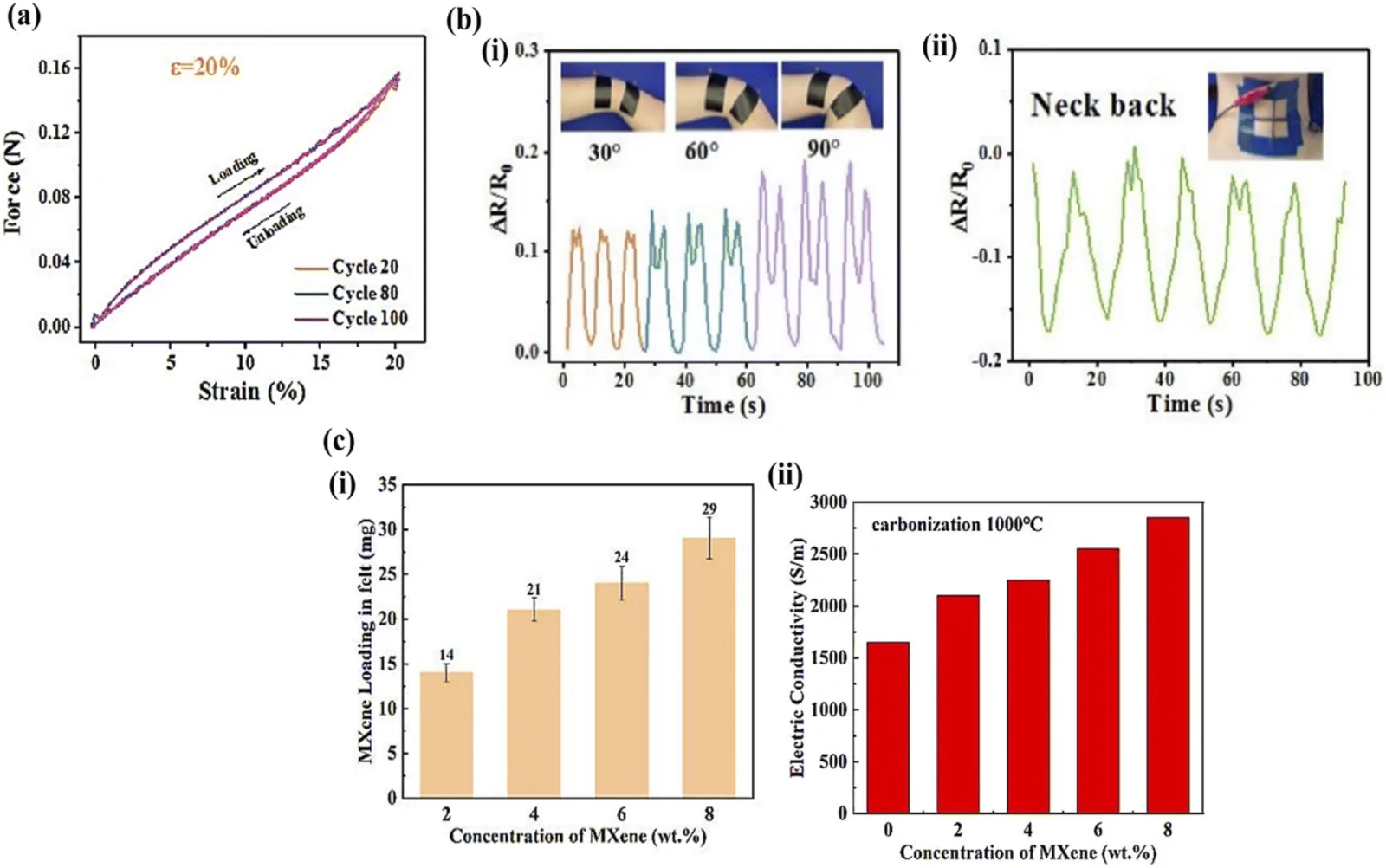
Mechanical and sensing performance of MXene-based composite yarns. (a) Representative force–strain profile of the MXene/cotton/spandex composite yarn, this figure has been adapted from ref. 118 with permission from American Chemical Society, copyright 2023. (b) Demonstration of wearable sensing capabilities of the MXene/cotton/spandex yarn when positioned on the (i) wrist and (ii) posterior neck region, this figure has been adapted from ref. 118 with permission from the American Chemical Society, copyright 2023. (c) Correlation between MXene concentration and resulting MXene content as well as electrical conductivity, indicating enhanced conductivity with higher MXene loading, this figure has been reproduced from ref. 166 with permission from nature, copyright 2023.

### 5.2. Fabric structure effects

Not only fiber use but also the fabric structure plays a vital role in enhancing the functional properties of MXene- and CNF-based textronics. In particular, air permeability, a key comfort parameter, and conductivity largely depend on the fabric structure because it exhibits gaps between adjacent yarns. Smaller gaps ensure a more compact, uniform coating of MXene nanosheets, whereas larger pores allow more MXene molecules to pass, thereby directly affecting conductivity.^165^

Moreover, production costs may increase because more MXene nanomaterial is needed to deposit on a fabric with larger pores than on a fabric with smaller pores. The fabric's pore size depends on its structure. For instance, woven fabrics have a more compact structure than knitted fabrics, which may allow the uniform deposition of MXene nanomaterials. In addition, it is possible to produce various types of woven fabrics (*e.g.*, plain weave, satin weave, twill weave, diamond weave, *etc.*) and knitted fabrics (*e.g.*, single jersey and double jersey, plated patterns, *etc.*), which also have different pore sizes depending on their design.^165^

Huang *et al.*^165^ investigated the load capacity of MXene nanomaterials on three different weave structures: plain, twill, and satin cotton fabrics to understand the photothermal and sensing performances. After dip-coating the fabrics into 5 mg per mL MXene solutions, three different levels of MXene content were achieved: 0.175 mg cm^−2^, 0.228 mg cm^−2^, and 0.297 mg cm^−2^ for plain weave, twill weave, and satin weave, respectively. Therefore, the satin fabric showed the highest conductivity among the satin weaves. The highest MXene content in satin fabric can be attributed to its fewer interweaving points, higher density, and thicker structure compared to the plain and twill weaves. As a result, MXene-decorated satin fabric showed excellent photothermal properties, including an ultrahigh temperature of 251.7 °C and a wide pressure range of 20 kPa, making it a pressure sensor.^165^

Besides the traditional two-dimensional (2D) fabric structures (*e.g.*, knitted and woven), three-dimensional (3D) fabric structures highly influence the functional properties of MXene and CNF-based textronics as, unlike the 2D fabric structure, the 3D structured fabric possesses a higher surface area, increasing the MXene loading percentage on the fabric surface. Fan *et al.*^166^ prepared 3D denim fiber from waste jeans, which was immersed in MXene solution to form a conductive layer. As the 3D structure has an additional fiber direction (*Z*) in addition to *X* and *Y*, the 3D denim fiber provides a larger contact area, increasing MXene loading and electrical conductivity, as shown in Fig. 8c. Therefore, the fabricated conductive 3D denim fiber showed a superior specific capacitance of 1748.5 mF cm^−2^ and a remarkable stability of more than 94% after 15 000 charge/discharge cycles.^166^

### 5.3. MXene size effects

MXene size has a substantial effect on the functional properties of MXene and CNF-based textronics. The etching methods of Ti_3_C_2_T_*x*_-MXene endow the MXene surface with numerous functional groups and affect the MXene flake size. Thakur *et al.*^167^ comprehensively reviewed the effects of synthesis conditions on MXene properties. For instance, reducing the amount of HF solution by using a combination of HCl, water, and HF in a 6 : 3 : 1 volumetric ratio during the preparation of Ti_3_C_2_ MXene resulted in larger MXene nanomaterials. Driscoll *et al.*^168^ demonstrated this approach.

Moreover, the combination of LiF and HCl, rather than HF, during the etching of MAX precursors resulted in larger MXene flakes. Ghidiu *et al.*^169^ developed this milder etching method. In addition to etching methods, post-treatment also affected MXene size. For instance, Maleski *et al.*^170^ investigated the effect of sonication on MXene lateral sizes, which alters electrical conductivity. He has been seen that the conductivity of MXene nanomaterials largely depends on MXene sizes, which can affect the functional properties of MXene and CNF-based textronics. He *et al.*^171^ explored the electrochemical performance of Ti_3_C_2_T_*x*_-MXene/rGO fiber based on different sizes of MXenes. They found that the MXene/rGO fiber prepared from large-size MXenes showed higher conductivity than the fiber prepared from medium-size MXenes. Due to this superior conductivity, large-size and medium-size MXene caused better electrochemical performance than small-size MXenes, as shown in Fig. 9a.^171^

**Fig. 9:**
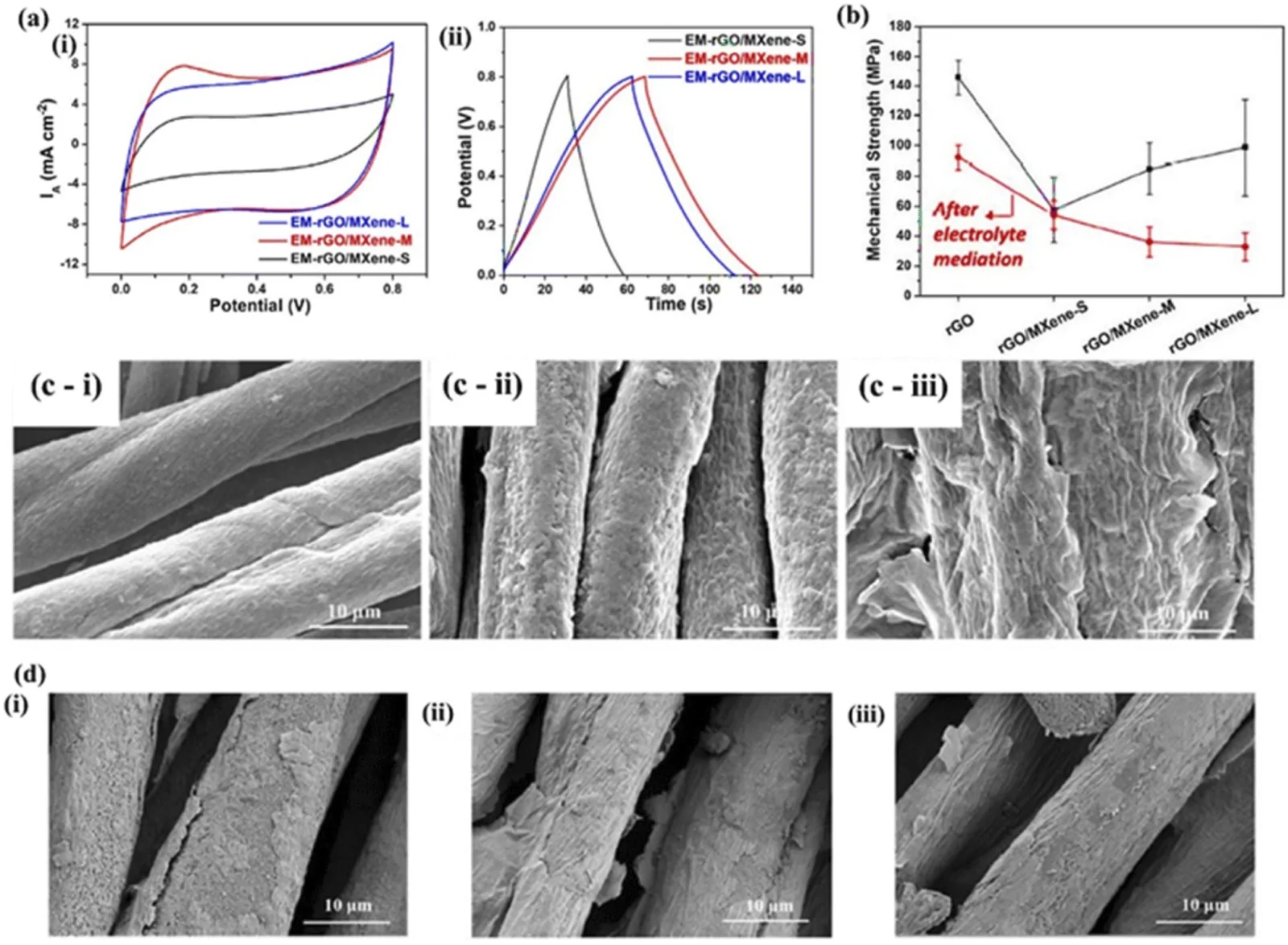
Electrochemical performance and structural characteristics of MXene-based fibers and fabrics. (a) Electrochemical behavior of electrolyte-treated rGO/MXene composite fibers incorporating MXene flakes of varying lateral sizes (large, medium, and small), shown through (i) cyclic voltammetry and (ii) galvanostatic charge–discharge profiles, this figure has been adapted from ref. 171 with permission from the American Chemical Society, copyright 2020. (b) Comparison of the mechanical strength of pristine rGO fibers and rGO/MXene composite fibers containing small-, medium-, and large-sized MXene sheets, evaluated before and after electrolyte treatment, this figure has been adapted from ref. 171 with permission from American Chemical Society, copyright 2020. (c) Photographs of MXene-functionalized fabrics prepared using (i) small-sized MXene, (ii) a mixed small/large MXene system, and (iii) large-sized MXene sheets, this figure has been adapted from ref. 173 with permission from Elsevier, copyright 2022. (d) Scanning electron microscopy (SEM) images of the corresponding MXene-coated fabrics after washing, illustrating the morphological stability of (i) small-sized MXene, (ii) mixed small/large MXene, and (iii) large-sized MXene modifications, this figure has been adapted from ref. 173 with permission from Elsevier, copyright 2022.

However, from Fig. 9b, it is evident that the mechanical strength declined for large- and medium-size MXene nanomaterials compared with small-size MXenes after electrolyte mediation, which was ascribed to a reduction in the interfacial interaction between MXene and rGO.^171^ In supercapacitor applications, large-size MXene nanomaterials may provide sufficient electrolyte-ion transport pathways. Therefore, MXene nanoflakes with more open space are suitable for wearable supercapacitor applications. However, for wearable electronic devices, mechanical strength is crucial because they undergo significant deformation when worn on the human body. Therefore, optimizing MXene size without compromising strength is essential.^171^

Before considering this issue, the following effects of MXene nanosheets should be carefully observed: (1) MXene nanoflakes with smaller sizes show lower conductivity; however, it introduces defects on the MXene surface, causing the enhancement of electroactive sites that aid in increasing the capacitance;^172^ (2) large MXene size enables the electrolyte ion transportation, although it may reduce the strength due to lack of interfacial interaction.^171^ Peng *et al.*^172^ discussed the relationship between MXene size and electrochemical performance. Considering the conductivity, wash fastness, and mechanical strength, Zhang *et al.*^173^ formulated that the combination of small/large MXene coating on cotton fabric shows better electromagnetic shielding properties than the large and small MXene alone. They concluded that small MXene particles covered the fiber surface due to their tiny size, but did not cover the fabric's pores. However, the large MXene covered the pore space, allowing a more conductive path, as shown in Fig. 9c.^173^ After washing, the large MXene tended to fall off, while the small MXene remained on the fabric surface. Therefore, regarding the combination of small/large MXenes, small MXenes covered the fiber or yarn surface, while large MXenes bridged between the yarns, increasing the binding force. Fig. 9d shows the coating of three different sizes of MXene on the fabric surface after washing.^173^

## 6. CNF-enhanced strain and pressure sensors

Cellulose nanofiber-modified thermoplastic polyurethane (TPU) nonwoven fabrics have shown remarkable potential as wearable strain and pressure sensors with exceptional performance. Li *et al.*^108^ fabricated MXene/cellulose nanocrystal-modified TPU non-woven fabric, where a special pre-stretching treatment was applied to create micro-crack structures within the conductive layer. This innovative approach leverages the unique properties of CNF to enhance sensor sensitivity through engineered structural modifications.^108^

These micro-crack structures enhance sensor sensitivity by disrupting electron transportation pathways under applied strain or pressure. When external pressure is applied, the conductive pathways are altered, altering electrical resistance that can be measured and correlated with the magnitude of the applied force.^108^

The gauge factor, which defines pressure sensitivity, can be calculated by the rule GF = (Δ*R*/*R*_0_)/*ε*, where *ε* is the applied tensile strain, Δ*R* = *R* − *R*_0_, *R* is the resistance after applying pressure, and *R*_0_ is the initial resistance. Ma *et al.*^14^ explained this fundamental relationship for strain sensors. When external pressure is applied, the conductive path is disrupted, thereby enhancing sensitivity. Creating microcrack structures within the MXene-modified fabric may enhance sensitivity, as crack surfaces hinder electron transport.^108^

The incorporation of cellulose nanocrystals in this system serves multiple purposes. First, CNF provides structural stability to the TPU substrate, ensuring that the micro-cracks remain controlled and reproducible. Second, the CNF acts as a stable platform for MXene deposition, facilitating the uniform distribution of the conductive material. Third, the hydrogen bonding between CNF and MXene enhances interfacial adhesion, preventing delamination during repeated mechanical deformation.^108^ This approach demonstrates how CNF can be strategically incorporated into e-textile architectures to achieve high-performance sensing capabilities essential for human motion detection and health-monitoring applications.^108^

Ma *et al.*^174^ developed a wearable, hydrophobic, and multifunctional MXene-decorated air-laid paper, followed by surface modification with poly(dimethylsiloxane) (PDMS). The developed MXene-decorated wearable device was used to fabricate pressure sensors, which showed a high sensitivity of 5.78 kPa and a rapid response time (30–40 ms). The air-laid paper substrate, composed primarily of cellulosic fibers, provides an excellent platform for MXene deposition while maintaining breathability and flexibility.

Fig. 10 illustrates the design, sensing performance, and multifunctional application of a flexible strain sensor based on a three-dimensional (3D) hollow MXene sphere/AgNW multidimensional nanocomposite, highlighting how rational nanomaterial integration can overcome the intrinsic limitations of single conductive fillers. Fig. 10 emphasizes that combining MXene and AgNWs not only enhances strain-sensing performance but also opens the way for integrated systems with additional functionalities, such as thermal management, which are increasingly important for next-generation wearable electronics.

**Fig. 10:**
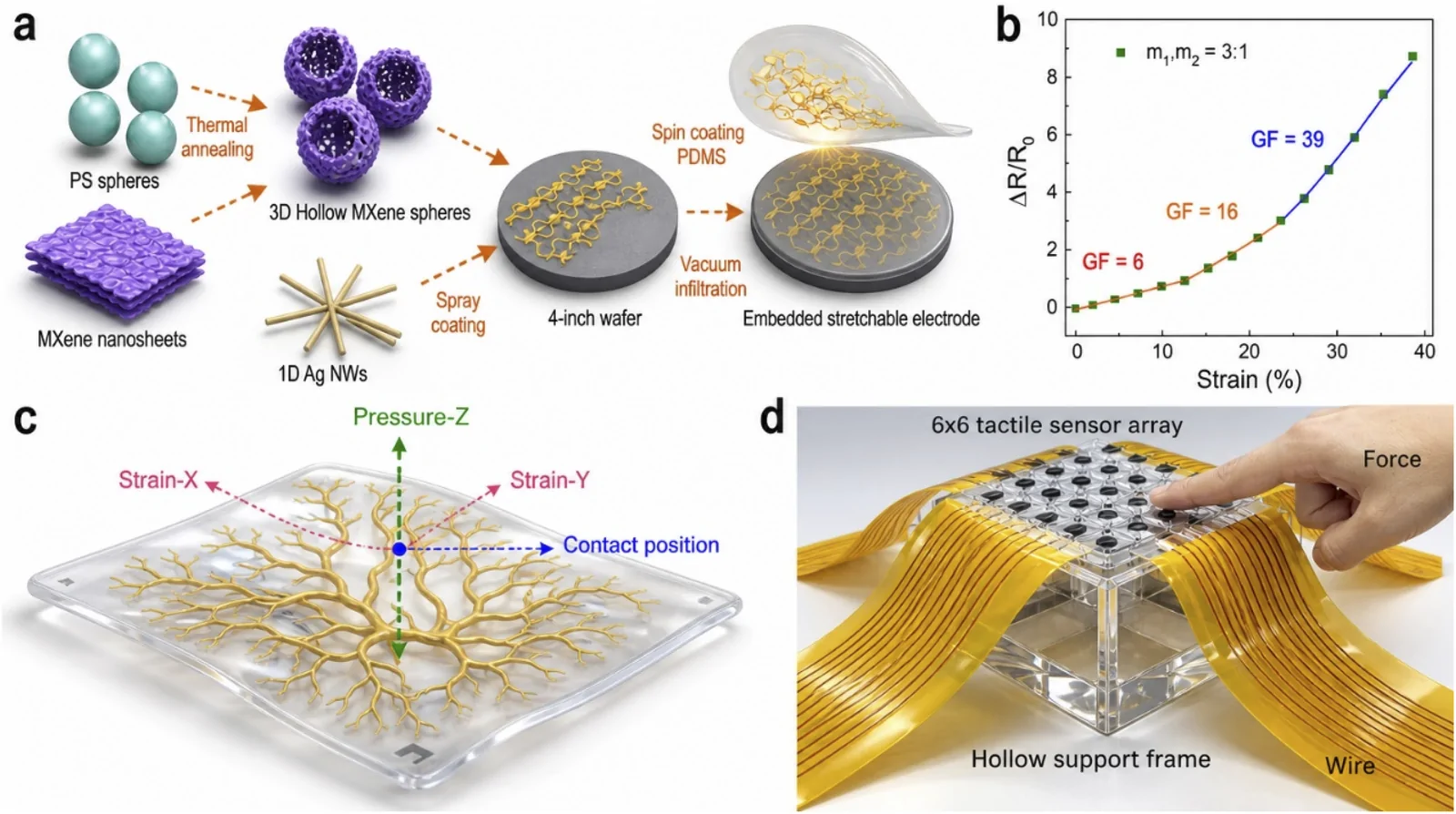
Fabrication, sensing mechanism, and application of a stretchable MXene-based tactile sensor array. (a) Schematic illustration of the fabrication process for embedded stretchable electrodes, including thermal annealing of PS spheres to form 3D hollow MXene structures, integration with MXene nanosheets and Ag nanowires (Ag NWs), followed by spray coating, vacuum infiltration, and PDMS spin coating on a 4-inch wafer. (b) Relative resistance change (Δ*R*/*R*_0_) as a function of applied strain, showing progressive gauge factors (GF = 6, 16, and 39) at increasing strain levels. (c) Working principle of multimodal sensing, enabling simultaneous detection of normal pressure (*Z*-axis), in-plane strain (*X*- and *Y*-axes), and contact position. (d) Photograph of a 6 × 6 tactile sensor array mounted on a hollow support frame, demonstrating force detection capability with integrated wiring, this figure has been reproduced from ref. 175 with permission from the American Chemical Society, copyright 2021.

As shown in Fig. 10a, polystyrene (PS) spheres are first employed as sacrificial templates to fabricate 3D hollow MXene spheres through thermal annealing. These hollow MXene architectures are subsequently hybridized with one-dimensional Ag nanowires (AgNWs) to form a multiscale conductive network. The composite is deposited onto a wafer *via* spray coating and then embedded into a PDMS matrix through spin coating and vacuum infiltration, resulting in an embedded stretchable electrode. In this structure, the hollow MXene spheres serve as localized electron-transport nodes and deformation-tolerant fillers, while the AgNWs form long-range percolation pathways. This synergistic configuration effectively combines high electrical conductivity with mechanical compliance, ensuring stable electrical performance under large deformation.

The electromechanical performance of the resulting strain sensor is presented in Fig. 10b, where the relative resistance change (Δ*R*/*R*_0_) is plotted as a function of applied strain. The sensor exhibits distinct sensitivity regimes, with the gauge factor (GF) increasing with increasing strain. Notably, a high GF of 39 is achieved in the strain range of 25–40%, indicating excellent sensitivity to large deformations. This enhanced sensitivity is attributed to progressive disconnection and reconstruction of the AgNW conductive pathways and the deformation-induced contact variation among the hollow MXene spheres. In addition to high sensitivity, the sensor demonstrates fast response and recovery times of 0.62 s and 0.61 s, respectively, as well as outstanding durability, maintaining stable performance over 2000 loading–unloading cycles at 20% strain. These results confirm the robustness of the multidimensional conductive network under repeated mechanical stress.

Beyond strain sensing, Fig. 10c conceptually illustrates the multiplexed detection capability achieved by integrating the strain sensor with a capacitive pressure sensor based on a T-ZnOw/PDMS dielectric layer. This hybrid sensing configuration enables simultaneous detection of in-plane strains in the *X* and *Y* directions and out-of-plane pressure in the *Z* direction. By analyzing the combined resistive and capacitive signals, the system can determine not only the magnitude of the applied force but also its direction and contact position. Such multidimensional sensing is essential for advanced human–machine interfaces, electronic skin, and intelligent wearable systems that require spatially resolved mechanical perception.

The practical implementation of this multiplexed sensing concept is shown in Fig. 10d, which presents an image of a 6 × 6 tactile sensor array mounted on a hollow support frame. The flexible array accommodates external deformation while maintaining reliable electrical connections *via* embedded wiring. When an external force is applied, localized signal changes across the array enable precise mapping of pressure distribution and contact location. This demonstration highlights the feasibility of the 3D hollow MXene-sphere/AgNW nanocomposite-based strain sensor as a core component for multifunctional wearable platforms.

## 7. Applications of CNF and MXene-based textronics

### 7.1. Wearable supercapacitors

Nowadays, to tackle high energy consumption, the demand for alternative energy sources has been escalating. Xu *et al.*^176^ and Qin *et al.*^177^ discussed the growing need for flexible energy storage. Accordingly, flexible wearable supercapacitors are gaining popularity in everyday life because they exhibit higher power density, shorter charge–discharge times, and longer lifespans than conventional batteries and capacitors.^176,177^

In addition, the usability of flexible wearable supercapacitors can be ensured by analyzing electrochemical performance, such as power density, energy density, capacitance (areal or gravimetric), and electrochemical impedance spectroscopy, under bending, folding, or torsion conditions. In terms of wearability, textile-based materials such as fiber, yarn, or fabric have been considered most suitable due to their lightweight nature, high bending capacity, and superior comfort. Chen *et al.*^31^ provided a comprehensive review of textile-based energy devices.

Furthermore, there are enormous opportunities to surface-modify textile materials to deposit electroactive materials, making them suitable for wearable supercapacitor applications. Among various conductive materials, MXene has been recently used extensively for the fabrication of textile-based wearable supercapacitor devices. Zhang and Nicolosi^29^ and Tang *et al.*^178^ reviewed MXene applications in energy storage. There are various ways to load MXene onto the textile's surface, such as coating, spraying, wet spinning, and more. After modifying the textile's surface with MXene, the electrochemical performance of textile-based supercapacitor devices has been significantly enhanced.^29,178^

Shao *et al.*^142^ developed a new method of nanofiber-coated yarn *via* electrospinning. They electrospun a MXene–polymer composite nanofiber onto a polyester yarn to produce MXene nanofiber-coated yarn electrodes for use as wearable supercapacitor electrodes. The prepared all-solid-state supercapacitor with yarn electrodes showed an areal capacitance of 18.39 mF cm^−2^ at 5 mV s^−1^. In addition, the supercapacitor device showed 98.2% capacitance retention after 6000 cycles, demonstrating exceptional long-term stability suitable for wearable applications.^142^

In another study, Levitt *et al.*^179^ reported the use of a combined electrospinning and wet-spinning technique to produce MXene/nylon nanoyarn for use as an energy storage device. Such yarn electrodes exhibited a high specific capacitance of 440 F cm^−3^ at 5 mV s^−1^ in LiClO_4_ electrolyte solution.

Energy harvesting technologies represent a critical frontier in advancing wearable therapeutic systems from laboratory prototypes to practical, continuous-use medical devices. The integration of energy-harvesting mechanisms directly into textile architectures eliminates the reliance on bulky external power sources, rigid batteries, or tethered electrical connections, which fundamentally compromise the comfort, flexibility, and wearability essential for long-term therapeutic applications.^180–182^ By converting ambient mechanical, thermal, or electromagnetic energy into usable electrical power, textile-integrated energy harvesting systems enable therapeutic devices to operate autonomously and continuously, supporting self-sustaining therapeutic interventions in real-world clinical and home-care environments.^183–185^

Fig. 11a illustrates a self-powered electrotactile textile haptic (SPETH) glove designed for neurosensory rehabilitation, which represents a paradigm shift in wearable therapeutic technology by enabling closed-loop feedback between user intention, physical motion, and sensory stimulation without any external power source.^186^ The system operates on principles of contact electrification and capacitive energy storage, creating a fully self-contained therapeutic platform that delivers adaptive sensory feedback in response to user actions.

**Fig. 11:**
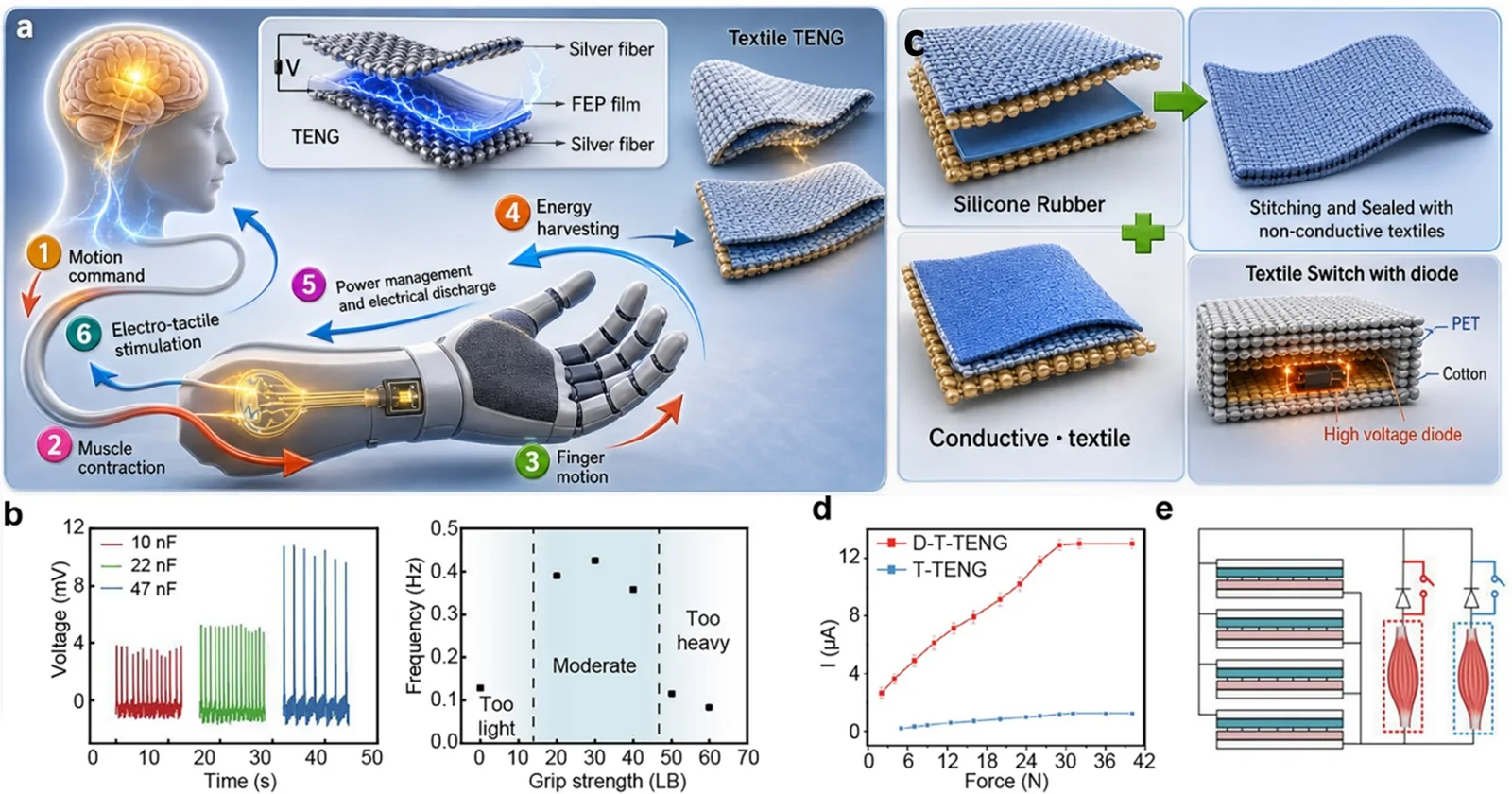
Self-powered energy-harvesting textiles for therapeutic stimulation. (a) Electrotactile rehabilitation glove operating in a closed-loop mode, converting hand grasping into electrical tactile feedback. Inset: structure of the textile triboelectric nanogenerator (TENG). (b) (Left) Tunable stimulation intensity with corresponding EMG signals *via* capacitor selection. (Right) Correlation between stimulation frequency and grip force. (c) Diode-assisted textile TENG with an integrated switch for on-demand muscle stimulation; schematics show the TENG design (top) and the diode-based textile switch (bottom). (d) Peak current output comparison indicating a 26-fold enhancement for the D-T-TENG. (e) Illustration of rat muscle contraction triggered by D-T-TENG-driven electrical stimulation, this figure has been reproduced from ref. 28 with permission from nature, copyright 2025.

The SPETH glove integrates five essential functional components into a seamlessly wearable garment architecture: (1) sensory input sensors that detect grasping motions and grip strength, (2) a triboelectric energy harvesting mechanism that converts mechanical contact into electrical charge, (3) energy storage in the form of capacitors that accumulate and stabilize harvested energy, (4) control circuitry that modulates stimulation parameters based on detected motion, and (5) electrotactile stimulation electrodes that deliver haptic feedback directly to the user's skin through hydrogel interfaces.^186^ This integrated architecture enables a closed-loop system in which user motion directly powers therapeutic feedback, without requiring any external energy input.

The core energy-harvesting mechanism in the SPETH glove relies on contact electrification between specially selected material pairs that maximize triboelectric charge generation during grasping. When the user performs grasping or squeezing motions, fluorinated ethylene propylene (FEP) films, selected for their exceptional triboelectric properties and high electronegativity, make contact with embroidered silver electrodes integrated throughout the glove structure.^186^ During each contact cycle, this material pairing generates approximately 400 nanocoulombs (nC) of electrical charge through triboelectric nanogenerator (TENG) mechanisms. This charge quantity, while seemingly modest at the individual cycle level, accumulates effectively in integrated capacitors to reach useful voltages for therapeutic applications.^186^

The triboelectric effect operates through the transfer of electrons between dissimilar materials during contact and separation. The FEP material, being highly electronegative, readily accepts electrons from the silver electrodes, creating an electrical potential difference that drives current through external circuits when the materials are separated. This process repeats with each grasping motion, creating a continuous and user-responsive energy source that scales with activity level more vigorous or frequent grasping automatically generates more electrical charge.^186^ This activity-dependent power generation provides natural and intuitive control, as the intensity of therapeutic stimulation directly correlates with the vigor of the user's therapeutic exercises.

The electrical charge generated through contact electrification is accumulated in integrated capacitors, where charge storage reaches voltages approaching 250 volts under typical operational conditions.^186^ The capacitor-based energy storage approach offers several critical advantages over battery-based systems for this application. Capacitors can charge and discharge extremely rapidly, enabling the generation of high-current pulses necessary for effective electrotactile stimulation from relatively modest individual grasping motions. Capacitors inherently avoid the wear-out issues associated with chemical batteries, since the charge storage mechanism relies on electrostatic separation of charges rather than chemical oxidation–reduction reactions that gradually degrade over repeated cycles.^184^ Furthermore, capacitor-based storage scales seamlessly with motion intensity. Vigorous grasping generates more charge, which accumulates to higher voltages, enabling proportionally stronger stimulation. In contrast, gentle or infrequent grasping results in moderate stimulation levels.

The fundamental innovation in Fig. 11a is the establishment of a true closed-loop feedback system where multiple physiological and mechanical variables interact continuously to provide personalized, adaptive therapeutic stimulation. The system closes the feedback loop through seven integrated stages: ① the user's intent to perform a therapeutic grasping exercise, ② initiation of the grasping motion, ③ contact between the FEP and silver electrode materials generating triboelectric charge, ④ accumulation of electrical charge in capacitors, ⑤ control circuitry determining appropriate stimulation parameters based on measured grip strength, ⑥ delivery of electrotactile pulses through hydrogel electrodes, and ⑦ sensory feedback to the user's fingertips and palm, which reinforces the motor intention and supports sensory-motor reintegration.^186^

This closed-loop architecture is particularly valuable for neurosensory rehabilitation following stroke, peripheral nerve injury, or other neurological conditions that disrupt normal sensory-motor integration. In typical stroke recovery, patients experience profound loss of sensory feedback from affected limbs, which severely impairs motor control even when some muscle function remains. By providing electrotactile feedback that is directly responsive to the patient's motor effort, the SPETH glove helps restore the critical sensory component of the sensory-motor loop, facilitating neuroplasticity and functional recovery.^187,188^ The fact that feedback intensity automatically scales with motion vigor creates natural biofeedback that encourages patients to perform therapeutic exercises with greater effort and consistency. Fig. 11b demonstrates the remarkable capability of the SPETH glove to deliver controllable stimulation intensity through a clever approach: the user (or clinician) can select different capacitor values to be charged by the same triboelectric energy source.^186^ Since the same amount of triboelectric charge (approximately 400 nC per grasp) distributes across different capacitor sizes, the resulting voltage scales inversely with capacitance larger capacitors accumulate to lower voltages. In comparison, smaller capacitors reach higher voltages from the same harvested charge. By offering multiple capacitor options (for example, 1 µF, 10 µF, 100 µF, or 1000 µF), clinicians can provide graded stimulation intensities appropriate to different patient conditions or rehabilitation stages.

The left panel of Fig. 11b plots stimulation intensity (measured as electromyography response amplitude) against capacitor selection, showing a clear dose–response relationship.^186^ Smaller capacitors produce greater voltage accumulation and, correspondingly, stronger electrotactile stimulation, resulting in larger EMG responses in the activated muscles. Larger capacitors produce lower voltages and weaker stimulation, generating more modest muscle responses. This relationship enables clinicians to start patients with stronger stimulation intensities to ensure clear sensory perception and robust muscle activation, then progressively transition to larger capacitors as patients improve, thereby creating a natural progression of therapeutic difficulty aligned with recovery.

Importantly, this intensity modulation occurs without any external control electronics, wireless communication, or battery management systems. The only change required is the mechanical substitution of one capacitor for another a simple, robust, and failure-proof approach that eliminates complicated control electronics and associated power requirements. This simplicity is particularly important in clinical settings and home environments where device reliability and ease of use are paramount.

The right panel of Fig. 11b illustrates an even more sophisticated control feature: the system automatically modulates stimulation frequency based on the user's measured grip strength.^186^ As the user increases grip force during a therapeutic grasping exercise, the contact area and contact pressure between the FEP and silver electrodes increase, generating more triboelectric charge per contact cycle and charging the capacitor to a useful voltage more frequently. This automatic frequency modulation creates a natural biofeedback mechanism where patients who perform stronger or more vigorous grasping exercises receive more frequent stimulation pulses, providing immediate reward and reinforcement for increased effort.

This frequency-based biofeedback is physiologically sensible and therapeutically valuable. During stroke recovery or rehabilitation following hand injury, many patients develop learned non-use; they avoid using affected limbs because of weakness or poor sensory feedback. By providing immediate, effort-responsive sensory feedback, the SPETH glove directly counteracts learned non-use by creating positive reinforcement for increased motor effort. The patient perceives that stronger grasping generates stronger or more frequent feedback, encouraging them to challenge themselves with greater force and more repetitions. This user-responsive stimulation is far more engaging and motivating than fixed-intensity stimulation that does not respond to the patient's actions.

The relationship between grip strength and stimulation frequency shown in the right panel demonstrates that this coupling occurs naturally without explicit control algorithms it emerges directly from the physics of the triboelectric harvesting mechanism. Stronger grasping creates more charge, which charges the capacitor more rapidly, enabling more frequent discharge pulses. This elegant example of passive control demonstrates how properly designed mechanical and material systems can automatically provide sophisticated therapeutic functions without the need for active electronic controllers.

While the SPETH glove shown in Fig. 11a demonstrates remarkable capabilities for self-powered electrotactile feedback, conventional triboelectric nanogenerators (TENGs) suffer from limitations that limit their applicability to certain therapeutic scenarios. Specifically, conventional textile TENGs generate electrical pulses passively in response to mechanical deformation, providing no direct control over discharge timing or pulse intensity. For many therapeutic applications, particularly neuromuscular stimulation for muscle strengthening or functional electrical stimulation for movement restoration, clinicians need precise control over stimulation timing to achieve specific therapeutic objectives. Additionally, discharging a capacitor through skin electrodes without control circuitry can result in unpredictable current waveforms that may be uncomfortable or ineffective for therapy.

To address these fundamental limitations, researchers developed the diode-enhanced textile TENG (D-T-TENG) system illustrated in Fig. 11c, which combines the advantages of self-powered energy generation with clinician-controlled delivery of therapeutic stimulation.^189^ The D-T-TENG integrates three key innovations: (1) a thin textile-based TENG (T-TENG) for energy harvesting, (2) high-voltage diodes that enable directional current flow and prevent back-discharge, and (3) textile-integrated switches that allow on-demand triggering of stimulation delivery.

The core energy-harvesting element, the textile TENG (T-TENG), consists of a copper-coated polyester woven fabric with a thickness of approximately 1.2 millimeters.^189^ This thin profile is critical for practical wearable applications, as thicker textile TENGs would be bulky, stiff, and uncomfortable to wear. The copper coating provides excellent electrical conductivity while maintaining the mechanical properties of the underlying polyester fabric. When the textile undergoes mechanical deformation, such as from body motion, muscle contraction, or manual actuation, the copper-coated layers periodically approach and separate, generating triboelectric charge.

High-voltage diodes are integrated into the textile structure adjacent to the T-TENG, strategically placed to control the direction of current flow.^189^ Diodes allow current to flow in one direction (from the T-TENG to the capacitor and stimulation electrodes) while blocking reverse flow (from the capacitor back to the T-TENG). This unidirectional control is essential for two reasons: first, it prevents self-discharge of the stored capacitor energy back through the T-TENG, which would waste harvested energy; second, it enables the use of textile-based switches to control discharge timing without complex control electronics.

The textile switch represents an elegant innovation that brings clinician-controlled stimulation timing to a fully textile-integrated system. Rather than using conventional mechanical switches with moving parts that would be uncomfortable and unreliable in wearable textiles, the textile switch is constructed from textile materials. It integrates high-voltage diodes in a configuration that allows controlled current flow to stimulation electrodes.^189^ When the switch is “closed” (activated), the diodes allow capacitor discharge to flow through the stimulation electrodes. When the switch is “open” (deactivated), the diodes block current flow, preventing unwanted stimulation.

This switching mechanism enables precise temporal control of stimulation delivery. Clinicians can trigger stimulation pulses at specific times during therapeutic exercises or rehabilitation protocols, ensuring stimulation is delivered at optimal movement phases or in synchrony with the patient's voluntary effort. For example, during functional electrical stimulation for stroke recovery, clinicians can ensure that stimulation is delivered to flexor muscles during the swing phase of gait and to extensor muscles during the stance phase, creating coordinated movement patterns that promote motor learning and functional recovery.

Fig. 11d compares the peak current output of the diode-enhanced D-T-TENG system with the conventional T-TENG without diode enhancement, revealing a dramatic 26-fold increase in delivered current.^189^ The conventional T-TENG without diode control produces approximately 0.5 microamperes (µA) of peak current, while the D-T-TENG achieves approximately 13 µA of peak current. This substantial performance enhancement occurs because the diodes prevent back-discharge and enable charge accumulation across multiple harvesting cycles before controlled release.

In the conventional T-TENG, each mechanical deformation cycle generates a brief pulse of current that immediately flows through any available low-resistance path. If external loads (such as the high impedance of skin tissue) present significant resistance, much of the generated charge dissipates internally, resulting in little productive output. Furthermore, without directional control, any energy stored in capacitors can leak back through the T-TENG during the next mechanical cycle, wasting previously harvested energy.

The diode-enhanced approach concentrates the charge generated across multiple mechanical cycles into organized, high-current pulses delivered through the stimulation electrodes. The capacitor accumulates charge from many deformation cycles, then when the switch activates, the entire accumulated charge discharges through the stimulation electrodes in a high-current pulse. This concentrated delivery is far more effective for achieving therapeutic objectives such as muscle contraction, sensory perception, or tissue stimulation, where high instantaneous current is often more effective than low-level continuous current.

Fig. 11e demonstrates the practical therapeutic efficacy of the D-T-TENG system through *in vivo* experiments on anesthetized rats, a standard animal model for validating functional electrical stimulation devices.^189^ The dorsal surface of a rat hindlimb is exposed, revealing the major muscles including the tibialis anterior (TA), which performs foot dorsiflexion (upward foot movement), and the gastrocnemius (GM), which performs plantarflexion (downward foot movement). Stimulation electrodes from the D-T-TENG are placed on the motor points of these muscles the locations where stimulation most efficiently activates muscle contraction.

By selectively stimulating TA or GM muscles through the D-T-TENG system, researchers demonstrate precise control over limb movement direction. When TA is stimulated, the rat's foot visibly dorsiflexes (moves upward). When GM is stimulated, the foot plantarflexes (moves downward). Most importantly, these directional responses occur reliably and repeatedly, demonstrating that the D-T-TENG system delivers consistent, controllable stimulation capable of producing reproducible, directionally specific movement.^189^

The ability to produce directionally specific, controllable movements in an *in vivo* animal model demonstrates the therapeutic potential of the D-T-TENG for functional electrical stimulation (FES) applications in humans. In clinical practice, FES is used to restore voluntary movement in patients with paralysis from spinal cord injury, stroke, or other neurological conditions. Successful FES requires precise timing (stimulating appropriate muscle groups at appropriate phases of movement) and precise intensity (delivering sufficient current to activate muscles without causing discomfort or tissue damage). The D-T-TENG meets both requirements: the textile switch provides temporal control, and the accumulated charge-based approach provides intensity control through capacitor selection.

The *in vivo* validation demonstrating directional control is particularly significant because it shows the system works not only on isolated tissue but also in a living organism with complex feedback mechanisms, variable physiological responses, and realistic impedance conditions. The fact that directional movement control is achieved and reliable suggests that clinical FES applications may be feasible, restoring the ability to flex or extend limbs voluntarily through appropriately timed stimulation.

These results, taken together with the textile architecture, integrated diode-enhanced switching, high-performance current output, and demonstrated *in vivo* efficacy, represent substantial progress toward the practical clinical translation of self-powered therapeutic devices. Unlike earlier FES systems that required tethered power supplies, control electronics, or rechargeable batteries, the D-T-TENG achieves functional electrical stimulation purely through triboelectric harvesting and mechanical switching. This eliminates major barriers to clinical adoption, including the need for frequent battery charging, the risk of electrical hazard from complex electronics, and the comfort limitations imposed by bulky external controllers.

The SPETH glove emphasizes closed-loop feedback and sensory rehabilitation, making it particularly suitable for neurosensory recovery in stroke, peripheral nerve injury, and other conditions where restoring sensory perception is essential for motor recovery. The automatic adjustment of stimulation intensity and frequency based on user effort creates engaging biofeedback that encourages patient participation and provides natural reinforcement for increased therapeutic effort.

The D-T-TENG emphasizes clinician control and functional movement restoration, making it suitable for functional electrical stimulation applications where precise temporal and intensity control are essential for achieving coordinated movement patterns. The ability to selectively stimulate different muscle groups on demand enables restoration of complex movement sequences such as grasping, reaching, or walking.

Both systems operate completely without external power sources, achieving the ideal of truly autonomous wearable therapy. Both integrate harvesting, storage, control, and stimulation into textile-based architectures that maintain the comfort and wearability essential for therapeutic acceptance. Yet each optimizes different aspects of the design space SPETH prioritizes intuitive, user-responsive feedback. In contrast, D-T-TENG prioritizes precise clinician control, demonstrating that self-powered textile-based therapy is not monolithic but can be adapted to diverse clinical needs.

The energy harvesting systems illustrated in Fig. 11 represent a fundamental shift in how wearable therapeutic devices can be powered and controlled. Traditional medical devices emphasize reliability and performance, often at the cost of comfort and practicality for continuous, long-term wear. By harvesting energy directly from user motion or environmental energy sources, textile-based therapeutic systems eliminate the burden of power management, no charging, no battery replacement, no tethered power supplies, while maintaining complete functionality for therapeutic intervention.

This energy autonomy has profound implications for healthcare delivery. In clinical settings, self-powered therapeutic devices reduce infrastructure requirements and care provider burden. In home-care environments, self-powered devices eliminate a major barrier to patient compliance the need to remember to charge devices or maintain external power supplies. For patients in resource-limited or developing-world settings, self-powered therapeutic devices may be the only practical way to access sophisticated therapeutic technologies.

Moreover, integrating energy harvesting, storage, control, and therapeutic functions into unified textile architectures demonstrates that wearable therapeutics need not be complex, fragile, or uncomfortable. The most effective therapeutic systems may be those that seamlessly integrate with clothing, operate entirely passively without requiring user attention for power management, and provide intuitive feedback that naturally encourages patient engagement and compliance.

Yang *et al.*^119^ dipped cotton yarn into a mixture of pyrrole and MXene solution and then soaked it into 1 wt% sodium anthraquinone sulfonate (C_14_H_7_NaO_5_S) and 4 wt% ammonium persulfate ((NH_4_)_2_S_2_O_8_) to initiate the polymerization process. The areal capacitance of PPy/MXene@cotton electrode reached 455.9 mF cm^−2^ at a scan rate of 0.9 mA cm^−2^.

Li *et al.*^190^ fabricated MXene-coated cotton fabric (MCF) to use for a personal body thermal management system, where a supercapacitor device made with MXene-coated cotton electrode was used as the power source. For MXene coating, pretreated cotton fabric was dipped into 4 mg per L MXene solution for 5 minutes. The MCF electrode showed the highest capacitance of 208 mF cm^−2^ at the scan rate of 5 mV s^−1^. For real-life applications, MCF electrodes were used to prepare a symmetric device, with 3 devices connected in series using the bipolar concept. In addition, 4 bipolar units were also connected in parallel combination. Moreover, the joining of the MCF electrode and the supercapacitor device at the elbow demonstrated the feasibility of real-world applications.

To understand the real-world applications of supercapacitors, it is necessary to prepare supercapacitor devices, symmetric or asymmetric, using electrode materials. The electrochemical performance of the electrodes can only show the potential applications. However, the specific capacitance, energy density, and power density measurements of supercapacitor devices are necessary to understand real-life applications (Table 5).

**Table 5:** Real-life applications of supercapacitor devices prepared by MXene/textile and CNF-based composite electrodes

Electrode	Fabrication method	Capacitance	Real-world applications	Ref.
MXene/cotton fabric	Dip coating	208 mF cm^−2^ at 5 mV s^−1^	Body thermal management	190
PANI/MXene decorated PET fabric	Chemical polymerization and spray coating	647 mF cm^−2^ at 0.3 mA cm^−2^	Powering thermohygrometer	191
PPy/MXene/PEI-modified fabric	Chemical polymerization and dip coating	1295 mF cm^−2^ at 1 mA cm^−2^	Powering digital watch	120
rGO/Ti_3_C_2_T_*x*_ modified cotton fabric	Chemical reduction and spray coating	258 mF cm^−2^ at 5 mV s^−1^	Powering a watch	192
Ti_3_C_2_T_*x*_/carbon nanotube yarn	Biscrolling	3188 mF cm^−2^ at 2 mA cm^−2^	Powering digital watch, digital timer	159
Ti_3_C_2_@PPy–CMC	Chemical polymerization and blending	100 F g^−1^ at 1 A g^−1^	Glucose sensor	193
Ti_3_C_2_T_*x*_/TPU/polypyrrole (PPy) fiber	Chemical polymerization and wet-spinning	41.2 F g^−1^ at 5 mV s^−1^	Operating watch, electric meter	194

To move beyond a sequential recounting of individual studies, Table 6 evaluates the reviewed MXene/textile supercapacitor systems across five practical dimensions relevant to wearable therapeutics: the design rationale (necessity) behind each material choice, quantitative electrochemical performance, key limitations, device-safety considerations for on-body use, and the realistic human-application scenario each system supports. This multidimensional framing reveals that no single electrode system optimizes all criteria simultaneously cellulose- and cotton-based electrodes (*e.g.*, MXene-coated cotton fabric, PPy/MXene@cotton, Ti_3_C_2_T_*x*_/cotton yarn) offer superior breathability, biocompatibility, and skin safety, making them better suited to direct, prolonged skin contact. In contrast, synthetic-substrate or liquid-electrolyte systems (*e.g.*, PET-based, nylon-based) achieve comparable or higher capacitance but raise greater safety and comfort concerns for epidermal use, restricting them to enclosed or accessory-level power modules rather than direct therapeutic integration. By explicitly comparing necessity, limitations, and safety alongside raw performance metrics, this table provides a practical framework for selecting appropriate electrode systems based on the intended wearable therapeutic context, rather than performance alone.

**Table 6:** Multidimensional evaluation of MXene/textile supercapacitor systems for wearable therapeutics

Electrode system	Necessity (why this design)	Performance	Key limitations	Device safety considerations	Human application scenario	Ref.
MXene nanofiber-coated polyester yarn	Enables flexible, washable yarn-form electrodes for garment integration	Areal capacitance 18.39 mF cm^−2^ at 5 mV s^−1^; 98.2% retention after 6000 cycles	Polyester substrate lacks breathability/biocompatibility of cellulosic alternatives	Electrolyte containment critical to prevent skin contact with liquid electrolyte during wear	Woven into garment seams to power discrete sensors; not suited for direct prolonged skin contact	Shao *et al.*^142^
MXene/nylon nanoyarn	Combines electrospinning + wet-spinning for continuous conductive yarn	Specific capacitance 440 F cm^−3^ at 5 mV s^−1^ (LiClO_4_ electrolyte)	Uses liquid electrolyte (LiClO_4_), raising leakage/corrosion risk in wearable use	Requires robust encapsulation; liquid electrolyte unsuitable for direct epidermal application	Best suited for enclosed, non-skin-contact power modules (*e.g.*, belt- or strap-mounted units)	Levitt *et al.*^179^
PPy/MXene@cotton electrode	Leverages cotton's natural breathability and hydrophilicity for wearable comfort	Areal capacitance 455.9 mF cm^−2^ at 0.9 mA cm^−2^	Long-term oxidative stability of MXene under sweat/humidity not established	Cotton substrate improves skin compatibility, but electrode encapsulation still needed to prevent moisture-driven degradation products contacting skin	Suitable for integration into wearable bands or patches with a protective barrier layer	Yang *et al.*^119^
MXene-coated cotton fabric (MCF)	Enables direct-textile electrode for body-worn thermal management systems	Highest capacitance 208 mF cm^−2^ at 5 mV s^−1^; bipolar series/parallel device configuration validated at elbow joint	Performance under repeated flexing at joints not extensively cycle-tested	Series-connected bipolar units increase voltage; safe low-voltage design confirmed but requires insulation at joints to avoid short-circuit during movement	Demonstrated at elbow for on-body thermal management power supply	Li *et al.*^190^
PANI/MXene decorated PET fabric	Combines conducting polymer with MXene for enhanced capacitance	647 mF cm^−2^ at 0.3 mA cm^−2^	PET is non-biodegradable, non-breathable; less suitable for prolonged skin-adjacent wear	Rigid PET backing may cause discomfort/pressure points if worn against skin for extended periods	Powering ancillary devices (*e.g.*, thermohygrometer) rather than direct on-body therapeutic use	Zheng *et al.*^191^
PPy/MXene/PEI-modified fabric	High-capacitance system for powering small wearable electronics	1295 mF cm^−2^ at 1 mA cm^−2^	Multi-step chemical polymerization increases manufacturing complexity/cost	Residual oxidant/monomer chemicals require thorough washing before skin-adjacent use	Demonstrated powering a digital watch; suitable for accessory-level integration	Li *et al.*^120^
Ti_3_C_2_T_*x*_/CNT yarn (biscrolled)	No-binder design maximizes MXene loading and porosity	3188 mF cm^−2^ at 2 mA cm^−2^	Host material limited to CNT; CNT biocompatibility/inhalation-exposure risk during manufacturing must be managed	Fiber-form factor reduces bulk skin contact area; CNT leaching risk considered low once embedded in yarn matrix	Powering digital watch, digital timer; feasible for cordage-style integration into garments	Wang *et al.*^159^

### 7.2. Wearable thermal devices and thermotherapy

Textronics are widely used for healthcare systems to diagnose diseases. He *et al.*,^189^ La *et al.*,^195^ and Tsukada *et al.*^196^ demonstrated various diagnostic applications. In addition, wearable textile devices are not only used for diagnosis but also for therapeutic applications. Repon *et al.*^36^ reviewed therapeutic applications of smart textiles.

Regarding therapeutic applications, 2D Ti_3_C_2_T_*x*_-MXene materials are widely used due to their excellent photothermal and electrothermal properties. MXene exhibits localized surface plasmon resonance (LSPR), leading to strong absorption across the solar spectrum. Wang *et al.*^197^ explained the photothermal mechanisms. Therefore, MXene-modified yarn or fabric is used for thermotherapy applications, such as wound healing, antibacterial activity, warm, protective fabric for cold environments, and so on.

Gong *et al.*^198^ fabricated Ti_3_C_2_T_*x*_-MXene/polydopamine (PDA)/Ni^2+^ coated spandex yarn (MPNi@spandex) to use for wearable antibacterial therapy under near-infrared (NIR) irradiation. The coated yarn exhibited excellent photothermal performance. Fig. 12a demonstrates that the surface temperatures of the MXene-coated yarn increased significantly with increasing power density of NIR laser irradiation. Moreover, at the power density of 1.5 W cm^−2^, the coated yarn showed excellent photothermal stability, as shown in Fig. 12b.^198^

**Fig. 12:**
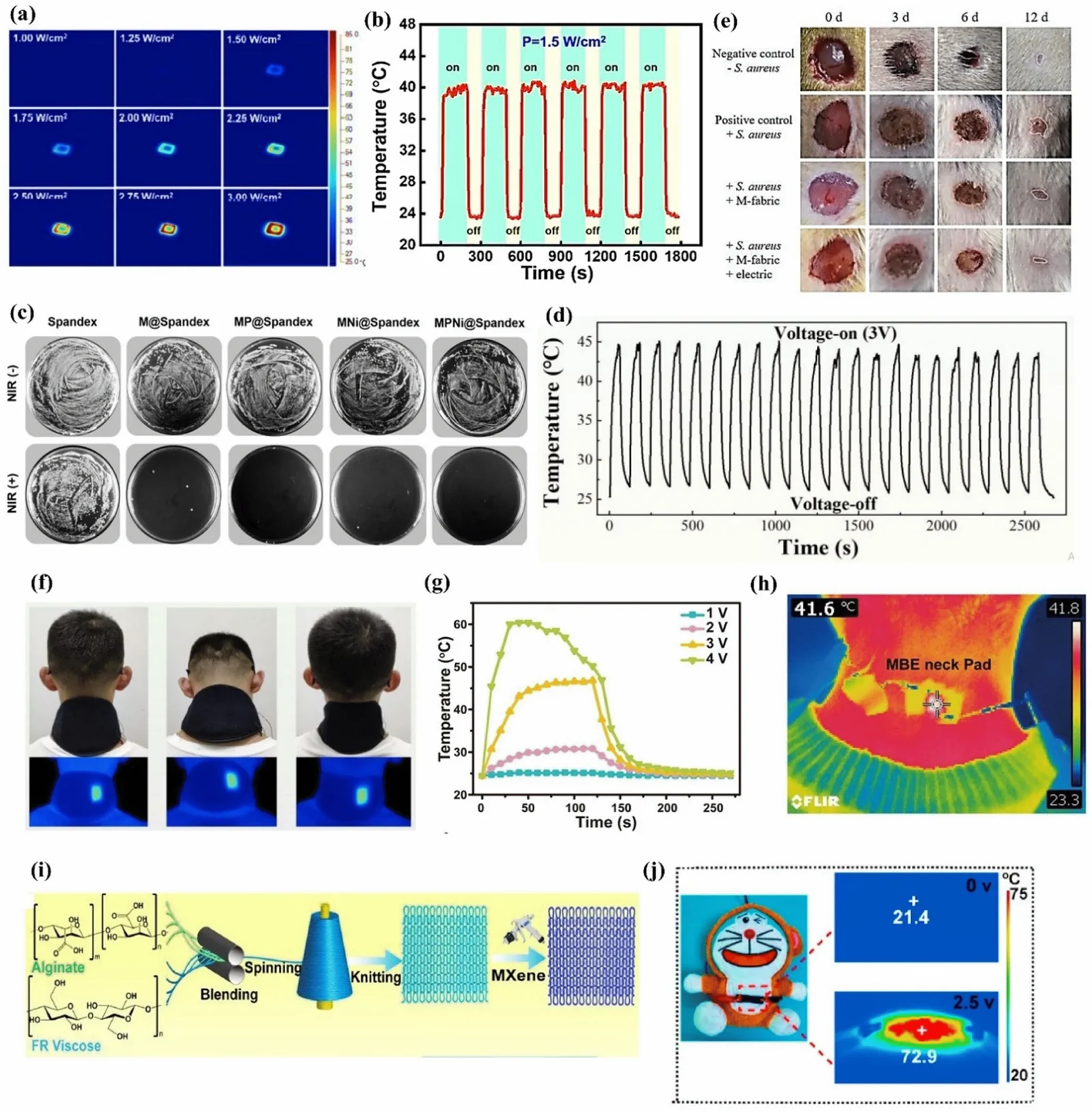
Photothermal and electrothermal behavior of MXene-modified textiles. (a) Surface temperature of MXene-coated spandex yarn under increasing NIR laser power, showing efficient Joule heating. (b) Photothermal stability of MXene-coated spandex at 1.5 W cm^−2^. (c) Antibacterial activity of spandex, MXene, MXene/PDA, MXene/Ni^2+^, and MXene/PDA/Ni^2+^ yarns with/without NIR irradiation, (a–c) have been adapted from ref. 198 with permission from American Chemical Society, copyright 2021. (d) Heat stability of MXene-coated nonwoven fabric under prolonged exposure. (e) Wound healing comparison of MXene-coated fabric with and without applied voltage. (f) Heating performance of MXene-coated fabric neck pad at 3 V, (d–f) have been adapted from ref. 202 with permission from American Chemical Society, copyright 2020. (g) Electrothermal response of MBE electrodes at 1–4 V. (h) Neck pad heating using MBE electrodes at 3 V, (g and h) have been adapted from ref. 200 with permission from American Chemical Society, copyright 2022. (i) Fabrication of MXene-coated alginate and viscose fiber fabrics. (j) Thermal imaging of MXene-modified fabric on a model object, (i and j) have been adapted from ref. 201 with permission from Elsevier, copyright 2022.

The excellent photothermal performance demonstrated the potential of Ti_3_C_2_T_*x*_-MXene-coated yarn for preparing wearable thermal devices. In addition, under laser irradiation, MPNi@spandex yarn showed excellent antibacterial activity (more than 99.9%) compared to MXene (M@spandex), MXene/PDA (MP@spandex), and MXene/Ni^2+^ (MNi@spandex) yarn, as demonstrated in Fig. 12c.^198^ This excellent antibacterial property was attributed to the sharp edges, anionic nature, and hydrophilicity of MXene nanosheets at the yarn surface.^198^

Zhao *et al.*^199^ used a dip coating and drying process to convert non-woven fabric into Ti_3_C_2_T_*x*_-MXene-loaded smart fabric. The adsorption of MXene onto the fabric surface is mainly caused due to the strong hydrophilic properties of the cellulosic fabric and the presence of functional groups on MXene. The MXene-coated fabric (M-fabric) exhibited excellent heating durability, as shown in Fig. 12d, demonstrating the potential for thermotherapy applications.

For bacterial-infected wound healing, Fig. 12e shows that the wounded areas of SD rats were almost recovered when M-fabric and electrical heating were used together.^199^ In addition, the neck guard pad made with M-fabric showed stable heat generation under an applied voltage of 3 V, as demonstrated in Fig. 12f.^199^ The cellulosic nonwoven substrate offers excellent biocompatibility and moisture management, making it ideal for wound-healing applications.

Song *et al.*^200^ prepared MXene-based epidermal electrodes (MBE) by dip-coating a cellulose film into a MXene dispersion. Fig. 12g shows that the temperature of the MBE film electrode reached its maximum position at a certain applied voltage within a very short time. To understand the potential theranostic application, an MBE neck cushion was worn. Fig. 12h shows that heating occurred around the neck muscle at 3 V. The use of grass-derived cellulose as the substrate demonstrates the versatility of cellulosic materials for biomedical applications, providing excellent skin compatibility and breathability.

He *et al.*^201^ blended sodium alginate (SA) fibers and flame-retardant viscose (FRV) fibers, followed by spinning to obtain the blended yarn. The yarn was knitted into fabric by a knitting machine. MXene solution was then sprayed onto the fabric, as shown in the fabrication process in Fig. 12i. The MXene-modified fabric demonstrated a wide temperature range across different applied voltages, demonstrating the heating requirements of wearable thermal devices. In addition, to understand human thermotherapy requirements, the MXene-modified fabric was placed on a toy. After applying 2.5 V, the surface temperature reached 72.9 °C, as illustrated in Fig. 12j. The incorporation of cellulose-based viscose fibers ensures comfort, breathability, and flame retardancy while maintaining excellent thermal performance.^201^Table 7 displays an overview of recent research progress on MXene- and CNF-based textronics in thermotherapy applications.

**Table 7:** Overview of recent research progress of MXene and CNF-based textronics in thermotherapy applications

MXene-based textronics	Fabrication strategy	Real-life applications	Ref.
MXene–amoxicillin–PVA nanofibrous membrane	Electrospinning	Antibacterial therapy, wound healing	203
Ti_3_C_2_T_*x*_/polydopamine/Ni^2+^ coated spandex yarn	Dipping and drying	Antibacterial therapy (>99.9% efficiency)	198
Ti_3_C_2_T_*x*_/polypropylene (PP) fabric	Dipping and drying	Antibacterial therapy	204
Ti_3_C_2_T_*x*_-coated grass-derived cellulose	Dip coating	Theranostic applications	200
AgNW–Ti_3_C_2_T_*x*_ coated non-woven fabric	Spray coating	Personal thermal management	205
Ti_3_C_2_T_*x*_/polydopamine decorated cotton fabric	Vacuum-assisted filtration	Personal thermal management	206
Ti_3_C_2_T_*x*_ coated fabric	Dip coating and drying	Antibacterial therapy, wound healing	151
MXene/PDMS/PDA/PU	Coating	Photothermal antibacterial activity	207
TPU/CS/MXene/AgNW film	Dip-coating and vacuum filtration	Photothermal therapy	208

### 7.3. Wearable sensors

At present, demand for textile-based wearable electronic devices is escalating. Due to the development of highly conductive MXene nanomaterials, a new form of smart textile wearable sensor has been built recently, thanks to the discovery of MXene materials.^15^ Jin and Bai^15^ comprehensively reviewed MXene-based sensors. When MXene is introduced into traditional textiles, sensor performance, such as sensitivity, stability, and response time, is significantly improved.^15^

In addition, lightweight, air permeability, high strength, and comfort are the ideal characteristics of textile-based wearable sensors. Furthermore, textile materials exhibit three distinct dimensional architectures: one-dimensional (*e.g.*, fiber or yarn), two-dimensional (*e.g.*, plane fabric), and three-dimensional (*e.g.*, spacer fabric). Therefore, it is possible to develop new high-performance sensor devices by using textile substrates.

MXene and CNF-based textile composite devices have high electrical conductivity, making them ideal candidates for wearable strain sensors. In addition to developing wearable gas-sensor devices, MXene- and CNF-based textronics are also widely used for humidity sensors. Liu *et al.*^209^ developed a vacuum-assisted layer-by-layer assembly technique to deposit electrically conductive substances (AgNW and MXene) on silk textiles. Moreover, the developed conductive fabric showed changes in electrical resistance under different moisture conditions, indicating its potential application for detecting human humidity (*i.e.*, sweating levels) to monitor human health. Furthermore, the MXene-modified silk textile showed stable, repeatable resistance changes during cyclic testing, indicating stable sensitivity and durability to moisture.^209^ Natural protein-based silk fibers, like cellulose, offer excellent biocompatibility and hygroscopic properties that enhance humidity-sensing capabilities.

## 8. Challenges and future perspectives

One of the most significant obstacles limiting the practical implementation of MXene-based therapeutic textiles is the oxidative degradation of MXene nanosheets during storage and long-term operation. Ti_3_C_2_T_*x*_ MXene is particularly susceptible to oxidation in aqueous and oxygen-containing environments, where dissolved oxygen and moisture gradually convert conductive Ti_3_C_2_T_*x*_ into electrically insulating TiO_2_. This degradation process is accelerated by elevated temperature, alkaline pH, structural defects, and light exposure, resulting in progressive deterioration of electrical conductivity, mechanical integrity, and electrochemical performance. Since wearable therapeutic devices often operate under humid physiological conditions for extended periods, improving oxidation resistance remains essential for ensuring long-term device reliability and clinical applicability.

Although considerable progress has been achieved in mitigating MXene oxidation through various stabilization strategies, MXene- and CNF-based textronics remain challenging. Polymer encapsulation using cellulose nanofibers, poly(vinyl alcohol), polyurethane, chitosan, and other biocompatible matrices effectively limits oxygen and moisture diffusion while preserving mechanical flexibility. Surface functionalization with antioxidants, reducing agents, or compatible polymers further suppresses oxidation by stabilizing surface termination groups and minimizing defect formation. Additional approaches, including freeze-drying, oxygen-free processing, vacuum or inert-gas storage, and incorporation of antioxidant molecules such as ascorbic acid or tannic acid, have also demonstrated significant improvements in storage stability. The combination of these approaches with CNF-based protective networks is particularly promising because cellulose nanofibers simultaneously provide mechanical reinforcement, improved dispersion, and partial environmental shielding without compromising flexibility or biocompatibility.

Exhibit remarkable potential for next-generation wearable electronics, several critical challenges must be resolved before large-scale commercialization can be realized. One of the most significant obstacles is the oxidation instability of MXenes, which severely limits their long-term performance in practical applications. Since MXene-modified fabrics are directly exposed to ambient conditions when worn on the human body, oxidation is highly probable. It can lead to rapid degradation of electrical conductivity and device reliability. This issue has therefore become a major bottleneck for MXene and CNF-based textronics and requires careful consideration in future research. Recent studies have explored high-temperature annealing as a strategy to improve MXene oxidation resistance; however, such processes may hinder scalable and cost-effective manufacturing of textile-based electronics. Alternatively, surface protection approaches using hydrophobic coatings, such as silicon-based materials or PDMS, have been proposed to limit environmental exposure. Luo *et al.*^210^ demonstrated that depositing a thin PDMS layer onto MXene-coated textiles *via* a simple dipping-and-drying process imparted superhydrophobicity and effectively protected MXenes from oxidation without compromising fabric breathability or mechanical strength. However, maintaining high electrical conductivity remains a critical requirement.

Scalability and manufacturability represent another key challenge for MXene and CNF-based textronics. From an industrial perspective, fabricating conductive yarns is considered more scalable than directly modifying finished fabrics, as conductive yarns can be readily integrated into existing textile manufacturing processes. Techniques such as wet-spinning, electrospinning, and solution coating have been employed to produce conductive fibers and yarns; however, achieving a balance between high electrical conductivity and mechanical robustness remains difficult. For example, in wet-spinning approaches, incorporating Ca^2+^ ions into the coagulation bath has been shown to enhance yarn toughness. Material selection is another decisive factor governing the functional properties of MXene and CNF-based textronics. Parameters such as MXene flake size, the incorporation of additional functional materials, fiber type, and fabric architecture all play critical roles in determining electrical, mechanical, and comfort-related characteristics. Cotton fibers are often favored for wearable electronics due to their softness and moisture permeability.

In contrast, high-strength fibers such as aramid are more suitable for demanding applications, such as military body armor.^163^ In environments requiring water resistance, such as diving or marine applications, hydrophobic surface modification becomes essential. Consequently, careful, application-specific material selection is necessary to fully exploit the potential of MXene–CNF hybrid textronics.

In the biomedical field, MXene and CNF-based textronics have attracted increasing attention for applications such as ECG, EMG, and other diagnostic systems.^189,196^ These skin-attachable devices must combine high flexibility, comfort, and long-term biocompatibility with reliable signal acquisition over extended periods of use. The inherent biocompatibility of cellulose nanofibers makes them particularly attractive for such applications, yet further comprehensive studies are needed to ensure safety, durability, and consistent performance under real-world conditions.

Looking ahead, several promising research directions emerge from the current literature on CNF-based e-textiles. Advancements in manufacturing processes are essential, including optimizing CNF extraction and modification to reduce costs; developing continuous coating and spinning techniques compatible with existing textile infrastructure; implementing robust quality control methods; and integrating with roll-to-roll processing for high-throughput production. Long-term durability and washability must also be systematically addressed through strategies such as covalent bonding between CNF and conductive fillers, development of protective yet flexible coatings, accelerated aging studies, and encapsulation approaches that preserve textile comfort while protecting conductive components.

Exploring novel material combinations offers further opportunities to enhance multifunctionality. Beyond Ti_3_C_2_T_*x*_, emerging MXene compositions such as Mo_2_TiC_2_ and Ti_4_N_3_,^14^ as well as other two-dimensional materials including phosphorene, silicene, and germanene, may provide unique electronic and sensing capabilities. Hybrid systems incorporating organic semiconductors or conducting polymers alongside CNF matrices could further expand performance envelopes. Application-specific development is particularly promising in healthcare, where CNF-based e-textiles can enable continuous health monitoring, smart wound dressings with antibacterial functionality,^198,199^ rehabilitation garments, and wearable drug delivery systems that leverage CNF's biocompatibility and porous structure.

Sustainability and circular economic considerations are increasingly important for future e-textile development. Strategies such as designing recyclable or compostable systems, using industrial waste streams as CNF sources, conducting life-cycle assessments, and designing for disassembly can significantly reduce environmental impact. In parallel, multifunctional architectures enabled by 3D printing of CNF-based inks, layer-by-layer assembly,^151,198^ biomimetic design principles, and gradient structures can unlock new levels of performance and integration. The incorporation of computational modeling, machine learning, and artificial intelligence offers powerful tools for accelerating materials discovery, process optimization, and device performance prediction. Future research should focus on quantitatively correlating oxidation kinetics with long-term device performance under realistic operating conditions, including repeated mechanical deformation, sweat exposure, sterilization, and prolonged storage. Standardized protocols for evaluating conductivity retention, oxidation rate, cyclic durability, and shelf life are also needed to facilitate meaningful comparisons among different CNF–MXene systems. Such studies will accelerate the translation of laboratory-scale prototypes into reliable, clinically applicable wearable therapeutic platforms.

## 9. Conclusion

Cellulose nanofibers represent a sustainable, high-performance, and versatile platform for the development of electronic textiles. Their combination of high mechanical strength, aqueous processability, biodegradability, and rich surface chemistry enables strong interfacial interactions and multifunctional performance. Numerous studies have demonstrated that CNF-based systems achieve high electrical and thermal conductivities,^103^ highly conductive fibers,^155^ sensitive, flexible sensors,^108^ high-capacitance energy storage devices,^166^ and effective thermal management.^190^ CNF serves as a structural binder, flexible substrate, functional component, and sustainable alternative to petroleum-based polymers, bridging the gap between natural materials and advanced electronics. The synergistic integration of CNF with MXenes, enabled by strong hydrogen-bonding interactions,^152^ produces composite systems with superior electrical stability and mechanical durability. As remaining challenges related to scalability, long-term stability, wash fastness, and cost are addressed through systematic research and engineering innovation, CNF-based e-textiles are poised to play a central role in the future of smart, comfortable, and environmentally responsible wearable electronics.

## Conflicts of interest

The authors declare that they have no conflict of interest.

## Abbreviations

AgNW: Silver nanowire
ANF: Aramid nanofiber
CF: Carbon fiber
CNC: Crystalline nanocellulose
CNFs: Cellulose nanofibers
CNT: Carbon nanotube
CSY: Core–sheath elastic yarn
CTE: Coefficients of thermal expansion
DMSO: Dimethyl sulfoxide
D-T-TENG: Diode-enhanced textile triboelectric nanogenerator
EMI: Electromagnetic interference
E-textiles: Electronic textiles
FEP: Fluorinated ethylene propylene
FRV: Flame-retardant viscose
GF: Gauge factor
GM: Gastrocnemius
HA: Sodium hyaluronate
h-BN: Hexagonal boron nitride
HC: High concentration
HF: Hydrofluoric acid
ILED: Inorganic light-emitting diode
*I*_sc_
: Short-circuit current
KM: Kevlar/MXene
LAA: l-Ascorbic acid
LC: Low concentration
LSPR: Localized surface plasmon resonance
MBE: MXene-based epidermal electrodes
MCCF: MXene-based Joule heating film
MCF: MXene-coated cotton fabric
MCSY: Modified cotton/spandex yarn
MF: MXene coated cotton fabric
M-fabric: MXene-coated fabric
MGP: MXene-glutaraldehyde polyvinyl alcohol
MSA: Methanesulfonic acid
MY: MXene coated cotton yarn
nC: Nanocoulombs
NIR: Near-infrared irradiation
P(VDF-TrFE): Poly(vinylidene fluoride-*co*-trifluoroethylene)
PAA: Poly(acrylic acid)
PANI: Polyaniline
PBS: Polyborosiloxane
PDA: Polydopamine
PDMS: Polydimethylsiloxane
PDT: Photodynamic therapy
PEO: Poly(ethylene oxide)
PET: Poly(ethylene terephthalate)
PP: Polypropylene
PPy: Polypyrrole
PS: Polystyrene
PTSA: *p*-Toluenesulfonic acid
PVA: Poly(vinyl alcohol)
Py: Pyrrole
*R*_c_
: Bending radius
RED: Reverse electrodialysis
rGO: Reduced graphene oxide
*R*_s_
: Sheet resistance
SA: Sodium alginate
SPETH: Self-powered electrotactile textile haptic
STDOC: Sodium taurodeoxycholate
TA: Tibialis anterior
TENG: Triboelectric nanogenerator
TFA: Trifluoroacetic acid
TPU: Thermoplastic polyurethane
TSP: Touchscreen panel
*V*_oc_
: Open-circuit voltage
WKF: Woven Kevlar fiber
WPU: Waterborne polyurethane
XPS: X-ray photoelectron spectroscopy

## Data Availability

All data generated or analyzed during this study are included in this manuscript.

## References

[cit1] Keum K., Kim J. W., Hong S. Y., Son J. G., Lee S. S., Ha J. S. (2020). Adv. Mater..

[cit2] Kwon K., Lee Y. J., Chung S., Lee J., Na Y., Kwon Y., Shin B., Bateman A., Lee J., Guess M. (2025). ACS Appl. Mater. Interfaces.

[cit3] Wang J., Lu C., Zhang K. (2020). Energy Environ. Mater..

[cit4] Xie M., Hisano K., Zhu M., Toyoshi T., Pan M., Okada S., Tsutsumi O., Kawamura S., Bowen C. (2019). Adv. Mater. Technol..

[cit5] Zhang Q., Gao X. W., Liu X., Mu J. J., Gu Q., Liu Z., Luo W. B. (2024). Battery Energy.

[cit6] Takei K., Honda W., Harada S., Arie T., Akita S. (2015). Adv. Healthcare Mater..

[cit7] Afroj S., Tan S., Abdelkader A. M., Novoselov K. S., Karim N. (2020). Adv. Funct. Mater..

[cit8] Karim N., Afroj S., Tan S., He P., Fernando A., Carr C., Novoselov K. S. (2017). ACS Nano.

[cit9] Di J., Zhang X., Yong Z., Zhang Y., Li D., Li R., Li Q. (2016). Adv. Mater..

[cit10] Huang X., Li Z., Li Y., Wu X., Liu C., Xie H., Yu W. (2024). ACS Appl. Nano Mater..

[cit11] Lim T., Kim H. J., Won S., Kim C. H., Yoo J., Lee J. H., Son K. S., Nam I.-W., Kim K., Yeo S. Y. (2023). ACS Appl. Nano Mater..

[cit12] Roslan N. C. , Aizamddin M. F., Ruzaidi D. A. A., Ayub A. N., Asri N. A. N., Jani N. A., Shafiee S. A. and Mahat M. M., in Conjugated Polymers for Next-Generation Applications, Elsevier, 2022, pp. 325–359

[cit13] Allison L., Hoxie S., Andrew T. L. (2017). Chem. Commun..

[cit14] Ma C., Ma M. G., Si C., Ji X. X., Wan P. (2021). Adv. Funct. Mater..

[cit15] Jin C., Bai Z. (2022). ACS Sens..

[cit16] Ghobashy M. M., Baata M., Almoiqli M. S., Haque M. A., Algethami F. K., Siddiq A., Attia M. S. (2026). RSC Adv..

[cit17] Algrafy E., Ghobashy M. M., Baata M., Almoiqli M. S., Siddiq A., Madani M., Algethami F. K., Attia M. S. (2026). J. Alloys Compd..

[cit18] Ciancibello J., King K., Meghrazi M. A., Padmanaban S., Levy T., Ramdeo R., Straka M., Bouton C. (2019). Bioelectronic Medicine.

[cit19] Wang Z., Zhao X., Yan K., Zhang P., Zhang S., Fan H. (2025). Mater. Sci. Eng., R.

[cit20] Li Z., Ma Y., Zhang K., Wan J., Zhao D., Pi Y., Chen G., Zhang J., Tang W., Lin L. (2023). Adv. Funct. Mater..

[cit21] Fu M., Zhang J., Jin Y., Zhao Y., Huang S., Guo C. F. (2020). Advanced Science.

[cit22] Lin R., Kim H.-J., Achavananthadith S., Xiong Z., Lee J. K., Kong Y. L., Ho J. S. (2022). Nat. Commun..

[cit23] Uzun S., Seyedin S., Stoltzfus A. L., Levitt A. S., Alhabeb M., Anayee M., Strobel C. J., Razal J. M., Dion G., Gogotsi Y. (2019). Adv. Funct. Mater..

[cit24] Song Y., Min J., Gao W. (2019). ACS Nano.

[cit25] Lee S. G., Yu K. J., Won S. M., Yoo J.-Y. (2025). Int. J. Extreme Manuf..

[cit26] Ahmed A., Hossain M. M., Adak B., Mukhopadhyay S. (2020). Chem. Mater..

[cit27] Barman J., Tirkey A., Batra S., Paul A. A., Panda K., Deka R., Babu P. J. (2022). Mater. Today Commun..

[cit28] Lee B. W., Kang J., Yoo J.-Y., Won S. M. (2025). npj Flexible Electron..

[cit29] Zhang C. J., Nicolosi V. (2019). Energy Storage Materials.

[cit30] Tang H., Hu Q., Zheng M., Chi Y., Qin X., Pang H., Xu Q. (2018). Prog. Nat. Sci.:Mater. Int..

[cit31] Chen G., Li Y., Bick M., Chen J. (2020). Chem. Rev..

[cit32] Usman K. A. S., Qin S., Henderson L. C., Zhang J., Hegh D. Y., Razal J. M. (2021). Mater. Horiz..

[cit33] Bhardwaj N., Kundu S. C. (2010). Biotechnol. Adv..

[cit34] Rohani Shirvan A., Nouri A., Sutti A. (2022). Eur. Polym. J..

[cit35] Libanori A., Chen G., Zhao X., Zhou Y., Chen J. (2022). Nat. Electron..

[cit36] Repon M. R., Mikučionienė D., Milašius R., Paul T. K., Ahmed C. M., Hussain S. Z., Haji A. (2023). Mater. Today Commun..

[cit37] Thakur A., Chandran B.S. N., Davidson K., Bedford A., Fang H., Im Y., Kanduri V., Wyatt B. C., Nemani S. K., Poliukhova V., Kumar R., Fakhraai Z., Anasori B. (2023). Small Methods.

[cit38] Zhao D., Zhu Y., Cheng W., Chen W., Wu Y., Yu H. (2021). Adv. Mater..

[cit39] Zhang Y., Zhang C., Wang Y. (2021). Nanoscale Adv..

[cit40] Lomov S. V., Huysmans G., Verpoest I. (2001). Text. Res. J..

[cit41] Messnaoui R., El Bakkali M., Elkhaoudi M., Soulhi A., Cherkaoui O. (2024). Autex Res. J..

[cit42] Euler L., Guo L., Persson N.-K. (2022). Text. Res. J..

[cit43] Li Q., Tao X. M. (2014). Proc. R. Soc. A.

[cit44] Chen J., He T., Du Z., Lee C. (2023). Nano Energy.

[cit45] Chen K., Hu H., Zeng Y., Pan H., Wang S., Zhang Y., Shi L., Tan G., Pan W., Liu H. (2022). Eur. Polym. J..

[cit46] Walther M., Vestweber P. K., Kühn S., Rieger U., Schäfer J., Münch C., Vogel-Kindgen S., Planz V., Windbergs M. (2023). Mol. Pharm..

[cit47] Samanta A., Bordes R. (2020). RSC Adv..

[cit48] Hong X., Sun W., Zhang S., Tang Z., Zhou M., Guo S., Guo X., Zhao W., Wang X., Chen H. (2024). Adv. Fiber Mater..

[cit49] Zhang X., Wang X., Lei Z., Wang L., Tian M., Zhu S., Xiao H., Tang X., Qu L. (2020). ACS Appl. Mater. Interfaces.

[cit50] Serrano-Claumarchirant J. F., Muñoz-Espí R., Cantarero A., Culebras M., Gómez C. M. (2023). Coatings.

[cit51] Chatterjee S., Chi-leung HUI P. (2019). Molecules.

[cit52] Ferri A., Plutino M. R., Rosace G. (2019). AIP Conf. Proc..

[cit53] Hsu P.-C., Liu C., Song A. Y., Zhang Z., Peng Y., Xie J., Liu K., Wu C.-L., Catrysse P. B., Cai L. (2017). Sci. Adv..

[cit54] Evanghelidis A., Beregoi M., Diculescu V. C., Galatanu A., Ganea P., Enculescu I. (2018). Sci. Rep..

[cit55] Perera A. S., Jackson R. J., Bristow R. M., White C. A. (2022). Sci. Rep..

[cit56] Tang J., Tong D., Tang C., Wang S., Dang G., Zhao W., Sun S., Zhao C. (2025). Macromol. Biosci..

[cit57] Lin Z.-W., Chen Y.-L., Lei W.-L., Ku C.-Y., Su P.-Y., Tai L.-C., Tsai T.-H., Liao Y.-T., Chuang A. E.-Y., Huang W.-C. (2025). J. Controlled Release.

[cit58] Sun X.-Z., Wang X., Wu J.-Z., Li S.-D. (2017). Appl. Surf. Sci..

[cit59] Mostafalu P., Kiaee G., Giatsidis G., Khalilpour A., Nabavinia M., Dokmeci M. R., Sonkusale S., Orgill D. P., Tamayol A., Khademhosseini A. (2017). Adv. Funct. Mater..

[cit60] Tan S., Afroj S., Li D., Islam M. R., Wu J., Cai G., Karim N., Zhao Z. (2023). Iscience.

[cit61] Mandriota G., Grandolfo A., Striani R., Panniello A., Bianco G. V., Milella A., Mele C., Comparelli R., Greco A., Striccoli M. (2025). Appl. Surf. Sci..

[cit62] Wang Z., Wang X., Cui J., Shi Z., Yan F., Han Y., Li Z., Zhu Z. (2023). Analytica.

[cit63] Hashem H. M., Motawea A., Kamel A. H., Bary E. A., Hassan S. S. (2022). Sci. Rep..

[cit64] Wang Z., Wu T., Wang Z., Zhang T., Chen M., Zhang J., Liu L., Qi M., Zhang Q., Yang J. (2020). Nat. Commun..

[cit65] Oh H. J., Han S. H., Kim S. S. (2014). J. Polym. Sci., Part B: Polym. Phys..

[cit66] Jalalah M., Ahmad A., Saleem A., Qadir M. B., Khaliq Z., Khan M. Q., Nazir A., Faisal M., Alsaiari M., Irfan M. (2022). Membranes.

[cit67] Sanchaniya J. V., Lasenko I., Kanukuntla S. P., Mannodi A., Viluma-Gudmona A., Gobins V. (2023). Nanomaterials.

[cit68] Orf N. D., Shapira O., Sorin F., Danto S., Baldo M. A., Joannopoulos J. D., Fink Y. (2011). Proc. Natl. Acad. Sci. U. S. A..

[cit69] Yan W., Dong C., Xiang Y., Jiang S., Leber A., Loke G., Xu W., Hou C., Zhou S., Chen M. (2020). Mater. Today.

[cit70] Canales A., Jia X., Froriep U. P., Koppes R. A., Tringides C. M., Selvidge J., Lu C., Hou C., Wei L., Fink Y. (2015). Nat. Biotechnol..

[cit71] Abouraddy A., Bayindir M., Benoit G., Hart S., Kuriki K., Orf N., Shapira O., Sorin F., Temelkuran B., Fink Y. (2007). Nat. Mater..

[cit72] Seyedin S., Carey T., Arbab A., Eskandarian L., Bohm S., Kim J. M., Torrisi F. (2021). Nanoscale.

[cit73] Cheng X., Jaluria Y. (2005). Int. J. Heat Mass Transfer.

[cit74] Shabahang S., Clouser F., Shabahang F., Yun S. H. (2021). Adv. Opt. Mater..

[cit75] Khakse P., Dangers F., Elsersawy R., Khondoker M. A. H. (2024). Materials.

[cit76] Borges C., Marques E., Carbas R., Ueffing C., Weißgraeber P., Silva L. d. (2021). Proc. Inst. Mech. Eng., Part C.

[cit77] Lee H. M., Kim M.-S., Hong W. S. (2023). Journal of Welding and Joining.

[cit78] Sloboda L., Kennedy T., Liu J., Ramjattan D., Wang N., Turbini L. J. (2013). Journal of Surface Mount Technology.

[cit79] Yan L., Yao J., Dai Y., Zhang S., Bai W., Gao K., Yang H., Wang Y. (2021). Electronics.

[cit80] Fahim A. , Hasan S. K., Suhling J. C. and Lall P., 2020 IEEE 70th Electronic Components and Technology Conference (ECTC), 2020, pp. 1486–1495

[cit81] Wang Y., Ying L., Sun R., Li C., Ding Z., Wang Z. (2022). Coatings.

[cit82] Li Y., Zhang X., Si Y., Yu J., Ding B. (2022). ACS Appl. Polym. Mater..

[cit83] Sheng J., Zhang M., Xu Y., Yu J., Ding B. (2016). ACS Appl. Mater. Interfaces.

[cit84] Gu X., Li N., Gu H., Xia X., Xiong J. (2018). J. Appl. Polym. Sci..

[cit85] Sheng J. , Zhao J., Yu X., Liu L., Yu J. and Ding B., in Electrospinning: Nanofabrication and Applications, Elsevier, 2019, pp. 543–570

[cit86] Yang H., Cheng L., Yang Q., Farooq A., Ying L., Wang P., Wang Z. (2023). Appl. Surf. Sci..

[cit87] Naguib M., Kurtoglu M., Presser V., Lu J., Niu J., Heon M., Hultman L., Gogotsi Y., Barsoum M. W. (2011). Adv. Mater..

[cit88] Downes M., Shuck C. E., McBride B., Busa J., Gogotsi Y. (2024). Nat. Protoc..

[cit89] Anasori B. , Lukatskaya M. R. and Gogotsi Y., in MXenes, Jenny Stanford Publishing, 2023, pp. 677–722

[cit90] Sun Z., Ahmad M., Gao Z., Shan Z., Xu L., Wang S., Jin Y. (2022). Sustainable Energy Fuels.

[cit91] Liu D., Li L., Gong Q., Du G., Xue Y., Li G., Li S. (2025). Appl. Surf. Sci..

[cit92] Chen R., Tang H., Dai Y., Zong W., Zhang W., He G., Wang X. (2022). ACS Nano.

[cit93] Wu Y., Xin W., Kong X.-Y., Chen J., Qian Y., Sun Y., Zhao X., Chen W., Jiang L., Wen L. (2020). Mater. Horiz..

[cit94] Liu P., Zhou T., Yang L., Zhu C., Teng Y., Kong X.-Y., Wen L. (2021). Energy Environ. Sci..

[cit95] Sun R., Xia Y., Peng M., Wang Y., Zhang J., Liu Q., Zhang S., Wang J. (2025). Colloids Surf., A.

[cit96] Meng W., Liu X., Song H., Xie Y., Shi X., Dargusch M., Chen Z.-G., Tang Z., Lu S. (2021). Nano Today.

[cit97] Khudiyev T., Lee J. T., Cox J. R., Argentieri E., Loke G., Yuan R., Noel G. H., Tatara R., Yu Y., Logan F. (2020). Adv. Mater..

[cit98] Dong Y., Xie Z., Xu F., Wang Z., Tu Z., Zhang Y. (2025). Polym. Compos..

[cit99] Cheng Y., Zhu M., Chi M., Lai Y., Li B., Qian R., Chen Z., Zhao G. (2024). ACS Appl. Mater. Interfaces.

[cit100] Inman A., Hryhorchuk T., Bi L., Wang R. J., Greenspan B., Tabb T., Gallo E. M., VahidMohammadi A., Dion G., Danielescu A. (2023). J. Mater. Chem. A.

[cit101] Wang L., Tian M., Zhang Y., Sun F., Qi X., Liu Y., Qu L. (2020). J. Mater. Sci..

[cit102] Ji S., Hyun B. G., Kim K., Lee S. Y., Kim S.-H., Kim J.-Y., Song M. H., Park J.-U. (2016). NPG Asia Mater..

[cit103] Qian K., Zhou Q., Thaiboonrod S., Fang J., Miao M., Wu H., Cao S., Feng X. (2022). J. Colloid Interface Sci..

[cit104] Wang Z.-G., Gong F., Yu W.-C., Huang Y.-F., Zhu L., Lei J., Xu J.-Z., Li Z.-M. (2018). Compos. Sci. Technol..

[cit105] Wang Q. W., Zhang H. B., Liu J., Zhao S., Xie X., Liu L., Yang R., Koratkar N., Yu Z. Z. (2019). Adv. Funct. Mater..

[cit106] Hazarika A., Deka B. K., Seo J., Jeong H. E., Park Y.-B., Park H. W. (2021). Nano Energy.

[cit107] Yan J., Ma Y., Zhang C., Li X., Liu W., Yao X., Yao S., Luo S. (2018). RSC Adv..

[cit108] Li Q., Yin R., Zhang D., Liu H., Chen X., Zheng Y., Guo Z., Liu C., Shen C. (2020). J. Mater. Chem. A.

[cit109] Zhao W., Zheng Y., Qian J., Zhaofa Z., Jin Z., Qiu H., Zhu C., Hong X. (2022). J. Alloys Compd..

[cit110] Liu X., Du X., Li L., Cao Y., Yang Y., Wang W., Wang J. (2022). Composites, Part A.

[cit111] Wei X., Aouraghe M. A., Pang S., Farha F. I., Saleemi S., Zhang K., Liu W., Xu F. (2022). J. Ind. Text..

[cit112] Yin Z., Jian M., Wang C., Xia K., Liu Z., Wang Q., Zhang M., Wang H., Liang X., Liang X., Long Y., Yu X., Zhang Y. (2018). Nano Lett..

[cit113] Dong Y., Xie Z., Xu F., Wang Z., Tu Z., Zhang Y. (2025). Polym. Compos..

[cit114] Lu W., Yu P., Jian M., Wang H., Wang H., Liang X., Zhang Y. (2020). ACS Appl. Mater. Interfaces.

[cit115] Usman K. A. S., Qin S., Henderson L. C., Zhang J., Hegh D. Y., Razal J. M. (2021). Mater. Horiz..

[cit116] Hatter C. B., Sarycheva A., Levitt A., Anasori B., Nataraj L., Gogotsi Y. (2020). C.

[cit117] Liu X., Jin X., Li L., Wang J., Yang Y., Cao Y., Wang W. (2020). J. Mater. Chem. A.

[cit118] Zou S., Li D., He C., Wang X., Cheng D., Cai G. (2023). ACS Appl. Mater. Interfaces.

[cit119] Yang L., Lin F., Zabihi F., Yang S., Zhu M. (2021). Int. J. Biol. Macromol..

[cit120] Li X., Hao J., Liu R., He H., Wang Y., Liang G., Liu Y., Yuan G., Guo Z. (2020). Energy Storage Materials.

[cit121] Yin G., Wang Y., Wang W., Yu D. (2020). Colloids Surf., A.

[cit122] Aziz F., Ismail A. F. (2015). Mater. Sci. Semicond. Process..

[cit123] Zabihi F., Xie Y., Gao S., Eslamian M. (2015). Appl. Surf. Sci..

[cit124] Bishop J. E., Smith J. A., Lidzey D. G. (2020). ACS Appl. Mater. Interfaces.

[cit125] Zhang Y. Z., Wang Y., Jiang Q., El-Demellawi J. K., Kim H., Alshareef H. N. (2020). Adv. Mater..

[cit126] Mendhe A. C. , in Simple Chemical Methods for Thin Film Deposition: Synthesis and Applications, Springer, 2023, pp. 387–424

[cit127] Nandy S. and Chae K. H., in Ferrite Nanostructured Magnetic Materials, Elsevier, 2023, pp. 309–334

[cit128] Ahn S., Han T. H., Maleski K., Song J., Kim Y. H., Park M. H., Zhou H., Yoo S., Gogotsi Y., Lee T. W. (2020). Adv. Mater..

[cit129] Hall D. B., Underhill P., Torkelson J. M. (1998). Polym. Eng. Sci..

[cit130] Purbayanto M. A. K., Bury D., Chandel M., Shahrak Z. D., Mochalin V. N., Wójcik A., Moszczynska D., Wojciechowska A., Tabassum A., Naguib M. (2023). ACS Appl. Mater. Interfaces.

[cit131] He W., Sohn M., Ma R., Kang D. J. (2020). Nano Energy.

[cit132] Tian P., Liu X. (2015). Regener. Biomater..

[cit133] Yilbas B. S. , Al-Sharafi A. and Ali H., Self-cleaning of Surfaces and Water Droplet Mobility, Elsevier, 2019

[cit134] Tan G. Z., Zhou Y. (2020). Int. J. Polym. Mater. Polym. Biomater..

[cit135] Li Y., Zhu J., Cheng H., Li G., Cho H., Jiang M., Gao Q., Zhang X. (2021). Adv. Mater. Technol..

[cit136] Huang Z.-M., Zhang Y.-Z., Kotaki M., Ramakrishna S. (2003). Compos. Sci. Technol..

[cit137] Taylor G. I. (1969). Proceedings of the Royal Society of London. A. Mathematical and Physical Sciences.

[cit138] Levitt A. S., Alhabeb M., Hatter C. B., Sarycheva A., Dion G., Gogotsi Y. (2019). J. Mater. Chem. A.

[cit139] Fu X., Li L., Chen S., Xu H., Li J., Shulga V., Han W. (2021). J. Colloid Interface Sci..

[cit140] Mayerberger E. A., Street R. M., McDaniel R. M., Barsoum M. W., Schauer C. L. (2018). RSC Adv..

[cit141] Mayerberger E. A., Urbanek O., McDaniel R. M., Street R. M., Barsoum M. W., Schauer C. L. (2017). J. Appl. Polym. Sci..

[cit142] Shao W., Tebyetekerwa M., Marriam I., Li W., Wu Y., Peng S., Ramakrishna S., Yang S., Zhu M. (2018). J. Power Sources.

[cit143] Shirvan A. R., Nouri A., Sutti A. (2022). Eur. Polym. J..

[cit144] Qin S., Usman K. A. S., Hegh D., Seyedin S., Gogotsi Y., Zhang J., Razal J. M. (2021). ACS Appl. Mater. Interfaces.

[cit145] Seyedin S., Yanza E. R. S., Razal J. M. (2017). J. Mater. Chem. A.

[cit146] Zhang J., Seyedin S., Qin S., Wang Z., Moradi S., Yang F., Lynch P. A., Yang W., Liu J., Wang X. (2019). Small.

[cit147] Cheng B., Wu P. (2021). ACS Nano.

[cit148] Wang L., Zhang M., Yang B., Tan J. (2021). ACS Appl. Mater. Interfaces.

[cit149] Yue L., Gong M., Wang J., Ma S., Chen Q., Kong X., Lin X., Zhang L., Wu Z., Wang D. (2023). ACS Mater. Lett..

[cit150] Usman K. A. S., Zhang J., Qin S., Yao Y., Lynch P. A., Mota-Santiago P., Naebe M., Henderson L. C., Hegh D., Razal J. M. (2022). J. Mater. Chem. A.

[cit151] Repon M. R., Mikučionienė D., Paul T. K., Al-Humaidi J. Y., Rahman M. M., Islam T., Shukhratov S. (2024). RSC Adv..

[cit152] Zhao X., Zhang J., Lv K., Kong N., Shao Y., Tao J. (2022). Carbon.

[cit153] Wu G., Yang Z., Zhang Z., Ji B., Hou C., Li Y., Jia W., Zhang Q., Wang H. (2021). Electrochim. Acta.

[cit154] Zheng T., Zhang X., Li Y., Zhu Y., Yan W., Zhao Z., Zhang L., Bai C., Wang X. (2023). Mater. Lett..

[cit155] Eom W., Shin H., Ambade R. B., Lee S. H., Lee K. H., Kang D. J., Han T. H. (2020). Nat. Commun..

[cit156] Zhou T., Yu Y., He B., Wang Z., Xiong T., Wang Z., Liu Y., Xin J., Qi M., Zhang H. (2022). Nat. Commun..

[cit157] Wu H., Zhang Y., Kjøniksen A. L., Zhou X., Zhou X. (2021). Adv. Funct. Mater..

[cit158] Yu C., Gong Y., Chen R., Zhang M., Zhou J., An J., Lv F., Guo S., Sun G. (2018). Small.

[cit159] Wang Z., Qin S., Seyedin S., Zhang J., Wang J., Levitt A., Li N., Haines C., Ovalle-Robles R., Lei W. (2018). Small.

[cit160] Meena J. S., Choi S. B., Jung S.-B., Kim J.-W. (2022). Appl. Mater. Today.

[cit161] Levitt A., Zhang J., Dion G., Gogotsi Y., Razal J. M. (2020). Adv. Funct. Mater..

[cit162] De Silva R., Vongsanga K., Wang X., Byrne N. (2016). Cellu.

[cit163] Qian Y., Zhong J., Ou J. (2023). Polym. Compos..

[cit164] Sinha S. K., Bansal P., Maity S. (2017). J. Inst. Eng. (India):Ser. E.

[cit165] Huang L.-Z., Yuan Q., Ji X.-X., Li D.-D., Zhang W., Guo W.-Y., Qi M.-Y., Ma M.-G. (2022). Cellu.

[cit166] Fan W., Wang Q., Rong K., Shi Y., Peng W., Li H., Guo Z., Xu B. B., Hou H., Algadi H., Ge S. (2023). Nano-Micro Lett..

[cit167] Thakur A., Chandran BS N., Davidson K., Bedford A., Fang H., Im Y., Kanduri V., Wyatt B. C., Nemani S. K., Poliukhova V. (2023). Small Methods.

[cit168] Driscoll N., Richardson A. G., Maleski K., Anasori B., Adewole O., Lelyukh P., Escobedo L., Cullen D. K., Lucas T. H., Gogotsi Y. (2018). ACS Nano.

[cit169] Ghidiu M. , Lukatskaya M. R., Zhao M.-Q., Gogotsi Y. and Barsoum M. W., in MXenes, Jenny Stanford Publishing, 2023, pp. 379–399

[cit170] Maleski K., Ren C. E., Zhao M.-Q., Anasori B., Gogotsi Y. (2018). ACS Appl. Mater. Interfaces.

[cit171] He N., Patil S., Qu J., Liao J., Zhao F., Gao W. (2020). ACS Appl. Energy Mater..

[cit172] Peng Y.-Y., Akuzum B., Kurra N., Zhao M.-Q., Alhabeb M., Anasori B., Kumbur E. C., Alshareef H. N., Ger M.-D., Gogotsi Y. (2016). Energy Environ. Sci..

[cit173] Zhang H., Ji H., Chen J., Wang N., Xiao H. (2022). Ind. Crops Prod..

[cit174] Ma C., Yuan Q., Du H., Ma M.-G., Si C., Wan P. (2020). ACS Appl. Mater. Interfaces.

[cit175] Zhao X.-F., Wen X.-H., Zhong S.-L., Liu M.-Y., Liu Y.-H., Yu X.-B., Ma R.-G., Zhang D. W., Wang J.-C., Lu H.-L. (2021). ACS Appl. Mater. Interfaces.

[cit176] Xu Y., Tao Y., Zheng X., Ma H., Luo J., Kang F., Yang Q.-H. (2015). Adv. Mater..

[cit177] Qin K., Kang J., Li J., Shi C., Li Y., Qiao Z., Zhao N. (2015). ACS Nano.

[cit178] Tang H., Hu Q., Zheng M., Chi Y., Qin X., Pang H., Xu Q. (2018). Prog. Nat. Sci.:Mater. Int..

[cit179] Levitt A., Seyedin S., Zhang J., Wang X., Razal J. M., Dion G., Gogotsi Y. (2020). Small.

[cit180] Liu J., Zhang J., Liu J., Sun W., Li W., Shen H., Wang L., Li G. (2023). Materials.

[cit181] Zhang Q., Soham D., Liang Z., Wan J. (2025). Med-X.

[cit182] Dionisi A., Marioli D., Sardini E., Serpelloni M. (2016). IEEE Trans. Instrum. Meas..

[cit183] Pu X., Li L., Liu M., Jiang C., Du C., Zhao Z., Hu W., Wang Z. L. (2016). Adv. Mater..

[cit184] Pu X., Hu W., Wang Z. L. (2018). Small.

[cit185] Ko Y., Lee S., Kwon C. H., Lee S. W., Cho J. (2021). Adv. Energy Mater..

[cit186] Xu G., Wang H., Zhao G., Fu J., Yao K., Jia S., Shi R., Huang X., Wu P., Li J. (2025). Sci. Adv..

[cit187] Bolognini N., Russo C., Edwards D. J. (2016). Restor. Neurol. Neurosci..

[cit188] Seim C. E., Wolf S. L., Starner T. E. (2021). Journal of NeuroEngineering and Rehabilitation.

[cit189] He T., Wang H., Wang J., Tian X., Wen F., Shi Q., Ho J. S., Lee C. (2019). Advanced Science.

[cit190] Li J., Chen J., Wang H., Xiao X. (2021). ChemElectroChem.

[cit191] Zheng X., Wang Y., Nie W., Wang Z., Hu Q., Li C., Wang P., Wang W. (2022). Composites, Part A.

[cit192] Zheng X., Nie W., Hu Q., Wang X., Wang Z., Zou L., Hong X., Yang H., Shen J., Li C. (2021). Mater. Des..

[cit193] Vaghasiya J. V., Mayorga-Martinez C. C., Vyskočil J., Pumera M. (2022). Biosens. Bioelectron..

[cit194] Zhang J., Wang X., Hang G., Zhang W., Zheng Z., Duan J., Liu Z. (2024). Compos. Commun..

[cit195] La T., Qiu S., Scott D., Bakhtiari R., Kuziek J., Mathewson K., Rieger J., Chung H. (2018). Adv. Healthcare Mater..

[cit196] Tsukada Y. T., Tokita M., Murata H., Hirasawa Y., Yodogawa K., Iwasaki Y.-k., Asai K., Shimizu W., Kasai N., Nakashima H. (2019). Heart Vessels.

[cit197] Wang J., Shen M., Liu Z., Wang W. (2022). Nano Energy.

[cit198] Gong M., Yue L., Kong J., Lin X., Zhang L., Wang J., Wang D. (2021). ACS Appl. Mater. Interfaces.

[cit199] Zhao X., Wang L.-Y., Tang C.-Y., Zha X.-J., Liu Y., Su B.-H., Ke K., Bao R.-Y., Yang M.-B., Yang W. (2020). ACS Nano.

[cit200] Song D., Ye G., Zhao Y., Zhang Y., Hou X., Liu N. (2022). ACS Nano.

[cit201] He G., Wang L., Bao X., Lei Z., Ning F., Li M., Zhang X., Qu L. (2022). Composites, Part B.

[cit202] Zhao X., Wang L.-Y., Tang C.-Y., Zha X.-J., Liu Y., Su B.-H., Ke K., Bao R.-Y., Yang M.-B., Yang W. (2020). ACS Nano.

[cit203] Xu X., Wang S., Wu H., Liu Y., Xu F., Zhao J. (2021). Colloids Surf., B.

[cit204] Purbayanto M. A. K., Jakubczak M., Bury D., Nair V. G., Birowska M., Moszczyńska D., Jastrzebska A. (2022). ACS Appl. Nano Mater..

[cit205] Liu X., Miao J., Fan Q., Zhang W., Zuo X., Tian M., Zhu S., Zhang X., Qu L. (2021). ACS Appl. Mater. Interfaces.

[cit206] Yan B., Zhou M., Yu Y., Xu B., Cui L., Wang Q., Wang P. (2022). Composites, Part A.

[cit207] Wang X., Tao Y., Pan S., Fang X., Lou C., Xu Y., Wu J., Sang M., Lu L., Gong X. (2022). npj Flexible Electron..

[cit208] Chao M., Di P., Yuan Y., Xu Y., Zhang L., Wan P. (2023). Nano Energy.

[cit209] Liu L. X., Chen W., Zhang H. B., Wang Q. W., Guan F., Yu Z. Z. (2019). Adv. Funct. Mater..

[cit210] Luo J., Gao S., Luo H., Wang L., Huang X., Guo Z., Lai X., Lin L., Li R. K., Gao J. (2021). Chem. Eng. J..

